# Integrative Bioinformatics Study of Tangeretin Potential Targets for Preventing Metastatic Breast Cancer

**DOI:** 10.1155/2021/2234554

**Published:** 2021-07-13

**Authors:** Adam Hermawan, Herwandhani Putri, Naufa Hanif, Muthi Ikawati

**Affiliations:** ^1^Laboratory of Macromolecular Engineering, Department of Pharmaceutical Chemistry, Faculty of Pharmacy, Universitas Gadjah Mada Sekip Utara II, Yogyakarta 55281, Indonesia; ^2^Cancer Chemoprevention Research Center, Faculty of Pharmacy, Universitas Gadjah Mada Sekip Utara II, Yogyakarta 55281, Indonesia

## Abstract

Agents that target metastasis are important to improve treatment efficacy in patients with breast cancer. Tangeretin, a citrus flavonoid, exhibits antimetastatic effects on breast cancer cells, but its molecular mechanism remains unclear. Tangeretin targets were retrieved from PubChem, whereas metastatic breast cancer regulatory genes were downloaded from PubMed. In total, 58 genes were identified as potential therapeutic target genes of tangeretin (PTs). GO and KEGG pathway enrichment analyses of PTs were performed using WebGestalt (WEB-based Gene SeT AnaLysis Toolkit). The PPI network was analyzed using STRING-DB v11.0 and visualized by Cytoscape software. Hub genes were selected on the basis of the highest degree score as calculated by the CytoHubba plugin. Genetic alterations of the PTs were analyzed using cBioPortal. The prognostic values of the PTs were evaluated with the Kaplan–Meier plot. The expression of PTs across breast cancer samples was confirmed using GEPIA. The reliability of the PTs in metastatic breast cancer cells was validated using ONCOMINE. Molecular docking was performed to foresee the binding sites of tangeretin with PIK3C*α*, MMP9, PTGS2, COX-2, and IKK. GO analysis showed that PTs participate in the biological process of stimulus response, are the cellular components of the nucleus and the membrane, and play molecular roles in enzyme regulation. KEGG pathway enrichment analysis revealed that PTs regulate the PI3K/Akt pathway. Genetic alterations for each target gene were *MTOR* (3%), *NOTCH1* (4%), *TP53* (42%), *MMP9* (4%), *NFKB1* (3%), *PIK3CA* (32%), *PTGS2* (15%), and *RELA* (5%). The Kaplan–Meier plot showed that patients with low mRNA expression levels of *MTOR, TP53, MMP9, NFKB1, PTGS2,* and *RELA* and high expression of *PIK3CA* had a significantly better prognosis than their counterparts. Further validation of gene expression by using GEPIA revealed that the mRNA expression of *MMP9* was significantly higher in breast cancer tissues than in normal tissues, whereas the mRNA expression of *PTGS2* showed the opposite. Analysis with ONCOMINE demonstrated that the mRNA expression levels of *MMP9* and *NFKB1* were significantly higher in metastatic breast cancer cells than in normal tissues. The results of molecular docking analyses revealed the advantage of tangeretin as an inhibitor of PIK3CA, MMP9, PTGS2, and IKK. Tangeretin inhibits metastasis in breast cancer cells by targeting TP53, PTGS2, MMP9, and PIK3CA and regulating the PI3K/Akt signaling pathway. Further investigation is needed to validate the results of this study.

## 1. Introduction

Breast cancer is a common cause of death among women worldwide [1]. Breast cancer was initially considered a local disease, but it can metastasize to lymph nodes and other organs in the body, which is fatal to patients [2]. In breast cancer patients, metastases are still the leading cause of morbidity [3]. Understanding the molecular mechanisms underlying metastasis is important to improve the clinical management of breast cancer [4]. Accordingly, molecular therapeutic agents that target metastasis must be developed to enhance the effectiveness of breast cancer therapy [5].

**Figure 1:**
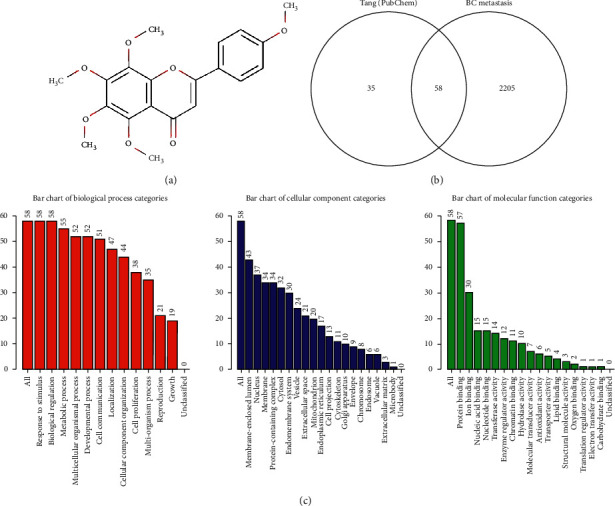
(a) Chemical structure of tangeretin. (b) A Venn diagram between tangeretin targets and regulatory genes of breast cancer metastasis. (c) GO enrichment, as analyzed by WebGestalt.

Tangeretin, a citrus flavonoid (Figure 1(a)), may be developed as a specific molecular-targeted anticancer agent because of its antimetastatic effects [6] on cancer cells [7–9]. Specifically, this compound inhibits metastases of skin, breast, and gastric cancer cells. Tangeretin hampers the invasion of MO4 mouse cells into the embryonic chick heart [10]. It also inhibits lung metastasis in melanoma B16F10 cell xenografts [11] and metastasis in 7,12-dimethylbenz (*α*) anthracene-induced rat breast cancer [12]. Moreover, tangeretin alleviates epithelial-mesenchymal transition (EMT), invasion, and migration in gastric cancer cells by downregulating Notch-1, Jagged1/2, Hey-1, and Hes-1 [13]. Nonetheless, the molecular target of tangeretin for the metastatic inhibition of breast cancer remains unknown.

In this study, we used a bioinformatics approach to obtain tangeretin protein target data from PubChem, metastatic breast cancer regulatory genes from PubMed, and potential target genes of tangeretin against metastatic breast cancer (PT). We performed gene ontology (GO), Kyoto Encyclopedia of Genes and Genomes (KEGG) pathway enrichment, and protein-protein interaction (PPI) network analyses and selected hub genes on the basis of the highest degree score. Selected PTs were further analyzed for their prognostic values by using Kaplan–Meier survival plots and GEPIA. Corroboration of the accuracy of the selected PT in metastatic breast cancer samples was performed using ONCOMINE. Alterations in the selected genes were analyzed using the public database cBioPortal. Molecular docking studies were conducted to identify the interaction between tangeretin and PT. The results of this study emphasized the potential of tangeretin as an antimetastatic agent in breast cancer therapy.

## 2. Materials and Methods

### 2.1. Data Collection and Processing

We downloaded 95 tangeretin targets from PubChem (Supplementary Table 1) and 2263 metastatic breast cancer regulatory genes from PubMed (Supplementary Table 2). A Venn diagram was generated using the tangeretin targets from PubChem and the metastatic breast cancer regulatory genes from PubMed, which resulted in 58 genes considered potential therapeutic target genes of tangeretin (PTs) (Figure 1(b), Supplementary Table 3).

### 2.2. GO and KEGG Pathway Enrichment Analyses

GO and KEGG pathway enrichment analyses were performed using WebGestalt (WEB-based Gene SeT AnaLysis Toolkit) with *p* < 0.05 as the cutoff value [14].

### 2.3. PPI Network and Selection of Hub Genes

The PPI network was analyzed using STRING-DB v11.0 [15] with confidence scores of >0.7 and visualized by Cytoscape software [16]. Hub genes were selected on the basis of the highest degree score as calculated by the CytoHubba plugin [17].

### 2.4. Genetic Alteration Analysis of PTs

Genetic alterations of the PTs were analyzed using cBioPortal [18, 19]. Further connectivity analysis was performed to PTs by using the selected breast cancer study, with a cutoff value of *p* < 0.05.

### 2.5. Kaplan–Meier Survival Analysis

The prognostic values of the PTs were evaluated with the Kaplan–Meier plot (http://kmplot.com) by using the breast cancer database. The cutoff value was *p* < 0.05 [20], and the number of samples is displayed in each curve.

### 2.6. Validation of PTs in Breast Cancer and Metastatic Breast Cancer Samples

The expression of PTs across breast cancer samples from TCGA and GTEx projects was confirmed using GEPIA (http://gepia.cancer-pku.cn), with a cutoff value of *p* < 0.05 [21]. The reliability of the PTs in metastatic breast cancer cells was validated by ONCOMINE (https://www.oncomine.org) [22] using samples from some projects, including TCGA, a study by Finak et al. [23], a study by Sorlie et al. [24] and a study by Perou et al. [25].

### 2.7. Molecular Docking

Molecular docking was performed to foresee the binding sites of tangeretin with PIK3C*α* (PDB ID: 4OVV), MMP9 (PDB ID: 2OW1), PTGS2 (PDB ID: 5F1A), COX-2 (PDB ID: 6COX), and IKK (PDB ID: 4KIK). All computational analyses were conducted using Windows 10 with an Intel Core i5-7th Gen processor and 4 GB RAM. The docking simulation, RMSD calculation, and visualization interaction were conducted using MOE 2010 (Licensed from Faculty of Pharmacy UGM). The structure of tangeretin was downloaded from PubChem (https://pubchem.ncbi.nlm.nih.gov) and sought for conformation and minimization by MOE using the energy minimization module. Docking simulations were performed on the binding side of the native ligand based on flexible ligand structures and rigid receptors. The London dG and triangle matchers were selected for the score function and placement settings, respectively, in the docking simulation. The forcefield method was used to refine the docking results of 30 settings. Docking simulation was performed using the default settings. The analysis results will conclude in which conformations generate the lowest energy when tangeretin binds to the target protein.

## 3. Results

### 3.1. GO and KEGG Pathway Enrichment Analyses

Metastasis is the main cause of death in patients with breast cancer. Utilizing the bioinformatics approach, we identified the PTs and mechanisms of tangeretin in inhibiting metastatic breast cancer. GO analysis was conducted with WebGestalt on the basis of three criteria, namely, biological process, cellular component, and molecular function (Figure 1(c)). PTs participate in the biological processes of stimulus response, metabolic process, and cell proliferation. In addition, PTs are cellular components of the nucleus and the membrane. PTs also play a molecular role in protein binding, ion binding, and enzyme regulator activity. Pathway enrichment by KEGG of the PTs (Supplementary Table 3) showed the regulation of ∼106 pathways, including the PI3K-Akt, breast cancer, and TNF signaling pathways (Supplementary Table 4), three main pathways that are regulated by tangeretin in metastasis signaling, based on the literature study. Several PTs were involved in PI3K-Akt signaling (e.g., PIK3CA, PRKAA2, RELA, and TP53), the breast cancer pathway (e.g., AKT1, MTOR, NOTCH1, PIK3CA, and TP53), and the TNF signaling pathway (e.g., MMP9, NFKB1, PIK3CA, PTGS2, and RELA) (Supplementary Table 5).

### 3.2. Analysis of the PPI Network and Selection of Hub Genes

**Figure 2:**
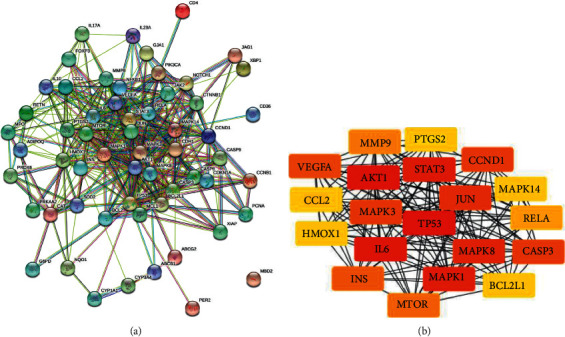
(a) Protein-protein interaction network of potential target genes of tangeretin against metastatic breast cancer, analyzed by STRING. (b) Top 20 hub genes based on the highest degree score, analyzed by CytoHubba.

A PPI network was constructed from 58 proteins (confidence level of 0.4) consisting of 58 nodes, 409 edges, PPI enrichment value of <1.10*e* − 16, and average local clustering coefficient of 0.62 (Figure 2(a)). The top 20 highest degree score genes, also known as hub genes, were identified, including TP53, AKT1, STAT3, IL6, and MAPK1 (Figure 2(b), Table 1).

### 3.3. Analysis of Genetic Alterations of Potential Target Genes

**Figure 3:**
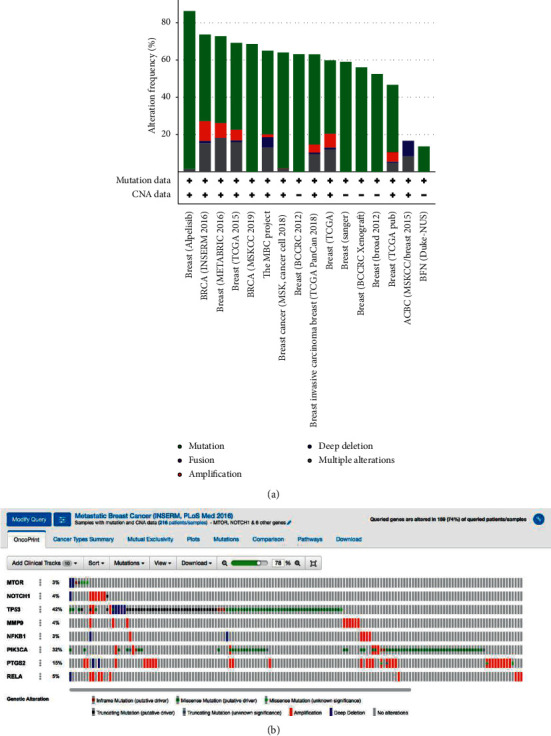
(a) Overview of genetic changes in *MTOR, NOTCH1, TP53, MMP9, NFKB1, PIK3CA, PTGS2,* and *RELA* across 16 breast cancer studies, as analyzed by cBioportal. (b) Summary of alterations in *MTOR, NOTCH1, TP53, MMP9, NFKB1, PIK3CA, PTGS2,* and *RELA* across breast cancer patients using a study from Lefebvre et al. [26]

Eight PTs were analyzed using cBioportal to explore their genomic alterations across breast cancer studies. *MTOR*, *NOTCH1, PIK3CA, TP53, MMP9, NFKB1, PTGS2,* and *RELA* were selected from KEGG pathway enrichment (Supplementary Table 5), whereas *TP53, MTOR, MMP9,RELA,* and *PTGS2* were selected based on the highest degree score using CytoHubba. The study BRCA INSERM 2016 [26] was selected for further analysis (Figure 3(a)). Genetic alterations for each target gene ranged from 3% to 42% of samples, including *MTOR* (3%), *NOTCH1* (4%), *TP53* (42%), *MMP9* (4%), *NFKB1* (3%), *PIK3CA* (32%), *PTGS2* (15%), and *RELA* (5%) (Figure 3(b)). Moreover, most gene alterations belonged to amplification, missense mutation, and truncating mutation (Figure 3(b)). Further analysis of mutual exclusivity showed that only one gene pair (*NOTCH1-RELA*) exhibited significant co-occurrence (*p* < 0.05) in the breast cancer study by the INSERM 2016 project (Table 2), which indicated the pivotal role of NOTCH1 and RELA under tangeretin treatment.

### 3.4. Kaplan–Meier Survival Analysis

**Figure 4:**
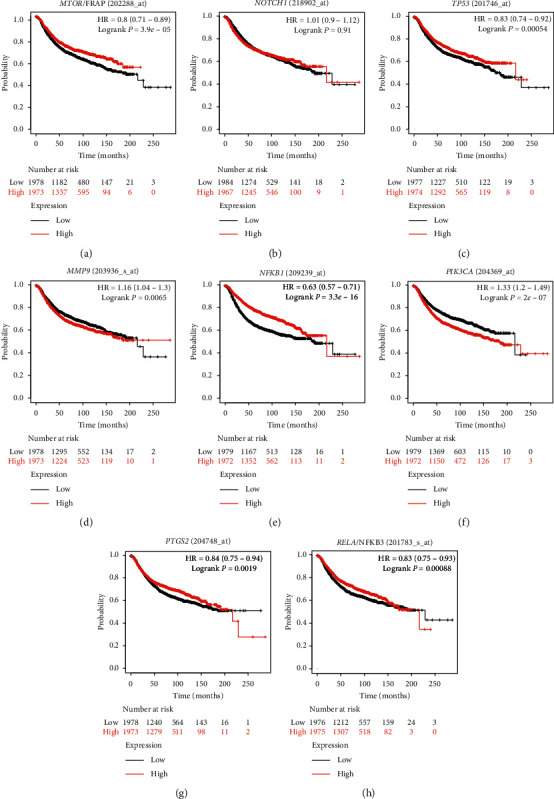
Overall survival of patients with breast cancer related to the mRNA levels of *MTOR, NOTCH1, TP53, MMP9, NFKB1, PIK3CA, PTGS2,* and *RELA*, as analyzed by GEPIA. (a) *MTOR*/FRAP (202288_at). (b) *NOTCH1* (218902_at). (c) *TP53* (201746_at). (d) *MMP9* (203936_s_at). (e) *NFKB1* (209239_at). (f) *PIK3CA* (204369_at). (g) *PTGS2* (204748_at). (h) *RELA*/NFKB3 (201783_s_at).

The Kaplan–Meier plot showed that patients with low mRNA expression levels of *MTOR*(*p*=3.95 × 10^−5^) , *TP53*(*p*=0.00054), *MMP9*(*p*=0.0065), *NFKB1*(*p*=3.3 × 10^−16^), *PTGS2*(*p*=0.0019), and *RELA*(*p*=0.00088) had significantly better overall survival rates than the opposite group (Figure 4). In addition, patients with a low mRNA level of *NOTCH1* had better overall survival rates than those with a high mRNA level of *NOTCH1*, but the difference was not significant (*p*=0.91). Moreover, patients with a low mRNA expression of *PIK3CA* showed significantly worse overall survival than the opposite group (*p*=2 × 10^−7^).

### 3.5. Validation of PTs in Breast Cancer and Metastatic Breast Cancer Samples

**Figure 5:**
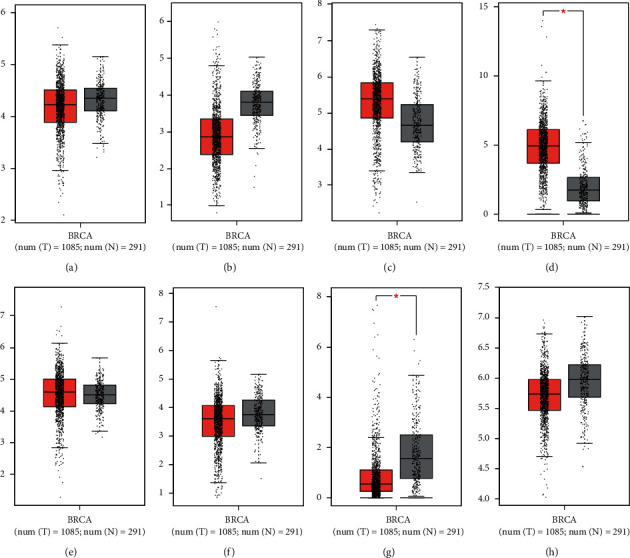
mRNA levels of (a) *MTOR*, (b) *NOTCH1*, (c) *TP53*, (d) *MMP9*, (e) *NFKB1*, (f) *PIK3CA*, (g) *PTGS2*, and (h) *RELA* in patients with breast cancer, as analyzed by GEPIA.

**Figure 6:**
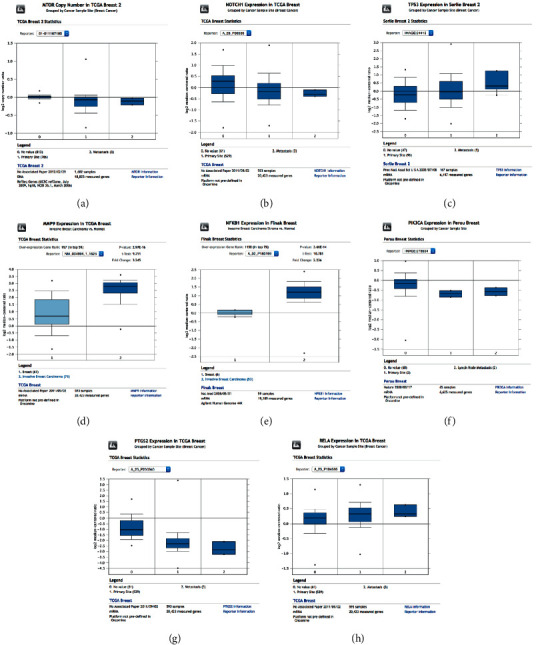
mRNA levels of (a) *MTOR*, (b) *NOTCH1*, (c) *TP53*, (d) *MMP9*, (e) *NFKB1*, (f) *PIK3CA*, (g) *PTGS2*, and (h) *RELA* in patients with metastatic breast cancer, as analyzed by ONCOMINE.

Validation of PTs in TCGA and GTEx samples using GEPIA demonstrated that the mRNA expression of *MMP9* was significantly higher in breast cancer tissues than in normal tissues (Figure 5). In addition, the mRNA expression of *PTGS2* was significantly lower in breast cancer tissues than in normal tissues. Furthermore, no significant difference in the mRNA expression levels of *MTOR, NOTCH1, TP53, NFKB1, PIK3CA,* and *RELA* was observed between breast cancer and normal tissue samples. The validation of target genes by using ONCOMINE showed that, in samples from a TCGA study, the mRNA level of *MMP9* was significantly higher in metastatic breast cancer cells than in normal breast cells with *p*=2.97 × 10^−16^ (Figure 6). In addition, samples from a study by Finak et al. showed that the mRNA level of *NFKB1* was significantly higher in metastatic breast cancer cells than in normal breast cells (*p*=3.66 × 10^−14^) [23]. Moreover, the mRNA levels of *MTOR, NOTCH1, TP53, PIK3CA, PTGS2,* and *RELA* were not different between metastatic breast cancer cells and normal breast cells.

### 3.6. Molecular Docking

**Figure 7:**
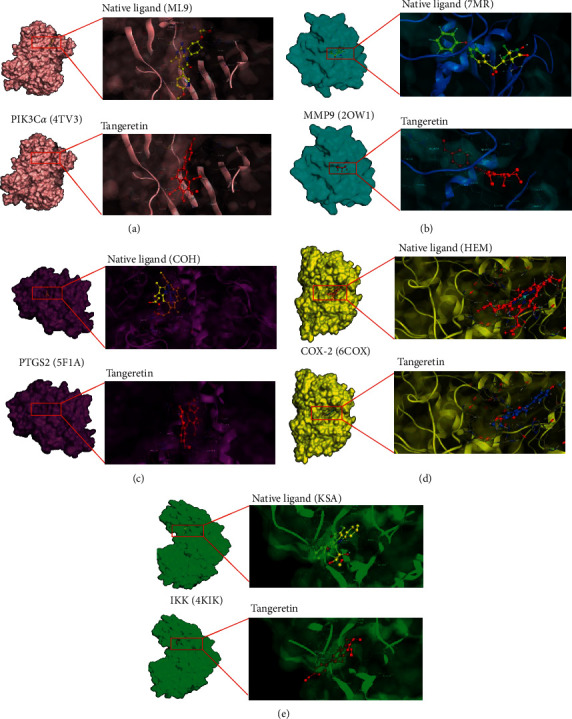
Visualization of ligand interaction to PIK3C*α*, MMP9, PTGS2, and IKK using MOE.

Simulation of molecular docking and visualization of ligand-protein binding were conducted with MOE software. The protein targets, including PIK3C*α*, MMP9, PTGS2, COX-2, and IKK, were selected on the basis of KEGG pathway enrichment analysis, hub gene selection, survival analysis, PT validation, and uniqueness as drug targets through literature research. Native ligands of each protein consist of PIK3C*α*, MMP9, PTGS2, COX-2, and IKK complexes comprising ML9 (2-amino-8-[trans-4-(2-hydroxyethoxy)cyclohexyl]-6-(6-methoxypyridin-3-yl)-4-methylpyrido[2,3-d]pyrimidin-7(8H)-one), 7 MR ((2R)-2-amino-3,3,3-trifluoro-n-hydroxy-2-{[(4-phenoxyphenyl) sulfonyl] methyl}propanamide), COH (protoporphyrin IX containing CO), HEM (protoporphyrin IX containing Fe), and KSA (K-252A). PIK3C*α* and MMP9 showed slightly lower docking scores than native ligands (ML9 and 7 MR) (Table 3). The lower the docking score, the more potent the binding affinity of the ligand, implying that PIK3C*α* and MMP9 tend to bend and react with tangeretin. Furthermore, tangeretin formed arene-H between Ile932 and the compound with a bonding distance of 4.07, which was shorter than the arene-H distance of ML9 with Ile932 (4.22) (Figure 7). The higher docking score of tangeretin on PTGS2, COX-2, and IKK indicated lower binding affinity compared with native ligands (3X). This phenomenon can be ascribed to the fact that only one amino acid, Gln203, interacted with tangeretin on PTGS2 by an arene-H bond (Table 3). Otherwise, the native ligand of PTGS2 (COH) had four amino acids, which interacted through arene-H (Gln203, Leu391), arene-cation (His207), and metal (His214) (Table 3). A similar phenomenon occurred for 6COX and IKK; the amino acid that interacted with tangeretin was fewer than the native ligands (Table 3).

## 4. Discussion

Metastasis is the main cause of death in patients with breast cancer. Utilizing a bioinformatics approach, we identified the PTs and mechanisms of tangeretin in inhibiting metastatic breast cancer. This study emphasized the important role of the PI3K/Akt pathway and related genes (*TP53, PTGS2, NFKB1,* and *PIK3CA*) in the antimetastatic effects of tangeretin on metastatic breast cancer cells. Here, we discussed the important roles of those genes and their potential as tangeretin targets against metastatic breast cancer cells. *TP53* encodes the tumor protein p53, a tumor suppressor gene [27]. Mutations in *TP53* occur in human epidermal growth factor receptor 2-positive [27], estrogen receptor-positive, and progesterone-positive breast cancer subtypes [28]. In addition, the *TP53* gene is mutated in 80% of patients with triple-negative breast cancer [29]. Loss of *p53* or gain of mutant *p53* promotes tumor progression and metastasis [30]. In addition, loss of *p53* induces metastasis via activation of Wnt signaling [31]. Moreover, the mutation in *TP53* can promote immunogenic activity in breast cancer [32].

Tangeretin regulates *p53* expression. Tangeretin increases *p53* expression in AGS human gastric cancer cells [33]. In addition, tangeretin treatment induces the upregulation of *p53* and inhibits metastasis in 7,12-dimethylbenz (*α*) anthracene-induced rat breast tumors [12]. However, the study of *TP53* mutation, metastasis, and tangeretin treatment remains elusive.

*MMP9* encodes matrix metalloproteinase 9 *(MMP9),* a protease that cleaves the extracellular matrix and is involved in angiogenesis, invasion, and metastasis [34]. *MMP9* is dominantly synthesized by tumor cells [35]. *MMP9* is upregulated in breast cancer cells compared with normal tissue and is correlated with metastasis and recurrence in breast cancer [36]. Thus, inhibition of MMP activity is an effective way of preventing metastasis in patients with breast cancer [37]. A previous study demonstrated that tangeretin inhibits metastasis in rat mammary carcinoma induced by 7,12-dimethylbenz (*α*) anthracene by downregulating *MMP2, MMP9,* and *VEGF* [12]. In addition, tangeretin inhibits the expression and activity of MMP9 in rats with pilocarpine-induced seizures [38]. Future studies of the effect of tangeretin on *MMP9* activity in metastatic breast cancer are warranted.

*PTGS2* encodes prostaglandin-endoperoxide synthase 2, also known as cyclooxygenase-2 (COX-2), which participates in prostaglandin synthesis, regulates inflammation, and promotes cancer progression, invasion, and migration [39,40]. COX-2 is expressed in 40% of human metastatic breast cancers. [41]. A previous study showed that tangeretin inhibits COX-2 expression induced by IL-1beta in A549 lung cancer cells by inhibiting NF-kB, p38 MAPK, JNK, and PI3K signaling [42]. Moreover, tangeretin inhibits UVB-induced COX-2 expression by inhibiting MAPK activation and reactive oxygen species elevation [43]. Recently, an in silico study demonstrated that tangeretin can inhibit C0X-2 [44]. Nevertheless, the effects of tangeretin on COX-2 activity and expression in metastatic breast cancer cells need further exploration.

*NFKB1* encodes the nuclear factor of kappa light polypeptide gene enhancer in B-cells 1, a member subunit of NKFB [45]. NFKB1 forms various dimeric complexes with other subunits to activate NFkB signaling that regulates several biological processes, including inflammation, senescence, apoptosis, cell survival, and cancer progression [45]. NFKB1 plays a role in cancer progression and is a potential target for cancer therapy [46]. NFkB signaling is important in the invasiveness of inflammatory breast cancer [47], as well as in chemoresistance mechanisms and invasive breast cancer [48]. Moreover, NFKB1/RELA induces breast cancer progression by upregulating ETS1 [49]. A previous study showed that tangeretin treatment reduces the phosphorylation of I*κ*B-*α* and IKK-*β*, as well as the nuclear translocation of the p65 subunit of NF-*κ*B in lipopolysaccharide-stimulated microglial cells [50]. Hence, the inhibitory effect of tangeretin on invasion and metastasis by targeting NFkB signaling needs to be explored in future studies.

*PIK3CA* encodes phosphatidylinositol-4,5-bisphosphate 3-kinase catalytic subunit alpha (PIK3CA), also known as p110*α*, a member of the phosphoinositide 3-kinase (PI3K) family [51]. The PI3K signaling pathway is involved in the biological processes of cellular proliferation, apoptosis, survival, motility, and metastasis [52,53]. Mutation in PIK3CA is present in most solid tumors [54] and is found in 12%–15% of patients with breast cancers [55]. A recent study has shown that mutation in PIK3CA corresponds to a poor prognosis in patients with hormone receptor-positive metastatic breast cancer but a good prognosis in patients with triple-negative breast cancer [56].

Tangeretin inhibits the PI3K signaling pathway. Tangeretin enhances the sensitivity of human ovarian cancer cells to cisplatin by downregulating the PI3K/Akt signaling pathway [57]. Tangeretin also inhibits the proliferation and migration of aortic smooth muscle cells by suppressing the PI3K/AKT signaling pathway [58]. Another study showed that tangeretin poses potent neuroprotective activity by triggering the PI3K/Akt signaling pathway in pilocarpine-induced seizures rats [38]. Tangeretin also inhibits PI3K and Notch signaling in neonatal asthmatic mice [59]. Moreover, tangeretin inhibits EMT in PC-3 prostate cancer cells by downregulating the PI3K/Akt/mTOR pathway [60]. However, the effects of tangeretin on PI3K signaling and PIK3CA mutation on metastatic breast cancer cells need to be clarified in future studies.

KEGG pathway enrichment analysis indicated that PTs regulate the PI3K/Akt signaling pathway. In this study, we discussed the cross-talk between PTs in the regulation of the PI3K/Akt pathway. COX2 promotes cell survival by activating the PI3K/Akt pathway in human lung cancer cells [61]. Inhibition of COX2 blocks PI3K/AKT kinase activity in ovarian cancer [62] and hepatocellular carcinoma cells [63]. In addition, PI3K/Akt kinase activity induces COX2 expression in lipopolysaccharide-induced murine adrenocortical cells [64]. Furthermore, COX2 and PI3K are associated with the progression of colon cancer [65].

The PI3K/Akt and mTOR signaling pathways are essential for maintaining the proliferation and survival of cancer cells [66]. A recent study has shown that activation of PI3K/AKT/mTOR signaling increases hepatocellular carcinoma resistance to radiotherapy [67]. On the one hand, activation of the PI3K/Akt pathway leads to the transcriptional activity of NF*κ*B [68]. On the other hand, NF*κ*B activity is important for oncogenic transformation induced by PI3K/Akt signaling [68]. Mutations in PI3K signaling regulators, including PIK3CA, lead to cytokine expression upon growth factor deprivation in an NF*κ*B-dependent manner [69]. Furthermore, PI3K/Akt/JNK/NF*κ*B signaling plays a pivotal role in the expression of MMP-9 and enlargement in human limbal epithelial cells [70].

Activation of the PI3K/PTEN/AKT/mTOR pathway promotes invasion and metastasis by increasing the expression of MMP9 in hepatocellular carcinoma cells [71] and human breast cancer cells [72]. Furthermore, inhibition of Notch1 signaling reduces the proliferation, migration, and invasion of human breast cancer cells by decreasing PI3K/Akt activity (Li et al.). p53 participates in the regulation of cell survival by blocking the PI3K/AKT signaling pathway in cancer cells [73]. Moreover, activating mutations in PIK3CA promote the stimulation of p53 signaling [74]. A previous study showed that PI3K/Akt promotes p53 translation in cancer development [75]. Inhibition of PI3K/Akt signaling leads to p53 upregulation in leukemic cancer cells [76]. A recent study reported p53 upregulation due to PI3K/Akt signaling inhibition in EMT inhibition in liver cancer cells [77].

In this study, molecular docking analysis emphasized the potential target of tangeretin in inhibiting metastatic breast cancer cells. Tangeretin was shown to inhibit PIK3CA, MMP9, COX2, and IKK. One of the unique targets for cancer drug discovery is PIK3C*α* because of the high prevalence of its mutations in various human tumors and the progression in the development of personalized cancer medicines [78]. The docking results on PIK3CA showed that the docking score of tangeretin was slightly lower than that of the native ligand ML9 ((2-amino-8-[trans-4-(2-hydroxyethoxy) cyclohexyl]-6-(6-methoxypyridin-3-yl)-4-methylpyrido [2,3-d]pyrimidin-7(8H)-one)). A low docking score represents a potent affinity of binding of the ligand, indicating that PIK3C*α* tends to bend and react with tangeretin instead of the native ligand. The docking results of tangeretin on PIK3CA formed arene-H between Ile932 and the compound with a bonding distance of 4.07, which was shorter than the arene-H distance of ML9 with Ile932 (4.22) (Table 3). Furthermore, tangeretin has donor sidechains, whereas native ligands have donor-acceptor backbones. Hence, this donor sidechain is useful in increasing tangeretin binding to PIK3CA. The docking results on MMP9 showed that the docking score of tangeretin was lower than that of the native ligand 7 MR ((2R)-2-amino-3,3,3-trifluoro-n-hydroxy-2-{[(4-phenoxyphenyl) sulfonyl] methyl}propanamide)). This result is due to the differences in bond types. Specifically, the native ligand has a type of arene-arene bond, whereas tangeretin has backbone and sidechain donors. This result is in line with the findings of Roshini et al. that tangeretin, when combined with zinc oxide (Tan-ZnO QDs), can downregulate the expression of metastasis markers, such as MMP2, MMP9, and VEGF [79]. Tangeretin showed a higher docking score than native ligands on PTGS2, COX-2, and IKK, suggesting that tangeretin has a lower binding affinity than the native ligands COH (protoporphyrin IX containing CO), HEM (protoporphyrin IX containing Fe), and KSA (K-252A) (Table 3).

Molecular docking results on PTGS2 showed that only one amino acid, Gln203, interacted with tangeretin by an arene-H bond (Table 3). Otherwise, the native ligand of PTGS2 (COH) had four amino acids, which are interacted by arene-H (Gln203, Leu391), arene-cation (His207), and metal contact (His214) (Table 3). The results of molecular docking on COX2 showed a lower docking score of tangeretin than native ligands because fewer amino acids on 6COX interacted with tangeretin than native ligands.

The IKK complex plays a pivotal role in NFkB signaling and is an important target for cancer therapy [80, 81]. Molecular docking results on IKK with the PDB code 4KIK showed that the docking scores of tangeretin were lower than those of native ligands because of the lack of one type of bonding, namely, backbone acceptor. However, tangeretin still inhibited COX2 and IKK activities. These results are supported by the previous finding of Chen et al. that tangeretin inhibits IL-1*β*-induced COX-2 protein expression by suppressing COX-2 gene expression [42]. Another study also showed that tangeretin significantly inhibits the activation of IKK-*β* induced by LPS [50]. Altogether, although the binding affinity of tangeretin is not much more robust than native ligands, it still has the potency to inhibit PTGS2, COX2, and IKK activities. Collectively, the PI3K/Akt signaling pathway is important for the regulation of metastatic breast cancer and is a potential target of tangeretin in inhibiting metastasis. However, whether the inhibitory effect of tangeretin on PI3K/Akt signaling is related to metastatic breast cancer requires further exploration.

## 5. Conclusions

Tangeretin inhibits metastasis in breast cancer cells by targeting TP53, PTGS2, MMP9, and PIK3CA. Molecular docking studies revealed the potential of tangeretin as an inhibitor of MMP9 and PTGS2. Furthermore, PI3K/Akt signaling is a potential target of tangeretin in inhibiting breast cancer metastasis. Future *in vitro* and *in vivo* investigations are needed to validate the results of this study.

## Figures and Tables

**Table 1:** Top 20 hub genes ranked by degree method, analyzed by CytoHubba.

Rank	Gene symbol	Gene name	Score
1	TP53	Cellular tumor antigen p53	40
2	AKT1	RAC-alpha serine/threonine-protein kinase	37
3	IL6	Interleukin-6	33
4	STAT3	Signal transducer and activator of transcription 3	31
4	MAPK1	Mitogen-activated protein kinase 1	31
6	MAPK8	Mitogen-activated protein kinase 8	29
7	JUN	Transcription factor AP-1	27
8	CCND1	G1/S-specific cyclin-D1	26
8	CASP3	Caspase-3	26
8	MAPK3	Mitogen-activated protein kinase 3	26
11	VEGFA	Vascular endothelial growth factor A	24
12	INS	Insulin	23
13	MTOR	Serine/threonine-protein kinase mTOR	22
14	MMP9	Matrix metalloproteinase-9	21
15	RELA	Transcription factor p65	20
16	BCL2L1	Bcl-2-like protein 1	19
16	CCL2	C-C motif chemokine 2	19
16	MAPK14	Mitogen-activated protein kinase 14	19
16	PTGS2	Prostaglandin G/H synthase 2	19
16	HMOX1	Heme oxygenase 1	19

**Table 2:** Mutual exclusivity analysis of selected genes.

A	B	Log2 odds ratio	*p* value	Tendency
NOTCH1	RELA	>3	<0.001	Co-occurrence

**Table 3:** Molecular docking results of tangeretin against the protein targets of PIK3C*α*, MMP9, PTGS2, COX-2, and IKK.

Protein	Ligand native						Tangeretin				
	Docking score	RMSD (Å)	Ligand atom	Amino acid	Binding type	Distance	Docking score	Ligand atom	Amino acid	Binding type	Distance
PIK3C*α* (4TV3)	−12.3229	1.9820	C	Ile848	Arene-H	4.81	−13.0943	C	Ile932	Arene-H	4.07
			C	Ile932	Arene-H	4.22		C	Trp780	Arene-H	7.51
			N	Val851	Backbone donor-acceptor	4.10		O	Lys802	Sidechain donor	5.20

MMP9 (2OW1)	−11.4732	1.7393	C	Leu188	Arene-H	5.02	−11.5442	C	Arg424	Arene-H	6.41
			C	Tyr423	Arene-H	4.66		O	Tyr423	Backbone donor	5.82
			C	Leu418	Arene-H	4.10		C	Leu418	Arene-H	4.42
			C	7MR502	Arene-arene			O	Gln402	Sidechain donor	4.16

PTGS2 (5F1A)	−14.8424	1.2559	C	Gln203	Arene-H	3.61	−11.8904	C	Gln203	Arene-H	4.39
			C	Leu391	Arene-H	6.85					
			O-	His207	Arene-cation	4.37					
			O	His214	Metal contact	5.61					

COX-2 (6COX)	−15.6490	1.0546	O	Asn382	Sidechain donor	5.26	−12.0495	C	His386	Arene-H	5.12
			O	Thr212	Backbone donor	3.52		O	Gln203	Arene-H	4.73
			O-<	Gln454	Sidechain donor	5.73		C	Leu391	Arene-H	6.40
			O	His214	Metal contact	5.00					

IKK (4KIK)	−14.0211	0.8232	C	Ile165	Arene-H	3.89	−10.4698	C	Ile165	Arene-H	4.67
			C	Leu21	Arene-H	3.72		O	Leu21	Arene-H	6.94
			O	Cys99	Backbone donor	3.66		O	Cys99	Backbone donor	4.00
			C	Val152	Arene-H	6.07		O	Val152	Arene-H	6.05
			C	Val29	Arene-H	5.80		C	Asp103	Arene-H	4.40
			N	Glu97	Backbone acceptor	6.18					

## Data Availability

All data produced by the study are included within the manuscript and supplementary information files.

## Abbreviations

COX2: Cyclooxygenase 2
EMT: Epithelial-to-mesenchymal transition
GO: Gene Ontology
KEGG: Kyoto Encyclopedia of Genes and Genomes
MMP9: Matrix metalloproteinase 9
NFkB1: Nuclear factor of kappa light polypeptide gene enhancer in B-cells 1
PIK3CA: Phosphatidylinositol-4,5-bisphosphate 3-kinase catalytic subunit alpha
PPI: Protein-protein interaction
PT: Potential therapeutic target genes of tangeretin
PTGS2: Prostaglandin-endoperoxide synthase 2.

## Supplementary Materials

Supplementary Materials Supplementary Table 1. Tangeretin targets in human, as retrieved from PubChem. Supplementary Table 2. Genes related to metastatic breast cancer, as retrieved from PubMed. Supplementary Table 3. Potential therapeutic target genes of tangeretin (PTs). Supplementary Table 4. KEGG pathway enrichment analysis of the PTs. Supplementary Table 5. Gene list enriched in breast cancer, TNF, and PI3K signaling pathway.
