# Supplementary material for: Integrative Bioinformatics Study of Tangeretin Potential Targets for Preventing Metastatic Breast Cancer
Source: Evid Based Complement Alternat Med. 2021 Jul 13;2021:2234554. doi: 10.1155/2021/2234554 (PMC8294962; doi:10.1155/2021/2234554)
Supplement: Supplementary Materials — Supplementary Table 1. Tangeretin targets in human, as retrieved from PubChem. Supplementary Table 2. Genes related to metastatic breast cancer, as retrieved from PubMed. Supplementary Table 3. Potential therapeutic target genes of tangeretin (PTs). Supplementary Table 4. KEGG pathway enrichment analysis of the PTs. Supplementary Table 5. Gene list enriched in breast cancer, TNF, and PI3K signaling pathway. [file 2234554.f1.pdf]

Supplementary Table 1. Tangeretin targets in human, as retreived from PuBChem

| No | Protein name | Symbol |
|---|---|---|
| 1 | Unspecific Monooxygenase | |
| 2 | Proline Rich Protein Bstni Subfamily 1 | PRB1 |
| 3 | Caspase 3 | CASP3 |
| 4 | Tumor Necrosis Factor | CD40LG |
| 5 | Bcl2, Apoptosis Regulator | BCL2 |
| 6 | Cytochrome P450 Family 3 Subfamily A Membe | CYP3A4 |
| 7 | Atp Binding Cassette Subfamily B Member 1 | ABCB1 |
| 8 | Mitogen-Activated Protein Kinase | MAP3K10 |
| 9 | Akt Serine/Threonine Kinase 1 | AKT1 |
| 10 | Glucuronosyltransferase | UGT2A3 |
| 11 | Heme Oxygenase 1 | HMOX1 |
| 12 | Interleukin-6 | IL6 |
| 13 | Mitogen-Activated Protein Kinase 8 | MAPK8 |
| 14 | Inositol-3-Phosphate Synthase | ISYNA1 |
| 15 | Bcl2 Like 1 | BCL2L1 |
| 16 | Glutathione Transferase | GSTO1 |
| 17 | Mitogen-Activated Protein Kinase 3 | MAPK3 |
| 18 | Phosphatidylinositol-4-Phosphate 3-Kinase | PIK3 |
| 19 | Prostaglandin-Endoperoxide Synthase 2 | PTGS2 |
| 20 | Tyrosinase | TYR |
| 21 | Caspase 9 | CASP9 |
| 22 | Protein Kinase X-Linked | PRKX |
| 23 | Proline-Rich P65 Protein | RELA |
| 24 | Cytochrome P450 Family 1 Subfamily A Membe | CYP1A2 |
| 25 | Phosphatidylinositol-4,5-Bisphosphate 3-Kinas | PIK3CA |
| 26 | Jagged 1 | JAG1 |
| 27 | Nad(+) Adp-Ribosyltransferase | ART1 |
| 28 | Cadherin 1 | CDH1 |
| 29 | Mitogen-Activated Protein Kinase Erk-A | ERK |
| 30 | P53 | TP53 |
| 31 | Nad(P)H Dehydrogenase (Quinone) | NQO2 |
| 32 | Notch 1 | NOTCH1 |
| 33 | Cyclin B1 | CCNB1 |
| 34 | Peroxiredoxin 6 Pseudogene 2 | PRDX6 |
| 35 | Mcl1, Bcl2 Family Apoptosis Regulator | MCL1 |
| 36 | Superoxide Dismutase | SOD2 |
| 37 | Insulin | INS |
| 38 | Interleukin 10 | IL10 |
| 39 | Cytochrome P450 Family 1 Subfamily A Membe | CYP1A1 |
| 40 | Methyl-Cpg Binding Domain Protein 2 | MBD2 |
| 41 | Interleukin 17a | IL17A |
| 42 | Caspase 8 | CASP8 |

| | | |
|---|---|---|
| 43 | Poly (Adp-Ribose) Polymerase | PARP |
| 44 | Vascular Endothelial Growth Factor A | VEGFA |
| 45 | Proliferating Cell Nuclear Antigen | PCNA |
| 46 | Catalase | CAT |
| 47 | Udp Glucuronosyltransferase Family 1 Membe | UGT1A9 |
| 48 | Macrophage Scavenger Receptor 1 | MSR1 |
| 49 | Solute Carrier Organic Anion Transporter Famil | SLCO1A2 |
| 50 | Udp Glucuronosyltransferase Family 2 Membe | UGT2B7 |
| 51 | Signal Transducer And Activator Of Transcriptic | STAT3 |
| 52 | C-C Motif Chemokine Ligand 2 | CCL2 |
| 53 | Period Circadian Regulator 2 | PER2 |
| 54 | Myeloperoxidase | MPO |
| 55 | Nuclear Factor Kappa B Subunit 1 | NFKB1 |
| 56 | Quinone Oxidoreductase 1 | CRYZL1 |
| 57 | X-Box Binding Protein 1 | XBP1 |
| 58 | Leucine Aminopeptidase 2 | LAP3P2 |
| 59 | 40s Ribosomal Protein S7 | RPS7 |
| 60 | Glutathione Peroxidase | GPX8 |
| 61 | Mechanistic Target Of Rapamycin Kinase | MTOR |
| 62 | Udp Glucuronosyltransferase Family 1 Membe | UGT1A8 |
| 63 | Interleukin 23 Subunit Alpha | IL23A |
| 64 | Resistin | RETN |
| 65 | X-Linked Inhibitor Of Apoptosis | XIAP |
| 66 | Cadherin 17 | CDH17 |
| 67 | Nad(P)H Quinone Dehydrogenase 1 | NQO1 |
| 68 | Cd36 Molecule | CD36 |
| 69 | Cyclin Dependent Kinase Inhibitor 1a | CDKN1A |
| 70 | Mitogen-Activated Protein Kinase 1 | MAPK1 |
| 71 | Gap Junction Protein Alpha 1 | GJA1 |
| 72 | Forkhead Box P3 | FOXP3 |
| 73 | Cd4 Molecule | CD4 |
| 74 | Janus Kinase 2 | JAK2 |
| 75 | Gelatinase B | MMP9 |
| 76 | Synuclein Alpha | SNCA |
| 77 | Interferon Lambda Receptor 1 | IFNLR1 |
| 78 | Atp Binding Cassette Subfamily G Member 2 (J | ABCG2 |
| 79 | Pyruvate Kinase | PKLR |
| 80 | Protein Kinase Amp-Activated Catalytic Subuni | PRKAA2 |
| 81 | Alpha-Amylase | AMY2A |
| 82 | Polyamine Modulated Factor 1 | PMF1 |
| 83 | Dna Nucleotidylexotransferase | DNTT |
| 84 | Glucose-6-Phosphate Dehydrogenase | G6PD |
| 85 | Cyclin-D1-1 | CCND1 |
| 86 | Taste 2 Receptor Member 14 | TAS2R14 |
| 87 | Apolipoprotein B | APOB |
| 88 | Jun Proto-Oncogene, Ap-1 Transcription Factor | JUN |

| | | |
|---|---|---|
| 89 | Adiponectin, C1q And Collagen Domain Contai | ADIPOQ |
| 90 | Cyclin D | CCND1 |
| 91 | Tyrosine Hydroxylase | TH |
| 92 | Tetraspanin 12 | TSPAN12 |
| 93 | Catenin Beta 1 | CTNNB1 |
| 94 | Mitogen-Activated Protein Kinase 14 | MAPK14 |
| 95 | Phosphatidylinositol 3-Kinase | PI3K |

Supplementary Table 2. Genes related to metastatic breast cancer, as retreived from PubMed

| Symbol | Aliases | description |
|---|---|---|
| BRCA2 | BRCC2, BROVCA2, FACD, FAD, FAD | BRCA2 DNA repair associated |
| BRMS1 | | BRMS1 transcriptional repressor and anoikis regulator |
| BRCA1 | BRCAI, BRCC1, BROVCA1, FANCS, II | BRCA1 DNA repair associated |
| ESR1 | ER, ESR, ESRA, ESTRR, Era, NR3A1 | estrogen receptor 1 |
| TERT | CMM9, DKCA2, DKCB4, EST2, PFBN | telomerase reverse transcriptase |
| CCND1 | BCL1, D11S287E, PRAD1, U21B31 | cyclin D1 |
| ABCG2 | ABC15, ABCP, BCRP, BCRP1, BMDP | ATP binding cassette subfamily G member 2 (Junior blood group |
| FGFR2 | BBDS, BEK, BFR-1, CD332, CEK3, CF | fibroblast growth factor receptor 2 |
| CDKN2A | ARF, CDK4I, CDKN2, CMM2, INK4, I | cyclin dependent kinase inhibitor 2A |
| BCAS3 | GAOB1, MAAB | BCAS3 microtubule associated cell migration factor |
| MALAT1 | HCN, LINC00047, NCRNA00047, NE | metastasis associated lung adenocarcinoma transcript 1 |
| NME1 | AWD, GAAD, NB, NBS, NDKA, NDPI | NME/NM23 nucleoside diphosphate kinase 1 |
| MTA1 | | metastasis associated 1 |
| MTDH | 3D3, AEG-1, AEG1, LYRIC, LYRIC/3D | metadherin |
| KISS1 | HH13, KiSS-1 | KiSS-1 metastasis suppressor |
| PTHLH | BDE2, HHM, PLP, PTHR, PTHRP | parathyroid hormone like hormone |
| BRMS1L | BRMS1 | BRMS1 like transcriptional repressor |
| NCOA3 | ACTR, AIB-1, AIB1, CAGH16, CTG26 | nuclear receptor coactivator 3 |
| CD82 | 4F9, C33, GR15, IA4, KAI1, R2, SAR2 | CD82 molecule |
| MDM4 | HDMX, MDMX, MRP1 | MDM4 regulator of p53 |
| TIAM1 | TIAM-1 | TIAM Rac1 associated GEF 1 |
| SNCG | BCSG1, SR | synuclein gamma |
| MACC1 | 7A5, SH3BP4L | MET transcriptional regulator MACC1 |
| MYC | MRTLC, bHLHe39, c-Myc, MYC | MYC proto-oncogene, bHLH transcription factor |
| RECK | ST15 | reversion inducing cysteine rich protein with kazal motifs |
| IGF1 | IGF, IGF-I, IGFI, MGF | insulin like growth factor 1 |
| CHEK2 | CDS1, CHK2, HuCds1, LFS2, PP1425 | checkpoint kinase 2 |
| TFF1 | BCEI, D21S21, HP1.A, HPS2, pNR-2, | trefoil factor 1 |
| MRTFA | BSAC, MAL, MKL, MKL1, MRTF-A | myocardin related transcription factor A |
| TP53 | BCC7, BMFS5, LFS1, P53, TRP53 | tumor protein p53 |
| MTA2 | MTA1L1, PID | metastasis associated 1 family member 2 |
| MTSS1 | MIM, MIMA, MIMB | MTSS I-BAR domain containing 1 |
| MAP3K1 | MAPKKK1, MEKK, MEKK 1, MEKK1, | mitogen-activated protein kinase kinase kinase 1 |
| EGFR | ERBB, ERBB1, HER1, NISBD2, PIG61 | epidermal growth factor receptor |
| ERBB4 | ALS19, HER4, p180erbB4 | erb-b2 receptor tyrosine kinase 4 |
| TNF | DIF-alpha, TNFA, TNFSF2, TNLG1F, | tumor necrosis factor |
| VEGFA | MVCD1, VEGF, VPF | vascular endothelial growth factor A |
| CCAR2 | DBC-1, DBC1, KIAA1967, NET35, p3 | cell cycle and apoptosis regulator 2 |
| IL6 | BSF-2, BSF2, CDF, HGF, HSF, IFN-be | interleukin 6 |
| CDKN2B | CDK4I, INK4B, MTS2, P15, TP15, p1 | cyclin dependent kinase inhibitor 2B |
| TGFB1 | CED, DPD1, IBDIMDE, LAP, TGF-bet | transforming growth factor beta 1 |
| MTA3 | | metastasis associated 1 family member 3 |
| TCF7L2 | TCF-4, TCF4 | transcription factor 7 like 2 |
| ERBB2 | CD340, HER-2, HER-2/neu, HER2, N | erb-b2 receptor tyrosine kinase 2 |
| TGFBR2 | AAT3, FAA3, LDS1B, LDS2, LDS2B, N | transforming growth factor beta receptor 2 |
| CDKN2B-A | ANRIL, CDKN2B-AS, CDKN2BAS, NC | CDKN2B antisense RNA 1 |
| HIF1A | HIF-1-alpha, HIF-1A, HIF-1alpha, HI | hypoxia inducible factor 1 subunit alpha |
| AREG | ARB, CRDGF, SDGF, AREG | amphiregulin |
| MMP9 | CLG4B, GELB, MANDP2, MMP-9 | matrix metallopeptidase 9 |

| | | |
|---|---|---|
| AR | AIS8, DHTR, HUMARA, HYSP1, KD, | androgen receptor |
| AKT1 | AKT, CWS6, PKB, PKB-ALPHA, PRKB | AKT serine/threonine kinase 1 |
| NFKB1 | CVID12, EBP-1, KBF1, NF-kB, NF-kB | nuclear factor kappa B subunit 1 |
| KLF4 | EZF, GKLF | Kruppel like factor 4 |
| STAT3 | ADMIO, ADMIO1, APRF, HIES | signal transducer and activator of transcription 3 |
| KRAS | 'C-K-RAS, C-K-RAS, CFC2, K-RAS2A, | KRAS proto-oncogene, GTPase |
| CERS2 | L3, LASS2, SP260, TMSG1 | ceramide synthase 2 |
| IL1B | IL-1, IL1-BETA, IL1F2, IL1beta | interleukin 1 beta |
| PTGS2 | COX-2, COX2, GRIPGHS, PGG/HS, P | prostaglandin-endoperoxide synthase 2 |
| IL10 | CSIF, GVHDS, IL-10A, TGIF, IL10 | interleukin 10 |
| CRP | PTX1 | C-reactive protein |
| TLR4 | ARMD10, CD284, TLR-4, TOLL | toll like receptor 4 |
| CTNNB1 | CTNNB, EVR7, MRD19, NEDSDV, ar | catenin beta 1 |
| PTEN | 10q23del, BZS, CWS1, DEC, GLM2, | phosphatase and tensin homolog |
| CXCL8 | GCP-1, GCP1, IL8, LECT, LUCT, LYN | C-X-C motif chemokine ligand 8 |
| NUPR1 | COM1, P8 | nuclear protein 1, transcriptional regulator |
| CD44 | CDW44, CSPG8, ECMR-III, HCELL, H | CD44 molecule (Indian blood group) |
| CDH1 | Arc-1, BCDS1, CD324, CDHE, ECAD, | cadherin 1 |
| MTOR | FRAP, FRAP1, FRAP2, RAFT1, RAPT | mechanistic target of rapamycin kinase |
| BCL2 | Bcl-2, PPP1R50 | BCL2 apoptosis regulator |
| SERPINB5 | PI5, maspin | serpin family B member 5 |
| CXCR4 | CD184, D2S201E, FB22, HM89, HSY | C-X-C motif chemokine receptor 4 |
| MED19 | DT2P1G7, LCMR1AS, MED19 | mediator complex subunit 19 |
| S100A14 | BCMP84, S100A15 | S100 calcium binding protein A14 |
| MDM2 | ACTFS, HDMX, LSKB, hdm2 | MDM2 proto-oncogene |
| MMP2 | CLG4, CLG4A, MMP-2, MMP-II, MC | matrix metallopeptidase 2 |
| ACE | ACE1, CD143, DCP, DCP1 | angiotensin I converting enzyme |
| MAPK1 | ERK, ERK-2, ERK2, ERT1, MAPK2, P4 | mitogen-activated protein kinase 1 |
| CD274 | B7-H, B7H1, PD-L1, PDCD1L1, PDCL | CD274 molecule |
| SERPINE1 | PAI, PAI-1, PAI1, PLANH1 | serpin family E member 1 |
| FHIT | AP3Aase, FRA3B | fragile histidine triad diadenosine triphosphatase |
| MTHFR | | methylenetetrahydrofolate reductase |
| CDKN1A | CAP20, CDKN1, CIP1, MDA-6, P21, | cyclin dependent kinase inhibitor 1A |
| CCL2 | GDCF-2, HC11, HSMCR30, MCAF, M | C-C motif chemokine ligand 2 |
| BIRC5 | API4, EPR-1 | baculoviral IAP repeat containing 5 |
| PIK3CA | CLAPO, CLOVE, CWS5, MCAP, MCM | phosphatidylinositol-4,5-bisphosphate 3-kinase catalytic subunit |
| BDNF | ANON2, BULN2 | brain derived neurotrophic factor |
| NOTCH1 | AOS5, AOVD1, TAN1, hN1 | notch receptor 1 |
| MIR21 | MIRN21, hsa-mir-21, miR-21, miRN | microRNA 21 |
| MET | AUTS9, DFNB97, HGFR, RCCP2, c-M | MET proto-oncogene, receptor tyrosine kinase |
| CXCL12 | IRH, PBSF, SCYB12, SDF1, TLSF, TPA | C-X-C motif chemokine ligand 12 |
| LEP | LEPD, OB, OBS | leptin |
| SIRT1 | SIR2, SIR2L1, SIR2alpha | sirtuin 1 |
| HMGB1 | HMG-1, HMG1, HMG3, SBP-1 | high mobility group box 1 |
| BCAR4 | | breast cancer anti-estrogen resistance 4 |
| FAS | ALPS1A, APO-1, APT1, CD951, FAST | Fas cell surface death receptor |
| ICAM1 | BB2, CD54, P3.58 | intercellular adhesion molecule 1 |
| RELA | CMCU, NFKB3, p65 | RELA proto-oncogene, NF-kB subunit |
| ESR2 | ER-BETA, ESR-BETA, ESRB, ESTRB, E | estrogen receptor 2 |
| SPP1 | BNSP, BSPI, ETA-1, OPN | secreted phosphoprotein 1 |
| FOXP3 | AIID, DIETER, IPEX, JM2, PIDX, XPID | forkhead box P3 |

| | | |
|---|---|---|
| ABCB1 | ABC20, CD243, CLCS, GP170, MDR1 | ATP binding cassette subfamily B member 1 |
| GSK3B | | glycogen synthase kinase 3 beta |
| EZH2 | ENX-1, ENX1b, KMT6, KMT6A, WVS | enhancer of zeste 2 polycomb repressive complex 2 subunit |
| BRAF | B-RAF1, B-raf1, NS7, RAFB1, BRAF | B-Raf proto-oncogene, serine/threonine kinase |
| IGF1R | CD221, IGFIR, IGFR, JTK13 | insulin like growth factor 1 receptor |
| SRC | ASV1, THC6, c-SRC, p60-Src, SRC | SRC proto-oncogene, non-receptor tyrosine kinase |
| ITGB3 | BDPLT16, BDPLT2, CD61, GP3A, GP | integrin subunit beta 3 |
| ITGB1 | CD29, FNRB, GPIIA, MDF2, MSK12, | integrin subunit beta 1 |
| PPARG | CIMT1, GLM1, NR1C31, PPARG2, P | peroxisome proliferator activated receptor gamma |
| CAV1 | BSCL3, CGL3, LCCNS, MSTP085, PP | caveolin 1 |
| RB1 | OSRC, PPP1R130, RB, p105-Rb, p11 | RB transcriptional corepressor 1 |
| NOS3 | ECNOS, eNOS | nitric oxide synthase 3 |
| MUC1 | ADMCKD, ADMCKD1, CA 15-3, CD2 | mucin 1, cell surface associated |
| IFNG | IFG, IFI | interferon gamma |
| MAPK3 | ERK-1, ERK1, ERT2, HS44KDAP, HU | mitogen-activated protein kinase 3 |
| HMOX1 | HMOX1D, HO-1, HSP32, bK286B10 | heme oxygenase 1 |
| CDKN1B | CDKN4, KIP1, MEN1B, MEN4, P27K | cyclin dependent kinase inhibitor 1B |
| MMP1 | CLG, CLGN | matrix metallopeptidase 1 |
| SP1 | | Sp1 transcription factor |
| RAC1 | MIG5, MRD48, Rac-1, TC-25, p21-R | Rac family small GTPase 1 |
| VDR | NR1I1, PPP1R163 | vitamin D receptor |
| JUN | AP-1, AP1, c-Jun, cJUN, p39 | Jun proto-oncogene, AP-1 transcription factor subunit |
| LGALS3 | CBP35, GAL3, GALBP, GALIG, L31, L | galectin 3 |
| KDR | CD309, FLK1, VEGFR, VEGFR2 | kinase insert domain receptor |
| TP63 | AIS, B(p51A), B(p51B), EEC3, KET, L | tumor protein p63 |
| LCN2 | 24p3, MSFI, NGAL, p25 | lipocalin 2 |
| ADIPOQ | ACDC, ACRP30, ADIPQTL1, ADPN, A | adiponectin, C1Q and collagen domain containing |
| MAPK14 | CSBP, CSBP1, CSBP2, CSPB1, EXIP, I | mitogen-activated protein kinase 14 |
| SOD2 | GClnc1, IPO-B, IPOB, MNSOD, MVC | superoxide dismutase 2 |
| KIT | C-Kit, CD117, MASTC, PBT, SCFR | KIT proto-oncogene, receptor tyrosine kinase |
| PTK2 | FADK, FAK, FAK1, FRNK, PPP1R71, | protein tyrosine kinase 2 |
| NFE2L2 | HEBP1, IMDDHH, NRF2, Nrf-2 | nuclear factor, erythroid 2 like 2 |
| RHOA | ARH12, ARHA, EDFAOB, RHO12, RH | ras homolog family member A |
| HGF | DFNB39, F-TCFB, HPTA, SF, HGF | hepatocyte growth factor |
| TNFRSF11 | OCIF, OPG, PDB5, TR1 | TNF receptor superfamily member 11b |
| MKI67 | KIA, MIB-, MIB-1, PPP1R105 | marker of proliferation Ki-67 |
| JAK2 | JTK10, THCYT3 | Janus kinase 2 |
| IL18 | IGIF, IL-18, IL-1g, IL1F4 | interleukin 18 |
| ATM | AT1, ATA, ATC, ATD, ATDC, ATE, TE | ATM serine/threonine kinase |
| YAP1 | COB1, YAP, YAP2, YAP65, YKI | Yes1 associated transcriptional regulator |
| MAGED2 | 11B6, BARTS5, BCG-1, BCG1, HCA1 | MAGE family member D2 |
| FN1 | CIG, ED-B, FINC, FN, FNZ, GFND, GF | fibronectin 1 |
| APC | BTPS2, DESMD, DP2, DP2.5, DP3, G | APC regulator of WNT signaling pathway |
| CCR5 | CC-CKR-5, CCCKR5, CCR-5, CD195, C | C-C motif chemokine receptor 5 (gene/pseudogene) |
| F3 | CD142, TF, TFA | coagulation factor III, tissue factor |
| MMP3 | CHDS6, MMP-3, SL-1, STMY, STMY | matrix metallopeptidase 3 |
| XRCC1 | RCC, SCAR26 | X-ray repair cross complementing 1 |
| GJA1 | AVSD3, CMDR, CX43, EKVP, EKVP3, | gap junction protein alpha 1 |
| LDOC1 | BCUR1, Mar7, Mart7, RTL7, SIRH7 | LDOC1 regulator of NFKB signaling |
| AURKA | AIK, ARK1, AURA, BTAK, PPP1R47, | aurora kinase A |
| CCL5 | D17S136E, RANTES, SCYA5, SIS-del | C-C motif chemokine ligand 5 |

| | | |
|---|---|---|
| GSTM1 | GST1-1, GSTM1a-1a, GSTM1b-1b, G | glutathione S-transferase mu 1 |
| MIF | GIF, GLIF, MMIF | macrophage migration inhibitory factor |
| IL17A | CTLA-8, CTLA8, IL-17, IL-17A, IL17 | interleukin 17A |
| HSPA5 | BIP, GRP78, HEL-S-89n, MIF2 | heat shock protein family A (Hsp70) member 5 |
| TNFSF11 | CD254, ODF, OPGL, OPTB2, RANKL | TNF superfamily member 11 |
| SMAD4 | DPC4, JIP, MADH4, MYHRS | SMAD family member 4 |
| ARID4B | BCAA, BRCAA1, RBBP1L1, RBP1L1, | AT-rich interaction domain 4B |
| FLT1 | FLT, FLT-1, VEGFR-1, VEGFR1 | fms related receptor tyrosine kinase 1 |
| BSG | 5F7, CD147, EMMPRIN, EMPRIN, O | basigin (Ok blood group) |
| POU5F1 | OCT3, OCT4, OTF-3, OTF3, OTF4, O | POU class 5 homeobox 1 |
| MIR155 | MIRN155, miRNA155, mir-155 | microRNA 155 |
| HLA-G | MHC-G | major histocompatibility complex, class I, G |
| SMAD3 | HSPC193, HsT17436, JV15-2, LDS1 | SMAD family member 3 |
| TWIST1 | ACS3, BPES2, BPES3, CRS, CRS1, CS | twist family bHLH transcription factor 1 |
| PLAUR | CD87, U-PAR, UPAR, URKR | plasminogen activator, urokinase receptor |
| TIMP1 | CLGI, EPA, EPO, HCI, TIMP, TIMP-1 | TIMP metallopeptidase inhibitor 1 |
| IGFBP3 | BP-53, IBP3 | insulin like growth factor binding protein 3 |
| FGFR1 | BFGFR, CD331, CEK, ECCL, FGFBR, | fibroblast growth factor receptor 1 |
| MIR146A | MIRN146, MIRN146A, miR-146a, m | microRNA 146a |
| SOX2 | ANOP3, MCOPS3 | SRY-box transcription factor 2 |
| SLC2A1 | CSE, DYT17, DYT18, DYT9, EIG12, G | solute carrier family 2 member 1 |
| MIR34A | MIRN34A, miRNA34A, mir-34, mir- | microRNA 34a |
| AGER | RAGE, SCARJ1 | advanced glycosylation end-product specific receptor |
| FOXO3 | AF6q21, FKHRL1, FKHRL1P2, FOXO | forkhead box O3 |
| EGF | HOMG4, URG | epidermal growth factor |
| FGF2 | BFGF, FGF-2, FGFB, HBGF-2 | fibroblast growth factor 2 |
| CHI3L1 | ASRT7, CGP-39, GP-39, GP39, HC-g | chitinase 3 like 1 |
| BMI1 | FLVI2/BMI1, PCGF4, RNF51, flvi-2/ | BMI1 proto-oncogene, polycomb ring finger |
| SYTL2 | CHR11SYT, EXO4, PPP1R151, SGA7 | synaptotagmin like 2 |
| MMP14 | MMP-14, MMP-X1, MT-MMP, MT- | matrix metallopeptidase 14 |
| E2F1 | E2F-1, RBAP1, RBBP3, RBP3 | E2F transcription factor 1 |
| PLAU | ATF, BDPLT5, QPD, UPA, URK, u-PA | plasminogen activator, urokinase |
| VIM | | vimentin |
| CASP8 | ALPS2B, CAP4, Casp-8, FLICE, MAC | caspase 8 |
| WT1 | AWT1, GUD, NPHS4, WAGR, WIT-2 | WT1 transcription factor |
| FOXM1 | FKHL16A, FOXM1B, FOXM1C, HFH- | forkhead box M1 |
| CTLA4 | ALPS5, CD, CD152, CELIAC3, CTLA-4 | cytotoxic T-lymphocyte associated protein 4 |
| CDC42 | CDC42Hs, G25K, TKS | cell division cycle 42 |
| ITGAV | CD51, MSK8, VNRA, VTNR | integrin subunit alpha V |
| MIR145 | MIRN145, miR-145, miRNA145 | microRNA 145 |
| CASP3 | CPP32, CPP32B, SCA-1 | caspase 3 |
| STAT1 | CANDF7, IMD31A, IMD31B, IMD31 | signal transducer and activator of transcription 1 |
| EPAS1 | ECYT4, HIF2A, HLF, MOP2, PASD2, | endothelial PAS domain protein 1 |
| EZR | CVIL, CVL, HEL-S-105, VIL2 | ezrin |
| SNAI1 | SLUGH2, SNA, SNAH, SNAIL, SNAIL1 | snail family transcriptional repressor 1 |
| GSTT1 | | glutathione S-transferase theta 1 |
| PGR | NR3C3, PR | progesterone receptor |
| RUNX2 | AML3, CBF-alpha-1, CBFA1, CCD, C | RUNX family transcription factor 2 |
| ANXA2 | ANX2, ANX2L4, CAL1H, HEL-S-270, | annexin A2 |
| GSTP1 | DFN7, FAEES3, GST3, GSTP, HEL-S-2 | glutathione S-transferase pi 1 |
| VEGFC | Flt4-L, LMPH1D, LMPHM4, VRP | vascular endothelial growth factor C |

| | | |
|---|---|---|
| PRKCA | AAG6, PKC-alpha, PKCA, PKCI+/-, P | protein kinase C alpha |
| DICER1 | DCR1, Dicer, Dicer1e, GLOW, HERN | dicer 1, ribonuclease III |
| PDCD1 | CD279, PD-1, PD1, SLEB2, hPD-1, h | programmed cell death 1 |
| PARP1 | ADPRT, ADPRT 1, ADPRT1, ARTD1, | poly(ADP-ribose) polymerase 1 |
| COL1A1 | CAFYD, EDSARTH1, EDSC, OI1, OI2, | collagen type I alpha 1 chain |
| ZEB1 | AREB6, BZP, DELTAEF1, FECD6, NIL | zinc finger E-box binding homeobox 1 |
| LYPD5 | PRO4356 | LY6/PLAUR domain containing 5 |
| BECN1 | ATG6, VPS30, beclin1 | beclin 1 |
| STK11 | LKB1, PJS, hLKB1 | serine/threonine kinase 11 |
| SELE | CD62E, ELAM, ELAM1, ESEL, LECAM | selectin E |
| DKK1 | DKK-1, SK | dickkopf WNT signaling pathway inhibitor 1 |
| SYK | p72-Syk | spleen associated tyrosine kinase |
| S100A9 | 60B8AG, CAGB, CFAG, CGLB, L1AG, | S100 calcium binding protein A9 |
| CCN2 | CTGF, HCS24, IGFBP8, NOV2 | cellular communication network factor 2 |
| HMGA2 | BABL, HMGI-C, HMGIC, LIPO, STQT | high mobility group AT-hook 2 |
| RUNX3 | AML2, CBFA3, PEBP2aC | RUNX family transcription factor 3 |
| SOX9 | CMD1, CMPD1, SRA1, SRXX2, SRXY | SRY-box transcription factor 9 |
| PAK1 | IDDMSSD, PAKalpha, alpha-PAK, p6 | p21 (RAC1) activated kinase 1 |
| RASSF1 | 123F2, NORE2AA, RDA32, REH3P21 | Ras association domain family member 1 |
| TET2 | KIAA1546, MDS | tet methylcytosine dioxygenase 2 |
| HPSE | HPA, HPA1, HPR11, HSE1, HPSE | heparanase |
| HOTAIR | HOXAS, HOXC-AS4, HOXC11-AS1, N | HOX transcript antisense RNA |
| WNT5A | hWNT5A | Wnt family member 5A |
| PKM | CTHBP, HEL-S-30, OIP3, PK32, TCB, | pyruvate kinase M1/2 |
| ALDH1A1 | ALDC, ALDH-E1, ALDH1, ALDH11, H | aldehyde dehydrogenase 1 family member A1 |
| YBX1 | BP-8, CBF-A, CSDA2, CSDB, DBPB, E | Y-box binding protein 1 |
| H19 | ASM, ASM1, BWS, D11S813E, LINC | H19 imprinted maternally expressed transcript |
| TGFBR1 | AAT5, ACVRLK4, ALK-5, ALK5, ESS1 | transforming growth factor beta receptor 1 |
| AGR3 | AG-3, AG3, BCMP11, HAG3, PDIA1 | anterior gradient 3, protein disulphide isomerase family membe |
| EPCAM | DIAR5, EGP-2, EGP314, EGP40, ESA | epithelial cell adhesion molecule |
| EIF4E | AUTS19, CBP1, EIF4EL1, EIF4F, eIF- | eukaryotic translation initiation factor 4E |
| SHH | HHG1, HLP3, HPE3, MCOPCB5, SM | sonic hedgehog signaling molecule |
| CFL1 | CFL, HEL-S-15, cofilin | cofilin 1 |
| CA9 | CAIX, MN | carbonic anhydrase 9 |
| MAPK8 | JNK, JNK-46, JNK1, JNK1A2, JNK21E | mitogen-activated protein kinase 8 |
| XIAP | API3, BIRC4, IAP-3, ILP1, MIHA, XLP | X-linked inhibitor of apoptosis |
| HSPB1 | CMT2F, HEL-S-102, HMN2B, HS.76 | heat shock protein family B (small) member 1 |
| MGMT | | O-6-methylguanine-DNA methyltransferase |
| PROM1 | AC133, CD133, CORD12, MCDR2, N | prominin 1 |
| PLK1 | PLK, STPK13 | polo like kinase 1 |
| GLI1 | GLI, PAPA8, PPD1 | GLI family zinc finger 1 |
| S100A4 | 18A2, 42A, CAPL, FSP1, MTS1, P9K | S100 calcium binding protein A4 |
| TLR9 | CD289 | toll like receptor 9 |
| ELAVL1 | ELAV1, HUR, Hua, MelG | ELAV like RNA binding protein 1 |
| TGM2 | TG(C), TGC | transglutaminase 2 |
| AGTR1 | AG2SB, AT1, AT1AR, AT1B, AT1BR, | angiotensin II receptor type 1 |
| RET | CDHF12, CDHR16, HSCR1, MEN2A, | ret proto-oncogene |
| TNFSF10 | APO2L, Apo-2L, CD253, TL2, TNLG | TNF superfamily member 10 |
| NOS2 | HEP-NOS, INOS, NOSA, NOS2 | nitric oxide synthase 2 |
| SOCS3 | ATOD4, CIS3, Cish3, SOCS-3, SSI-3, | suppressor of cytokine signaling 3 |
| ALK | CD246, NBLST3 | ALK receptor tyrosine kinase |

| | | |
|---|---|---|
| CREB1 | CREB, CREB-1 | cAMP responsive element binding protein 1 |
| F2R | CF2R, HTR, PAR-1, PAR1, TR | coagulation factor II thrombin receptor |
| NAMPT | 1110035O14Rik, PBEF, PBEF1, VF, V | nicotinamide phosphoribosyltransferase |
| AHR | RP85, bHLHe76 | aryl hydrocarbon receptor |
| BAX | BCL2L4 | BCL2 associated X, apoptosis regulator |
| ERCC1 | COFS4, RAD10, UV20 | ERCC excision repair 1, endonuclease non-catalytic subunit |
| HDAC1 | GON-10, HD1, KDAC1, RPD3, RPD3 | histone deacetylase 1 |
| CYP1A1 | AHH, AHRR, CP11, CYP1, CYPIA1, P | cytochrome P450 family 1 subfamily A member 1 |
| TYMS | HST422, TMS, TS | thymidylate synthetase |
| IRS1 | HIRS-1 | insulin receptor substrate 1 |
| ITGA5 | CD49e, FNRA, VLA-5, VLA5A | integrin subunit alpha 5 |
| SMAD2 | JV18, JV18-1, MADH2, MADR2, hM | SMAD family member 2 |
| TIMP2 | CSC-21K, DDC8 | TIMP metallopeptidase inhibitor 2 |
| ANXA1 | ANX1, LPC1 | annexin A1 |
| GATA3 | HDR, HDRS | GATA binding protein 3 |
| CDH13 | CDHH, P105 | cadherin 13 |
| TP73 | P73 | tumor protein p73 |
| ADRB2 | ADRB2R, ADRBR, B2AR, BAR, BETA | adrenoceptor beta 2 |
| FASLG | ALPS1B, APT1LG1, APTL, CD178, CI | Fas ligand |
| APEX1 | APE, APE1, APEN, APEX, APX, HAP1 | apurinic/apyrimidinic endodeoxyribonuclease 1 |
| CD24 | CD24A | CD24 molecule |
| MMP13 | CLG3, MANDP1, MDST, MMP-13 | matrix metallopeptidase 13 |
| PARK7 | DJ-1, DJ1, GATD2, HEL-S-67p | Parkinsonism associated deglycase |
| AXL | ARK, JTK11, Tyro7, UFO | AXL receptor tyrosine kinase |
| MIR221 | MIRN221, miRNA221, mir-221 | microRNA 221 |
| F2RL1 | GPR11, PAR2 | F2R like trypsin receptor 1 |
| FBXW7 | AGO, CDC4, FBW6, FBW7, FBX30, F | F-box and WD repeat domain containing 7 |
| MLH1 | COCA2, FCC2, HNPCC, HNPCC2, hM | mutL homolog 1 |
| ROCK1 | P160ROCK, ROCK-I | Rho associated coiled-coil containing protein kinase 1 |
| CYP3A4 | CP33, CP34, CYP3A, CYP3A3, CYPIII | cytochrome P450 family 3 subfamily A member 4 |
| RETN | ADSF, FIZZ31, RSTN, XCP1, RETN | resistin |
| MYB | Cmyb, c-myb, c-myb_CDS, efg | MYB proto-oncogene, transcription factor |
| SPARC | BM-40, OI17, ON, ONT | secreted protein acidic and cysteine rich |
| GLI2 | CJS, HPE9, PHS2, THP1, THP2 | GLI family zinc finger 2 |
| CYP19A1 | ARO, ARO1, CPV1, CYAR, CYP19, CY | cytochrome P450 family 19 subfamily A member 1 |
| SNAI2 | SLUG, SLUGH, SLUGH1, SNAIL2, WS | snail family transcriptional repressor 2 |
| IDO1 | IDO, IDO-1, INDO | indoleamine 2,3-dioxygenase 1 |
| ID1 | ID, bHLHb24 | inhibitor of DNA binding 1, HLH protein |
| MIR126 | MIRN126, miRNA126, mir-126 | microRNA 126 |
| SPHK1 | SPHK | sphingosine kinase 1 |
| CCR7 | BLR2, CC-CKR-7, CCR-7, CD197, CD | C-C motif chemokine receptor 7 |
| CEACAM5 | CD66e, CEA | CEA cell adhesion molecule 5 |
| POSTN | OSF-2, OSF2, PDLPOSTN, PN | periostin |
| ETS1 | ETS-1, EWSR2, c-ets-1, p54 | ETS proto-oncogene 1, transcription factor |
| MIR29A | MIRN29, MIRN29A, hsa-mir-29, hs | microRNA 29a |
| KRT19 | CK19, K19, K1CS | keratin 19 |
| ERBB3 | ErbB-3, FERLK, HER3, LCCS2, MDA- | erb-b2 receptor tyrosine kinase 3 |
| RAD51 | BRCC5, FANCR, HRAD51, HsRad51, | RAD51 recombinase |
| CTSD | CLN10, CPSD, HEL-S-130P | cathepsin D |
| TLR2 | CD282, TIL4 | toll like receptor 2 |
| MIR200C | MIRN200C, mir-200c | microRNA 200c |

| | | |
|---|---|---|
| KIAA0100 | BCOX, BCOX1, CT101, FMP27 | KIAA0100 |
| LMNA | CDCD1, CDDC, CMD1A, CMT2B1, E | lamin A/C |
| LEPR | CD295, LEP-RD, OB-R, OBR, LEPR | leptin receptor |
| PPARGC1A | LEM6, PGC-1(alpha), PGC-1alpha, F | PPARG coactivator 1 alpha |
| IGF2 | C11orf43, GRDF, IGF-II, PP9974 | insulin like growth factor 2 |
| STMN1 | C1orf215, LAP18, Lag, OP18, PP17, | stathmin 1 |
| NDRG1 | CAP43, CMT4D, DRG-1, DRG1, GC4 | N-myc downstream regulated 1 |
| DNMT1 | ADCADN, AIM, CXXC9, DNMT, HSN | DNA methyltransferase 1 |
| JAG1 | AGS, AGS1, AHD, AWS, CD339, DCH | jagged canonical Notch ligand 1 |
| ENG | END, HHT1, ORW1 | endoglin |
| EPHA2 | ARCC2, CTPA, CTPP1, CTRCT6, ECK | EPH receptor A2 |
| BMP2 | BDA2A, SSFSC, BMP2 | bone morphogenetic protein 2 |
| MCL1 | BCL2L3, EAT-ES, MCL1L, MCL1S, M | MCL1 apoptosis regulator, BCL2 family member |
| ADAM17 | ADAM18, CD156B, CSVP, NISBD, N | ADAM metallopeptidase domain 17 |
| MIR143 | MIRN143, mir-143 | microRNA 143 |
| CCN1 | CYR61, GIG1, IGFBP10 | cellular communication network factor 1 |
| GPAT3 | AGPAT 10, AGPAT10, AGPAT8, AGF | glycerol-3-phosphate acyltransferase 3 |
| NRP1 | BDCA4, CD304, NP1, NRP, VEGF165 | neuropilin 1 |
| MSH2 | COCA1, FCC1, HNPCC, HNPCC1, LCI | mutS homolog 2 |
| GHRL | MTLRP | ghrelin and obestatin prepropeptide |
| PTPN11 | BPTP3, CFC, JMML, METCDS, NS1, | protein tyrosine phosphatase non-receptor type 11 |
| GJB2 | BAPS, CX26, DFNA3, DFNA3A, DFN | gap junction protein beta 2 |
| ITGA6 | CD49fB, VLA-6, ITGA6 | integrin subunit alpha 6 |
| MIR27A | MIR27, MIRN27A, mir-27a | microRNA 27a |
| WWTR1 | TAZ | WW domain containing transcription regulator 1 |
| IL1A | IL-1 alpha, IL-1A, IL1, IL1-ALPHA, IL | interleukin 1 alpha |
| PEBP1 | HCNP, HCNPpp, HEL-210, HEL-S-34 | phosphatidylethanolamine binding protein 1 |
| CXCL1 | FSP, GRO1, GROa, MGSA, MGSA-a, | C-X-C motif chemokine ligand 1 |
| LOX | AAT10 | lysyl oxidase |
| SDC1 | CD138, SDC, SYND1, syndecan | syndecan 1 |
| TNFRSF11 | CD265, FEO, LOH18CR1, ODFR, OFI | TNF receptor superfamily member 11a |
| AKT2 | HIHGHH, PKBB, PKBBETA, PRKBB, F | AKT serine/threonine kinase 2 |
| KDM1A | AOF2, BHC110, CPRF, KDM1, LSD1 | lysine demethylase 1A |
| B2M | IMD43 | beta-2-microglobulin |
| HSP90AA1 | EL52, HEL-S-65p, HSP86, HSP89A, H | heat shock protein 90 alpha family class A member 1 |
| MMP7 | MMP-7, MPSL1, PUMP-1 | matrix metallopeptidase 7 |
| ITGB4 | CD104, GP150 | integrin subunit beta 4 |
| IL4 | BCGF-1, BCGF1, BSF-1, BSF1, IL-4 | interleukin 4 |
| OGG1 | HMMH, HOGG1, MUTM, OGH1 | 8-oxoguanine DNA glycosylase |
| TOP2A | TOP2, TP2A | DNA topoisomerase II alpha |
| NTN4 | PRO3091 | netrin 4 |
| CD4 | CD4mut | CD4 molecule |
| SERPINA1 | A1A, A1AT, AAT, PI, PI1, PRO2275, | serpin family A member 1 |
| CFTR | ABC35, ABCC7, CF/MRP, MRP7, TN | CF transmembrane conductance regulator |
| THBS1 | THBS, THBS-1, TSP, TSP-1, TSP1 | thrombospondin 1 |
| HRAS | C-BAS/HAS, C-H-RAS, C-HA-RAS1, C | HRas proto-oncogene, GTPase |
| FOXO1 | FKH1, FKHRA, FOXO1 | forkhead box O1 |
| NRG1 | ARIA, GGF, GGF2, HGL, HRG, HRG1 | neuregulin 1 |
| NANOG | | Nanog homeobox |
| NBN | AT-V1, AT-V2, ATV, NBS, NBS1, P95 | nibrin |
| PIK3R1 | AGM7, GRB1, IMD36, p85, p85-ALI | phosphoinositide-3-kinase regulatory subunit 1 |

| | | |
|---|---|---|
| F2 | PT, RPRGL2, THPH1 | coagulation factor II, thrombin |
| FGFR3 | ACH, CD333, CEK2, HSFGFR3EX, JTI | fibroblast growth factor receptor 3 |
| PRL | GHA1 | prolactin |
| RUNX1 | AML1, AML1-EVI-1, AMLCR1, CBF2 | RUNX family transcription factor 1 |
| DNMT3B | ICF, ICF1, M.HsaIIIB | DNA methyltransferase 3 beta |
| ADAM10 | AD10, AD18, CD156c, CDw156, HsT | ADAM metallopeptidase domain 10 |
| ACKR3 | CMKOR1, CXC-R7, CXCR-7, CXCR7, | atypical chemokine receptor 3 |
| PRKAA1 | AMPK, AMPKa1 | protein kinase AMP-activated catalytic subunit alpha 1 |
| GRN | CLN11, GEP, GP88, PCDGF, PEPI, PC | granulin precursor |
| BCL2L1 | BCL-XL/S, BCL2L, BCLX, Bcl-X, PPP1 | BCL2 like 1 |
| INSR | CD220, HHF5 | insulin receptor |
| NF1 | NFNS, VRNF, WSS | neurofibromin 1 |
| SOCS1 | CIS1, CISH1, JAB, SOCS-1, SSI-1, SSI | suppressor of cytokine signaling 1 |
| LGALS1 | GAL1, GBP | galectin 1 |
| NES | Nbla00170 | nestin |
| EDN1 | ARCND3, ET1, HDLCQ7, PPET1, QM | endothelin 1 |
| VCAM1 | CD106, INCAM-100 | vascular cell adhesion molecule 1 |
| NTRK2 | EIEE58, GP145-TrkB, OBHD, TRKB, | neurotrophic receptor tyrosine kinase 2 |
| CLDN1 | CLD1, ILVASC, SEMP1 | claudin 1 |
| MIR200B | MIRN200B, mir-200b | microRNA 200b |
| HMGA1 | HMG-RA, HMGIY, HMGA1 | high mobility group AT-hook 1 |
| CYP1B1 | ASGD6, CP1B, CYPIB1, GLC3A, P450 | cytochrome P450 family 1 subfamily B member 1 |
| EGR1 | AT225, G0S30, KROX-24, NGFI-A, T | early growth response 1 |
| ABL1 | ABL, BCR-ABL, CHDSKM, JTK7, bcr/ | ABL proto-oncogene 1, non-receptor tyrosine kinase |
| PIN1 | DOD, UBL5 | peptidylprolyl cis/trans isomerase, NIMA-interacting 1 |
| NQO1 | DHQU, DIA4, DTD, NMOR1, NMOR | NAD(P)H quinone dehydrogenase 1 |
| CDK1 | CDC2, CDC28A, P34CDC2 | cyclin dependent kinase 1 |
| MIRLET7A | LET7A1, MIRNLET7A1, let-7a-1 | microRNA let-7a-1 |
| EP300 | KAT3B, MKHK2, RSTS2, p300 | E1A binding protein p300 |
| IL33 | C9orf26, DVS27, IL1F11, NF-HEV, N | interleukin 33 |
| BAP1 | HUCEP-13, UCHL2, hucep-6 | BRCA1 associated protein 1 |
| ILK | HEL-S-28-1, ILK-2, P59, p59ILK, ILK | integrin linked kinase |
| NEAT1 | LINC00084, NCRNA00084, TncRNA | nuclear paraspeckle assembly transcript 1 |
| SKP2 | FBL1, FBXL1, FLB1, p45 | S-phase kinase associated protein 2 |
| GDF15 | GDF-15, MIC-1, MIC1, NAG-1, PDF, | growth differentiation factor 15 |
| PTTG1 | EAP1, HPTTG, PTTG, TUTR1 | PTTG1 regulator of sister chromatid separation, securin |
| MIR124-1 | MIR124A, MIR124A1, MIRN124-1, | microRNA 124-1 |
| PRKDC | DNA-PKC, DNA-PKcs, DNAPK, DNAI | protein kinase, DNA-activated, catalytic subunit |
| CST3 | ARMD11, HEL-S-2 | cystatin C |
| KCNN4 | DHS2, IK, IK1, IKCA1, KCA4, KCa3.1 | potassium calcium-activated channel subfamily N member 4 |
| ANGPT2 | AGPT2, ANG2 | angiopoietin 2 |
| MCAM | CD146, HEMCAM, METCAM, MUC1 | melanoma cell adhesion molecule |
| PXN | | paxillin |
| MIR30A | MIRN30A, mir-30a | microRNA 30a |
| MIR203A | MIR203, MIRN203, hsa-mir-203a, r | microRNA 203a |
| ATF3 | | activating transcription factor 3 |
| CD36 | BDPLT10, CHDS7, FAT, GP3B, GP4, | CD36 molecule |
| CLU | AAG4, APO-J, APOJ, CLI1, CLU2, KU | clusterin |
| DCLK1 | CL1, CLICK1, DCAMKL1, DCDC3A, D | doublecortin like kinase 1 |
| FASN | FAS, OA-519, SDR27X1 | fatty acid synthase |
| UCA1 | CUDR, LINC00178, NCRNA00178, U | urothelial cancer associated 1 |

| | | |
|---|---|---|
| CD80 | B7, B7-1, B7.1, BB1, CD28LG, CD28 | CD80 molecule |
| MME | CALLA, CD10, CMT2T, NEP, SCA43, | membrane metalloendopeptidase |
| CEBPB | C/EBP-beta, IL6DBP, NF-IL6, TCF5 | CCAAT enhancer binding protein beta |
| PIK3CG | PI3CG, PI3K, PI3Kgamma, PIK3, p11 | phosphatidylinositol-4,5-bisphosphate 3-kinase catalytic subunit |
| CDH2 | CD325, CDHN, CDw325, NCAD | cadherin 2 |
| XRCC3 | CMM6 | X-ray repair cross complementing 3 |
| DEPDC1B | BRCC3, XTP1 | DEP domain containing 1B |
| VWF | F8VWF, VWD | von Willebrand factor |
| SMARCA2 | BAF190, BRM, NCBRS, SNF2, SNF2L | SWI/SNF related, matrix associated, actin dependent regulator c |
| PCNA | ATLD2 | proliferating cell nuclear antigen |
| GPER1 | CEPR, CMKRL2, DRY12, FEG-1, GPC | G protein-coupled estrogen receptor 1 |
| MIR182 | MIRN182, miRNA182, mir-182 | microRNA 182 |
| PTP4A3 | PRL-3, PRL-R, PRL3 | protein tyrosine phosphatase 4A3 |
| FGF19 | | fibroblast growth factor 19 |
| PRKN | AR-JP, LPRS2, PARK2, PDJ | parkin RBR E3 ubiquitin protein ligase |
| PDGFRA | CD140A, PDGFR-2, PDGFR2 | platelet derived growth factor receptor alpha |
| ADAM12 | ADAM12-OT1, CAR10, MCMP, MCI | ADAM metallopeptidase domain 12 |
| MIR206 | MIRN206, miRNA206, mir-206 | microRNA 206 |
| BMP4 | BMP2B, BMP2B1, MCOPS6, OFC11 | bone morphogenetic protein 4 |
| SQSTM1 | A170, DMRV, FTDALS3, NADGP, OS | sequestosome 1 |
| ARRDC3 | TLIMP | arrestin domain containing 3 |
| IGFBP2 | IBP2, IGF-BP53 | insulin like growth factor binding protein 2 |
| RPS6KB1 | PS6K, S6K, S6K-beta-1, S6K1, STK14 | ribosomal protein S6 kinase B1 |
| BCAR1 | CAS, CAS1, CASS1, CRKAS, P130Cas | BCAR1 scaffold protein, Cas family member |
| MIR141 | MIRN141, mir-141 | microRNA 141 |
| CHEK1 | CHK1 | checkpoint kinase 1 |
| LDHA | GSD11, HEL-S-133P, LDHM, PIG19 | lactate dehydrogenase A |
| S100A8 | 60B8AG, CAGA, CFAG, CGLA, CP-10 | S100 calcium binding protein A8 |
| SATB1 | | SATB homeobox 1 |
| CXCL10 | C7, IFI10, INP10, IP-10, SCYB10, crg | C-X-C motif chemokine ligand 10 |
| MIR200A | MIRN200A, mir-200a | microRNA 200a |
| NTRK1 | MTC, TRK, TRK1, TRKA, Trk-A, p140 | neurotrophic receptor tyrosine kinase 1 |
| CRYAB | CMD1II, CRYA2, CTPP2, CTRCT16, H | crystallin alpha B |
| AGT | ANHU, SERPINA8, hFLT1 | angiotensinogen |
| BCL2L11 | BAM, BIM, BOD | BCL2 like 11 |
| PPP1R26 | KIAA0649, NRBE3 | protein phosphatase 1 regulatory subunit 26 |
| FOXA1 | HNF3A, TCF3A | forkhead box A1 |
| TIMP3 | HSMRK222, K222, K222TA2, SFD | TIMP metallopeptidase inhibitor 3 |
| NTN1 | MRMV4L, NTN1 | netrin 1 |
| WNT1 | BMND16, INT1, OI15 | Wnt family member 1 |
| ATR | FCTCS, FRP1, MEC1, SCKL, SCKL1 | ATR serine/threonine kinase |
| RHOC | ARH9, ARHC, H9, RHOH9 | ras homolog family member C |
| FGFR4 | CD334, JTK2, TKF | fibroblast growth factor receptor 4 |
| MIR10B | MIRN10B, hsa-mir-10b, miRNA10B | microRNA 10b |
| MIR214 | MIRN214, miRNA214, mir-214 | microRNA 214 |
| WWOX | D16S432E, EIEE28, FOR, FRA16D, H | WW domain containing oxidoreductase |
| L1CAM | CAML1, CD171, HSAS, HSAS1, MAS | L1 cell adhesion molecule |
| CDK4 | CMM3, PSK-J3 | cyclin dependent kinase 4 |
| MIR17 | MIR17-5p, MIR91, MIRN17, MIRN9 | microRNA 17 |
| SOX4 | CSS10, EVI16 | SRY-box transcription factor 4 |
| MIR205 | MIRN205, mir-205 | microRNA 205 |

| | | |
|---|---|---|
| YY1 | DELTA, GADEVS, INO80S, NF-E1, U( | YY1 transcription factor |
| MIR204 | MIRN204, RDICC, miRNA204, mir-2 | microRNA 204 |
| ZEB2 | HSPC082, SIP-1, SIP1, SMADIP1, ZF | zinc finger E-box binding homeobox 2 |
| ALCAM | CD166, MEMD | activated leukocyte cell adhesion molecule |
| INHBB | | inhibin subunit beta B |
| MIR22 | MIRN22, hsa-mir-22, miR-22 | microRNA 22 |
| CDK2 | CDKN2, p33(CDK2) | cyclin dependent kinase 2 |
| CXCR2 | CD182, CDw128b, CMKAR2, IL8R2, | C-X-C motif chemokine receptor 2 |
| AGR2 | AG-2, AG2, GOB-4, HAG-2, HEL-S-1 | anterior gradient 2, protein disulphide isomerase family membe |
| MEG3 | FP504, GTL2, LINC00023, NCRNA00 | maternally expressed 3 |
| MICA | MIC-A, PERB11.1 | MHC class I polypeptide-related sequence A |
| KRT18 | CK-18, CYK18, K18 | keratin 18 |
| HSF1 | HSTF1 | heat shock transcription factor 1 |
| MYD88 | MYD88D | MYD88 innate immune signal transduction adaptor |
| MST1R | CD136, CDw136, NPCA3, PTK8, RO | macrophage stimulating 1 receptor |
| APOA1 | HPALP2, apo(a) | apolipoprotein A1 |
| HBB | CD113t-C, ECYT6, beta-globin | hemoglobin subunit beta |
| TNC | 150-225, DFNA56, GMEM, GP, HXB | tenascin C |
| IGFBP5 | IBP5 | insulin like growth factor binding protein 5 |
| CEACAM1 | BGP, BGP1, BGPI | CEA cell adhesion molecule 1 |
| CCNB1 | CCNB | cyclin B1 |
| PDPN | AGGRUS, GP36, GP40, Gp38, HT1A | podoplanin |
| CTNND1 | BCDS2, CAS, CTNND, P120CAS, P12 | catenin delta 1 |
| CEBPA | C/EBP-alpha, CEBP | CCAAT enhancer binding protein alpha |
| CTTN | EMS1 | cortactin |
| SLIT2 | SLIL3, Slit-2 | slit guidance ligand 2 |
| SLC9A1 | APNH, LIKNS, NHE-1, NHE1, PPP1R | solute carrier family 9 member A1 |
| MIR148A | MIRN148, MIRN148A, hsa-mir-148 | microRNA 148a |
| CCNE1 | CCNE, pCCNE1 | cyclin E1 |
| NOD2 | ACUG, BLAU, BLAUS, CARD15, CD, | nucleotide binding oligomerization domain containing 2 |
| TNFRSF10 | CD262, DR5, KILLER, KILLER/DR5, T | TNF receptor superfamily member 10b |
| OPRM1 | LMOR, M-OR-1, MOP, MOR, MOR1 | opioid receptor mu 1 |
| IGF2BP3 | CT98, IMP-3, IMP3, KOC, KOC1, VIC | insulin like growth factor 2 mRNA binding protein 3 |
| F7 | SPCA | coagulation factor VII |
| SFN | YWHAS | stratifin |
| FSCN1 | FAN1, HSN, SNL, p55 | fascin actin-bundling protein 1 |
| BMP7 | OP-1 | bone morphogenetic protein 7 |
| PRNP | ASCR, AltPrP, CD230, CJD, GSS, KUI | prion protein |
| NOTCH3 | CADASIL, CADASIL1, CASIL, IMF2, L | notch receptor 3 |
| CALR | CRT, HEL-S-99n, RO, SSA, cC1qR | calreticulin |
| SMAD7 | CRCS3, MADH7, MADH8 | SMAD family member 7 |
| INS | IDDM, IDDM1, IDDM2, ILPR, IRDN, | insulin |
| CSNK2A1 | CK2A1, CKII, Cka1, Cka2, OCNDS | casein kinase 2 alpha 1 |
| MIR222 | MIRN222, miRNA222, mir-222 | microRNA 222 |
| IL2 | IL-2, TCGF, lymphokine | interleukin 2 |
| GPX1 | GPXD, GSHPX1 | glutathione peroxidase 1 |
| MIR210 | MIRN210, mir-210 | microRNA 210 |
| MIR125B1 | MIRN125B1, mir-125b-1 | microRNA 125b-1 |
| H2AX | H2A.X, H2A/X, H2AFX | H2A.X variant histone |
| RAF1 | CMD1NN, CRAF, NS5, Raf-1, c-Raf | Raf-1 proto-oncogene, serine/threonine kinase |
| MPO | | myeloperoxidase |

| | | |
|---|---|---|
| MIR195 | MIRN195, miRNA195, mir-195 | microRNA 195 |
| SMARCA4 | BAF190, BAF190A, BRG1, CSS4, MR | SWI/SNF related, matrix associated, actin dependent regulator c |
| FLT4 | CHTD7, FLT-41, LMPH1A, LMPHM1 | fms related receptor tyrosine kinase 4 |
| PVT1 | LINC00079, MIR1204HG, NCRNA00 | Pvt1 oncogene |
| FOXC1 | ARA, ASGD3, FKHL7, FREAC-3, FREA | forkhead box C1 |
| IDH1 | HEL-216, HEL-S-26, IDCD, IDH, IDP, | isocitrate dehydrogenase (NADP(+)) 1 |
| CXCR3 | CD182, CD183, CKR-L2, CMKAR3, G | C-X-C motif chemokine receptor 3 |
| MIR101-1 | MIRN101-1, mir-101-1 | microRNA 101-1 |
| CTSB | APPS, CPSB, RECEUP | cathepsin B |
| RARB | HAP, MCOPS12, NR1B2, RARbeta1, | retinoic acid receptor beta |
| UCHL1 | HEL-117, HEL-S-53, NDGOA, PARK5 | ubiquitin C-terminal hydrolase L1 |
| FOS | AP-1, C-FOS, p55 | Fos proto-oncogene, AP-1 transcription factor subunit |
| MIR31 | MIRN31, hsa-mir-31, miR-31 | microRNA 31 |
| FOXQ1 | HFH1 | forkhead box Q1 |
| CD40 | Bp50, CDW40, TNFRSF5, p50 | CD40 molecule |
| PDGFRB | CD140B, IBGC4, IMF1, JTK12, KOGS | platelet derived growth factor receptor beta |
| FYN | SLK, SYN, p59-FYN | FYN proto-oncogene, Src family tyrosine kinase |
| CBL | C-CBL2, FRA11B, NSLL, RNF55, CBL | Cbl proto-oncogene |
| NLRP3 | AGTAVPRL, AII, AVP, C1orf7, CIAS1 | NLR family pyrin domain containing 3 |
| TNFRSF1A | CD120a, FPF, TBP1, TNF-R, TNF-R-I | TNF receptor superfamily member 1A |
| STAT5A | MGF, STAT5 | signal transducer and activator of transcription 5A |
| UBE2I | C358B7.1, P18, UBC9 | ubiquitin conjugating enzyme E2 I |
| GPC3 | DGSX, GTR2-2, MXR7, OCI-5, SDYS, | glypican 3 |
| EPOR | EPO-R | erythropoietin receptor |
| EPO | DBAL, ECYT5, EP, MVCD2 | erythropoietin |
| LGR5 | FEX, GPR49, GPR67, GRP49, HG38 | leucine rich repeat containing G protein-coupled receptor 5 |
| PML | MYL, PP8675, RNF71, TRIM19 | promyelocytic leukemia |
| TLR3 | CD283, IIAE2 | toll like receptor 3 |
| GRB2 | ASH, EGFRBP-GRB2, Grb3-3, MST0 | growth factor receptor bound protein 2 |
| XBP1 | TREB-5, TREB5, XBP-1, XBP2 | X-box binding protein 1 |
| KEAP1 | INrf2, KLHL19 | kelch like ECH associated protein 1 |
| CD14 | | CD14 molecule |
| HP | BP2ALPHA2, HPA1S, HP | haptoglobin |
| STAT5B | STAT5 | signal transducer and activator of transcription 5B |
| MIR122 | MIR122A, MIRN122, MIRN122A, hs | microRNA 122 |
| KLF5 | BTEB2, CKLF, IKLF | Kruppel like factor 5 |
| KLK3 | APS, KLK2A1, PSA, hK3 | kallikrein related peptidase 3 |
| VEGFD | FIGF, VEGF-D | vascular endothelial growth factor D |
| VHL | HRCA1, RCA11, pVHL, VHL | von Hippel-Lindau tumor suppressor |
| PHB | HEL-215, HEL-S-54e1, PHB | prohibitin |
| MUC16 | CA125 | mucin 16, cell surface associated |
| MECP2 | AUTSX3, MRX16, MRX79, MRXS13, | methyl-CpG binding protein 2 |
| CAT | | catalase |
| TFPI2 | PP5, REF1, TFPI-2 | tissue factor pathway inhibitor 2 |
| RACK1 | GNB2L1, Gnb2-rs1, H12.3, HLC-7, P | receptor for activated C kinase 1 |
| MIR125A | MIRN125A, miRNA125A, mir-125a | microRNA 125a |
| FABP4 | A-FABP, AFABP, ALBP, HEL-S-104, a | fatty acid binding protein 4 |
| CCR2 | CC-CKR-2, CCR-2A, CCR2B, CD192, | C-C motif chemokine receptor 2 |
| MIR137 | MIRN137, miR-137 | microRNA 137 |
| VCAN | CSPG2, ERVR, GHAP, PG-M, WGN, | versican |
| MIR26A1 | MIR26A, MIRN26A1, mir-26a-1 | microRNA 26a-1 |

| | | |
|---|---|---|
| PTPN1 | PTP1B | protein tyrosine phosphatase non-receptor type 1 |
| HTT | HD, IT15, LOMARS | huntingtin |
| APOBEC3( | A3G, ARCD, ARP-9, ARP9, CEM-15, | apolipoprotein B mRNA editing enzyme catalytic subunit 3G |
| SERPINF1 | EPC-1, OI12, OI6, PEDF, PIG35 | serpin family F member 1 |
| MIR183 | MIRN183, miR-183, miRNA183 | microRNA 183 |
| ALDH2 | ALDH-E2, ALDHI, ALDM | aldehyde dehydrogenase 2 family member |
| MMP8 | CLG1, HNC, MMP-8, PMNL-CL | matrix metallopeptidase 8 |
| BST2 | CD317, TETHERIN | bone marrow stromal cell antigen 2 |
| ITGA3 | CD49C, FRP-2, GAP-B3, GAPB3, ILN | integrin subunit alpha 3 |
| CSF1 | CSF-1, MCSF | colony stimulating factor 1 |
| CYP3A5 | CP35, CYPIIIA5, P450PCN3, PCN3 | cytochrome P450 family 3 subfamily A member 5 |
| SIRT3 | SIR2L3 | sirtuin 3 |
| BCL6 | BCL5A, LAZ3, ZBTB27, ZNF51, BCL6 | BCL6 transcription repressor |
| PDCD4 | H731 | programmed cell death 4 |
| IL11 | AGIF, IL-11 | interleukin 11 |
| HAVCR2 | CD366, HAVcr-2, KIM-3, SPTCL, TIM | hepatitis A virus cellular receptor 2 |
| MIR223 | MIRN223, miRNA223, mir-223 | microRNA 223 |
| DPP4 | ADABP, ADCP2, CD26, DPPIV, TP10 | dipeptidyl peptidase 4 |
| XRCC5 | KARP-1, KARP1, KU80, KUB2, Ku86, | X-ray repair cross complementing 5 |
| FOXC2 | FKHL14, LD, MFH-1, MFH1 | forkhead box C2 |
| MSN | HEL70, IMD50 | moesin |
| ADM | AM, PAMP | adrenomedullin |
| MIR93 | MIRN9, MIRN93, hsa-mir-93, miR-9 | microRNA 93 |
| HDAC2 | HD2, KDAC2, RPD3, YAF1 | histone deacetylase 2 |
| DAPK1 | DAPK, ROCO3 | death associated protein kinase 1 |
| TRAF6 | MGC:3310, RNF85 | TNF receptor associated factor 6 |
| TARDBP | ALS10, TDP-43 | TAR DNA binding protein |
| EREG | EPR, ER, Ep | epiregulin |
| KL | HFTC3 | klotho |
| TGFBI | BIGH3, CDB1, CDG2, CDGG1, CSD, ( | transforming growth factor beta induced |
| HSP90B1 | ECGP, GP96, GRP94, HEL-S-125m, I | heat shock protein 90 beta family member 1 |
| EIF4EBP1 | 4E-BP1, 4EBP1, BP-1, PHAS-I | eukaryotic translation initiation factor 4E binding protein 1 |
| CIP2A | KIAA1524, p90 | cellular inhibitor of PP2A |
| NT5E | CALJA, CD73, E5NT, NT, NT5, NTE, ( | 5'-nucleotidase ecto |
| ANGPTL4 | ARP4, FIAF, HARP, HFARP, NL2, PG | angiopoietin like 4 |
| MIR24-1 | MIR189, MIRN24-1, miR-24-1, miR | microRNA 24-1 |
| NAT1 | AAC1, MNAT, NAT-1, NATI | N-acetyltransferase 1 |
| NGFR | CD271, Gp80-LNGFR, TNFRSF16, p7 | nerve growth factor receptor |
| SELP | CD62, CD62P, GMP140, GRMP, LEC | selectin P |
| PRLR | HPRL, MFAB, RI-PRLR, hPRLrI | prolactin receptor |
| CADM1 | BL2, IGSF4, IGSF4A, NECL2, Necl-2, | cell adhesion molecule 1 |
| TXN | TRDX, TRX, TRX1 | thioredoxin |
| DNMT3A | DNMT3A2, HESJAS, M.HsaIIIA, TBR | DNA methyltransferase 3 alpha |
| ITGA2 | BR, CD49B, GPIa, HPA-5, VLA-2, VL | integrin subunit alpha 2 |
| NR1I2 | BXR, ONR1, PAR, PAR1, PAR2, PAR( | nuclear receptor subfamily 1 group I member 2 |
| TNFRSF1B | CD120b, TBPII, TNF-R-II, TNF-R75, T | TNF receptor superfamily member 1B |
| IL13 | IL-13, P600 | interleukin 13 |
| PDGFB | IBGC5, PDGF-2, PDGF2, SIS, SSV, c- | platelet derived growth factor subunit B |
| CCL18 | AMAC-1, AMAC1, CKb7, DC-CK1, D | C-C motif chemokine ligand 18 |
| PIM1 | PIM | Pim-1 proto-oncogene, serine/threonine kinase |
| RANBP9 | BPM-L, BPM90, RANBPM, RanBP7 | RAN binding protein 9 |

| | | |
|---|---|---|
| TACR1 | NK1R, NKIR, SPR, TAC1R | tachykinin receptor 1 |
| GAS5 | NCRNA00030, SNHG2 | growth arrest specific 5 |
| GNB3 | CSNB1H | G protein subunit beta 3 |
| P2RX7 | P2X7 | purinergic receptor P2X 7 |
| STIM1 | D11S4896E, GOK, IMD10, STRMK, T | stromal interaction molecule 1 |
| NOTCH2 | AGS2, HJCYS, hN2 | notch receptor 2 |
| NGF | Beta-NGF, HSAN5B, NGF | nerve growth factor |
| MUC4 | ASGP, HSA276359, MUC-4 | mucin 4, cell surface associated |
| PTX3 | TNFAIP5, TSG-14 | pentraxin 3 |
| YWHAZ | 14-3-3-zeta, HEL-S-3, HEL-S-93, HEI | tyrosine 3-monooxygenase/tryptophan 5-monooxygenase activa |
| HAMP | HEPC, HFE2B, LEAP1, PLTR | hepcidin antimicrobial peptide |
| SHC1 | SHC, SHCA | SHC adaptor protein 1 |
| STAT6 | D12S1644, IL-4-STATB, STAT6C, ST/ | signal transducer and activator of transcription 6 |
| S100A7 | PSOR1c, S100A7 | S100 calcium binding protein A7 |
| CASP9 | APAF-3, APAF3, ICE-LAP6, MCH6, P | caspase 9 |
| CDH5 | 7B4, CD144 | cadherin 5 |
| ST14 | ARCI11, HAI, MT-SP1, MTSP1, PRSS | suppression of tumorigenicity 14 |
| IGFBP1 | AFBP, IBP1, IGF-BP25, PP12, hIGFB | insulin like growth factor binding protein 1 |
| MIR139 | MIR139-3p, MIRN139, mir-139 | microRNA 139 |
| PTCH1 | BCNS, NBCCS, PTC, PTC1, PTCH | patched 1 |
| FANCD2 | FA-D2, FA4, FACD, FAD, FAD2, FAN | FA complementation group D2 |
| PSCA | PRO232 | prostate stem cell antigen |
| DLC1 | ARHGAP7, HP, STARD12, p122-Rho | DLC1 Rho GTPase activating protein |
| LIN28A | CSDD1, LIN-28, LIN28, ZCCHC1, lin- | lin-28 homolog A |
| ROCK2 | ROCK-II | Rho associated coiled-coil containing protein kinase 2 |
| IRF1 | IRF-1, MAR | interferon regulatory factor 1 |
| PTK2B | CADTK, CAKB, FADK2, FAK2, PKB, P | protein tyrosine kinase 2 beta |
| CTH | | cystathionine gamma-lyase |
| MIR196A2 | MIRN196-2, MIRN196A2, mir-196a | microRNA 196a-2 |
| MMP11 | SL-3, ST3, STMY3 | matrix metallopeptidase 11 |
| PRKCI | DXS1179E, PKCI, nPKC-iota | protein kinase C iota |
| CD34 | | CD34 molecule |
| FLNA | ABP-280, ABPX, CSBS, CVD1, FGS2, | filamin A |
| TNFAIP3 | A20, AISBL, OTUD7C, TNFA1P2 | TNF alpha induced protein 3 |
| MIR375 | MIRN375, hsa-mir-375, miRNA375 | microRNA 375 |
| PFN1 | ALS18 | profilin 1 |
| MIR20A | C13orf25, MIR17HG, MIR20, MIRH | microRNA 20a |
| MDC1 | NFBD1 | mediator of DNA damage checkpoint 1 |
| RXRA | NR2B1 | retinoid X receptor alpha |
| STUB1 | CHIP, HSPABP2, NY-CO-7, SCA48, S | STIP1 homology and U-box containing protein 1 |
| MAP2K1 | CFC3, MAPKK1, MEK1, MKK1, PRKM | mitogen-activated protein kinase kinase 1 |
| GSN | ADF, AGEL | gelsolin |
| GALC | | galactosylceramidase |
| HSPA4 | APG-2, HEL-S-5a, HS24/P52, HSPH2 | heat shock protein family A (Hsp70) member 4 |
| CD276 | 4Ig-B7-H3, B7-H3, B7H3, B7RP-2 | CD276 molecule |
| PAK4 | | p21 (RAC1) activated kinase 4 |
| PTPN6 | HCP, HCPH, HPTP1C, PTP-1C, SH-PT | protein tyrosine phosphatase non-receptor type 6 |
| SREBF1 | SREBP1, bHLHd1 | sterol regulatory element binding transcription factor 1 |
| TNFRSF6B | DCR3, DJ583P15.1.1, M68, M68E, T | TNF receptor superfamily member 6b |
| CAMP | CAP-18, CAP18, CRAMP, FALL-39, F | cathelicidin antimicrobial peptide |
| SFRP1 | FRP, FRP-1, FRP1, FrzA, SARP2 | secreted frizzled related protein 1 |

| | | |
|---|---|---|
| MIR335 | MIRN335, hsa-mir-335, miRNA335, | microRNA 335 |
| AKT3 | MPPH, MPPH2, PKB-GAMMA, PKBG | AKT serine/threonine kinase 3 |
| SAA1 | PIG4, SAA, SAA2, TP53I4 | serum amyloid A1 |
| MIR199A1 | MIR-199-s, MIRN199A1, mir-199a- | microRNA 199a-1 |
| TXNIP | ARRDC6, EST01027, HHCPA78, THI | thioredoxin interacting protein |
| KRT5 | CK5, DDD, DDD1, EBS2, K5A, KRT5 | keratin 5 |
| BMP6 | VGR, VGR1 | bone morphogenetic protein 6 |
| BIRC7 | KIAP, LIVIN, ML-IAP, MLIAP, RNF50 | baculoviral IAP repeat containing 7 |
| MAP1LC3A | ATG8E, LC3, LC3A, MAP1ALC3, MA | microtubule associated protein 1 light chain 3 alpha |
| PGF | D12S1900L, PIGF, PLGF, PlGF-2, SH | placental growth factor |
| GHR | GHBP, GHIP | growth hormone receptor |
| IKBKB | IKK-beta, IKK2, IKKB, IMD15, IMD1 | inhibitor of nuclear factor kappa B kinase subunit beta |
| BRD4 | CAP, HUNK1, HUNKI, MCAP | bromodomain containing 4 |
| CALCA | CALC1, CGRP, CGRP-I, CGRP-alpha, | calcitonin related polypeptide alpha |
| LEF1 | LEF-1, TCF10, TCF1ALPHA, TCF7L3 | lymphoid enhancer binding factor 1 |
| PRDX1 | MSP23, NKEF-A, NKEFA, PAG, PAG | peroxiredoxin 1 |
| ARID1A | B120, BAF250, BAF250a, BM029, C | AT-rich interaction domain 1A |
| DLL4 | AOS6, delta4, hdelta2 | delta like canonical Notch ligand 4 |
| TGFB2 | G-TSF, LDS4, TGF-beta2 | transforming growth factor beta 2 |
| MIR9-1 | MIRN9-1, hsa-mir-9-1, miRNA9-1, r | microRNA 9-1 |
| ENO1 | ENO1L1, HEL-S-17, MPB1, NNE, PP | enolase 1 |
| TRAF2 | MGC:45012, RNF117, TRAP, TRAP3 | TNF receptor associated factor 2 |
| CTCF | CFAP108, FAP108, MRD21 | CCCTC-binding factor |
| OSM | | oncostatin M |
| CDX2 | CDX-3/AS, CDX3, CDX2 | caudal type homeobox 2 |
| IL32 | IL-32alpha, IL-32beta, IL-32delta, IL | interleukin 32 |
| LHCGR | HHG, LCGR, LGR2, LH/CG-R, LH/CG | luteinizing hormone/choriogonadotropin receptor |
| IL15 | IL-15 | interleukin 15 |
| HTRA1 | ARMD7, CADASIL2, CARASIL, HtrA, | HtrA serine peptidase 1 |
| HBEGF | DTR, DTS, DTSF, HEGFL | heparin binding EGF like growth factor |
| G6PD | G6PD1 | glucose-6-phosphate dehydrogenase |
| S100P | MIG9 | S100 calcium binding protein P |
| APOBEC3B | A3B, APOBEC1L, ARCD3, ARP4, DJ7 | apolipoprotein B mRNA editing enzyme catalytic subunit 3B |
| ITGB2 | CD18, LAD, LCAMB, LFA-1, MAC-1, | integrin subunit beta 2 |
| CCNA2 | CCN1, CCNA | cyclin A2 |
| PPM1D | IDDGIP, JDVS, PP2C-DELTA, WIP1 | protein phosphatase, Mg2+/Mn2+ dependent 1D |
| VTCN1 | B7-H4, B7H4, B7S1, B7X, B7h.5, PR | V-set domain containing T cell activation inhibitor 1 |
| NCL | C23, Nsr1 | nucleolin |
| NEK2 | HsPK21A, NLK1, PPP1R111, RP67, N | NIMA related kinase 2 |
| DCN | CSCD, DSPG2, PG40, PGII, PGS2, SL | decorin |
| GZMB | C11, CCPI, CGL-1, CGL1, CSP-B, CSP | granzyme B |
| PPIA | CYPA, CYPH, HEL-S-69p | peptidylprolyl isomerase A |
| TUBB3 | CDCBM, CDCBM1, CFEOM3, CFEOM | tubulin beta 3 class III |
| NOTCH4 | INT3 | notch receptor 4 |
| IQGAP1 | HUMORFA01, SAR1, p195 | IQ motif containing GTPase activating protein 1 |
| PAX5 | ALL3, BSAP | paired box 5 |
| PPP2CA | NEDLBA, PP2Ac, PP2CA, PP2Calpha | protein phosphatase 2 catalytic subunit alpha |
| MIR96 | DFNA50, MIRN96, hsa-mir-96, miR | microRNA 96 |
| XRCC4 | SSMED | X-ray repair cross complementing 4 |
| LINC-ROR | ROR, lincRNA-RoR, lincRNA-ST8SIA | long intergenic non-protein coding RNA, regulator of reprogramm |
| FAP | DPPIVA, FAPalpha, SIMP, FAP | fibroblast activation protein alpha |

| | | |
|---|---|---|
| TRPM7 | ALSPDC, CHAK, CHAK1, LTRPC7, LT | transient receptor potential cation channel subfamily M membe |
| IRS2 | IRS-2 | insulin receptor substrate 2 |
| NF2 | ACN, BANF, SCH | neurofibromin 2 |
| TRIM28 | KAP1, PPP1R157, RNF96, TF1B, TIF | tripartite motif containing 28 |
| RPS6KA3 | CLS, HU-3, ISPK-1, MAPKAPK1B, M | ribosomal protein S6 kinase A3 |
| MIR29C | MIRN29C, miRNA29C, mir-29c | microRNA 29c |
| PLG | | plasminogen |
| IL27 | IL-27, IL-27AA, IL27p28, IL30, p28, | interleukin 27 |
| BAD | BBC2, BCL2L8 | BCL2 associated agonist of cell death |
| SALL4 | DRRS, HSAL4, ZNF797 | spalt like transcription factor 4 |
| S100B | NEF, S100, S100-B, S100beta | S100 calcium binding protein B |
| MAP3K7 | CSCF, FMD2, MEKK7, TAK1, TGF1a | mitogen-activated protein kinase kinase kinase 7 |
| LRP5 | BMND1, EVR1, EVR4, HBM, LR3, LR | LDL receptor related protein 5 |
| TET1 | CXXC6, LCX, bA119F7.1 | tet methylcytosine dioxygenase 1 |
| AGO2 | CASC7, EIF2C2, LINC00980, PPD, Q | argonaute RISC catalytic component 2 |
| PRKCE | PKCE, nPKC-epsilon | protein kinase C epsilon |
| NFATC2 | NFAT1, NFATP | nuclear factor of activated T cells 2 |
| MIR29B1 | MIRN29B1, miR-29b, miRNA29B1, | microRNA 29b-1 |
| NEDD9 | CAS-L, CAS2, CASL, CASS2, HEF1 | neural precursor cell expressed, developmentally down-regulate |
| HOXB13 | HPC9, PSGD | homeobox B13 |
| AURKB | AIK2, AIM-1, AIM1, ARK-2, ARK2, A | aurora kinase B |
| ORAI1 | CRACM1, IMD9, ORAT1, TAM2, TM | ORAI calcium release-activated calcium modulator 1 |
| ACE2 | ACEH | angiotensin I converting enzyme 2 |
| NCOA1 | F-SRC-1, KAT13A, RIP160, SRC1, bH | nuclear receptor coactivator 1 |
| PRKCZ | PKC-ZETA, PKC2 | protein kinase C zeta |
| EFEMP1 | DHRD, DRAD, FBLN3, FBNL, FIBL-3, | EGF containing fibulin extracellular matrix protein 1 |
| DUSP1 | CL100, HVH1, MKP-1, MKP1, PTPN | dual specificity phosphatase 1 |
| PAX6 | AN, AN1, AN2, ASGD5, D11S812E, | paired box 6 |
| TFPI | EPI, LACI, TFI1, TFPI | tissue factor pathway inhibitor |
| CCR6 | BN-1, C-C CKR-6, CC-CKR-6, CCR-6, | C-C motif chemokine receptor 6 |
| FOSL1 | FRA, FRA1, fra-1 | FOS like 1, AP-1 transcription factor subunit |
| ELMO1 | CED-12, CED12, ELMO-1 | engulfment and cell motility 1 |
| CD151 | GP27, MER2, PETA-3, RAPH, SFA1, | CD151 molecule (Raph blood group) |
| NR5A2 | B1F, B1F2, CPF, FTF, FTZ-F1, FTZ-F1 | nuclear receptor subfamily 5 group A member 2 |
| CD163 | M130, MM130, SCARI1 | CD163 molecule |
| KPNA2 | IPOA1, QIP2, RCH1, SRP1-alpha, SR | karyopherin subunit alpha 2 |
| PAWR | PAR4, Par-4 | pro-apoptotic WT1 regulator |
| DIABLO | DFNA64, SMAC | diablo IAP-binding mitochondrial protein |
| CCDC88A | APE, GIRDIN, GIV, GRDN, HkRP1, K | coiled-coil domain containing 88A |
| SUMO1 | DAP1, GMP1, OFC10, PIC1, SENP2, | small ubiquitin like modifier 1 |
| LTF | GIG12, HEL110, HLF2, LF | lactotransferrin |
| LASP1 | Lasp-1, MLN50 | LIM and SH3 protein 1 |
| MIR100 | MIRN100, miR-100 | microRNA 100 |
| CTSK | CTS02, CTSO, CTSO1, CTSO2, PKND | cathepsin K |
| C1QBP | COXPD33, GC1QBP, HABP1, SF2AP | complement C1q binding protein |
| ENPP2 | ATX, ATX-X, AUTOTAXIN, LysoPLD, | ectonucleotide pyrophosphatase/phosphodiesterase 2 |
| MDK | ARAP, MK, NEGF2 | midkine |
| PROCR | CCCA, CCD41, EPCR | protein C receptor |
| SOST | CDD, DAND61, VBCH, SOST | sclerostin |
| COL18A1 | KNO, KNO1, KS | collagen type XVIII alpha 1 chain |
| MYH9 | BDPLT6, DFNA17, EPSTS, FTNS, MA | myosin heavy chain 9 |

| | | |
|---|---|---|
| PRMT1 | ANM1, HCP1, HRMT1L2, IR1B4 | protein arginine methyltransferase 1 |
| MIR16-1 | MIRN16-1, miRNA16-1, mir-16-1 | microRNA 16-1 |
| MSLN | MPF, SMRP | mesothelin |
| CUL1 | | cullin 1 |
| LOXL2 | LOR, LOR2, WS9-14 | lysyl oxidase like 2 |
| HSPD1 | CPN60, GROEL, HLD4, HSP-60, HSP | heat shock protein family D (Hsp60) member 1 |
| XIST | DXS1089, DXS399E, LINC00001, NC | X inactive specific transcript |
| TSG101 | TSG10, VPS23 | tumor susceptibility 101 |
| DDR1 | CAK, CD167, DDR, EDDR1, HGK2, M | discoidin domain receptor tyrosine kinase 1 |
| CCND2 | KIAK0002, MPPH3 | cyclin D2 |
| BAK1 | BAK, BAK-LIKE, BCL2L7, CDN1 | BCL2 antagonist/killer 1 |
| TSC2 | LAM, PPP1R160, TSC4 | TSC complex subunit 2 |
| ANGPT1 | AGP1, AGPT, ANG1 | angiopoietin 1 |
| PROC | APC, PC1, THPH3, THPH4, PROC | protein C, inactivator of coagulation factors Va and VIIIa |
| LGALS3BP | 90K, BTBD17B, CyCAP, M2BP, MAC | galectin 3 binding protein |
| CSF1R | BANDDOS, C-FMS, CD115, CSF-1R, | colony stimulating factor 1 receptor |
| LYN | JTK8, p53Lyn, p56Lyn | LYN proto-oncogene, Src family tyrosine kinase |
| ARHGDIA | GDIA1, HEL-S-47e, NPHS8, RHOGD | Rho GDP dissociation inhibitor alpha |
| PSMD10 | dJ889N15.2, p28, p28(GANK) | proteasome 26S subunit, non-ATPase 10 |
| WNT3A | | Wnt family member 3A |
| CTSL | CATL1, MEP, CTSL | cathepsin L |
| IL4R | CD124, IL-4RAA, IL4R | interleukin 4 receptor |
| ARF6 | | ADP ribosylation factor 6 |
| CDK5 | LIS7, PSSALRE | cyclin dependent kinase 5 |
| THY1 | CD90, CDw90 | Thy-1 cell surface antigen |
| CDK9 | C-2k, CDC2L4, CTK1, PITALRE, TAK | cyclin dependent kinase 9 |
| ADAR | ADAR1, AGS6, DRADA, DSH, DSRAD | adenosine deaminase RNA specific |
| MAPK7 | BMK1, ERK4, ERK5, PRKM7 | mitogen-activated protein kinase 7 |
| PKD1 | PBP, PC1, Pc-1, TRPP1 | polycystin 1, transient receptor potential channel interacting |
| NDRG2 | SYLD | NDRG family member 2 |
| RAN | ARA24, Gsp1, TC4 | RAN, member RAS oncogene family |
| OGT | HINCUT-1, HRNT1, MRX106, O-GLC | O-linked N-acetylglucosamine (GlcNAc) transferase |
| SIX1 | BOS3, DFNA23, TIP39 | SIX homeobox 1 |
| AQP5 | AQP-5, PPKB | aquaporin 5 |
| OLR1 | CLEC8A, LOX1, LOXIN, SCARE1, SLC | oxidized low density lipoprotein receptor 1 |
| CD74 | DHLAG, HLADG, II, Ia-GAMMA, p33 | CD74 molecule |
| FGB | HEL-S-78p | fibrinogen beta chain |
| FGF3 | HBGF-3, INT2 | fibroblast growth factor 3 |
| GATA4 | ASD2, TACHD, TOF, VSD1 | GATA binding protein 4 |
| LIFR | CD118, LIF-R, SJS2, STWS, SWS | LIF receptor subunit alpha |
| ACP5 | HPAP, TRACP5a, TRACP5b, TRAP, T | acid phosphatase 5, tartrate resistant |
| CX3CL1 | ABCD-3, C3Xkine, CXC3, CXC3C, NT | C-X3-C motif chemokine ligand 1 |
| CDH3 | CDHP, HJMD, PCAD | cadherin 3 |
| FOXP1 | 12CC4, HSPC215, MFH, QRF1, hFKH | forkhead box P1 |
| NCOR2 | CTG26, N-CoR2, SMAP270, SMRT, S | nuclear receptor corepressor 2 |
| TFF3 | ITF, P1B, TFI | trefoil factor 3 |
| DPYD | DHP, DHPDHASE, DPD | dihydropyrimidine dehydrogenase |
| F11R | CD321, JAM, JAM1, JAMA, JCAM, K | F11 receptor |
| MECOM | AML1-EVI-1, EVI1, KMT8E, MDS1, M | MDS1 and EVI1 complex locus |
| DDR2 | MIG20a, NTRKR3, TKT, TYRO10, W | discoidin domain receptor tyrosine kinase 2 |
| CCL20 | CKb4, Exodus, LARC, MIP-3-alpha, | C-C motif chemokine ligand 20 |

| | | |
|---|---|---|
| TRIO | ARHGEF23, MEBAS, MRD44, tgat | trio Rho guanine nucleotide exchange factor |
| TGFA | TFGA | transforming growth factor alpha |
| INHBA | EDF, FRP | inhibin subunit beta A |
| PTPA | PP2A, PPP2R4, PR53 | protein phosphatase 2 phosphatase activator |
| FHL2 | AAG11, DRAL, FHL-2, SLIM-3, SLIM | four and a half LIM domains 2 |
| FGF1 | AFGF, ECGF, ECGF-beta, ECGFA, EC | fibroblast growth factor 1 |
| MEN1 | MEAI, SCG2 | menin 1 |
| MIR19A | C13orf25, MIR17HG, MIRH1, MIRH | microRNA 19a |
| SCD | FADS5, MSTP0081, SCDOS, hSCD1, | stearoyl-CoA desaturase |
| SIN3A | WITKOS | SIN3 transcription regulator family member A |
| XRCC2 | FANCU | X-ray repair cross complementing 2 |
| ANXA3 | ANX3 | annexin A3 |
| UCP2 | BMIQ4, SLC25A8, UCPH | uncoupling protein 2 |
| KRT8 | CARD2, CK-8, CK8, CYK8, K2C8, K8, | keratin 8 |
| AQP1 | AQP-CHIP, CHIP28, CO | aquaporin 1 (Colton blood group) |
| CLOCK | KAT13D, bHLHe8 | clock circadian regulator |
| DDIT3 | AltDDIT3, C/EBPzeta, CEBPZ, CHOP | DNA damage inducible transcript 3 |
| TUG1 | LINC00080, NCRNA00080, TI-227H | taurine up-regulated 1 |
| ALOX15 | 12-LOX, 15-LOX, 15-LOX-1, LOG15 | arachidonate 15-lipoxygenase |
| RAB5A | RAB5 | RAB5A, member RAS oncogene family |
| LAPTM4B | LAPTM4beta, LC27 | lysosomal protein transmembrane 4 beta |
| IKBKG | AMCBX1, EDAID1, FIP-3, FIP3, Fip3 | inhibitor of nuclear factor kappa B kinase regulatory subunit gan |
| PITX2 | ARP1, ASGD4, Brx1, IDG2, IGDS, IG | paired like homeodomain 2 |
| SDCBP | MDA-9, MDA9, ST1, SYCL, TACIP18 | syndecan binding protein |
| PPARD | FAAR, NR1C2, NUC1, NUCI, NUCII, | peroxisome proliferator activated receptor delta |
| SLC16A1 | HHF7, MCT, MCT1, MCT1D | solute carrier family 16 member 1 |
| PELP1 | MNAR, P160 | proline, glutamate and leucine rich protein 1 |
| CD55 | CHAPLE, CR, CROM, DAF, TC | CD55 molecule (Cromer blood group) |
| VASP | | vasodilator stimulated phosphoprotein |
| PIK3CB | P110BETA, PI3K, PI3KBETA, PIK3C1 | phosphatidylinositol-4,5-bisphosphate 3-kinase catalytic subunit |
| MIR138-1 | MIRN138-1, mir-138-1 | microRNA 138-1 |
| ITGA4 | CD49D, IA4 | integrin subunit alpha 4 |
| MIR218-1 | MIRN218-1, mir-218-1 | microRNA 218-1 |
| NCOA2 | GRIP1, KAT13C, NCoA-2, SRC2, TIF2 | nuclear receptor coactivator 2 |
| MIR140 | MIRN140, SEDN, miRNA140, mir-14 | microRNA 140 |
| AKR1C3 | DD3, DDX, HA1753, HAKRB, HAKRe | aldo-keto reductase family 1 member C3 |
| FADD | GIG3, MORT1 | Fas associated via death domain |
| BBC3 | JFY-1, JFY1, PUMA | BCL2 binding component 3 |
| IKBKE | IKK-E, IKK-i, IKKE, IKKI | inhibitor of nuclear factor kappa B kinase subunit epsilon |
| JAK1 | JAK1AB, JTK3, JAK1 | Janus kinase 1 |
| THRB | C-ERBA-2, C-ERBA-BETA, ERBA2, G | thyroid hormone receptor beta |
| EHMT2 | BAT8, C6orf30, G9A, GAT8, KMT1C | euchromatic histone lysine methyltransferase 2 |
| GAPDH | G3PD, GAPD, HEL-S-162eP | glyceraldehyde-3-phosphate dehydrogenase |
| MIR149 | MIRN149, mir-149 | microRNA 149 |
| GADD45A | DDIT1, GADD45 | growth arrest and DNA damage inducible alpha |
| CSF2 | CSF, GMCSF | colony stimulating factor 2 |
| BIRC2 | API1, HIAP2, Hiap-2, MIHB, RNF48, | baculoviral IAP repeat containing 2 |
| SGK1 | SGK | serum/glucocorticoid regulated kinase 1 |
| TACSTD2 | EGP-1, EGP1, GA733-1, GA7331, G | tumor associated calcium signal transducer 2 |
| MIR106B | MIRN106B, mir-106b | microRNA 106b |
| TRPS1 | GC79, LGCR | transcriptional repressor GATA binding 1 |

| | | |
|---|---|---|
| ALOX5 | 5-LO, 5-LOX, 5LPG, LOG5 | arachidonate 5-lipoxygenase |
| MIR451A | MIR451, MIRN451, hsa-mir-451, hs | microRNA 451a |
| KLF6 | BCD1, CBA1, COPEB, CPBP, GBF, PA | Kruppel like factor 6 |
| IL1RL1 | DER4, FIT-1, IL33R, ST2, ST2L, ST2V | interleukin 1 receptor like 1 |
| TAC1 | Hs.2563, NK2, NKNA, NPK, TAC2 | tachykinin precursor 1 |
| FABP5 | E-FABP, EFABP, KFABP, PA-FABP, P | fatty acid binding protein 5 |
| NRP2 | NP2, NPN2, PRO2714, VEGF165R2 | neuropilin 2 |
| MIR107 | MIRN107, miR-107 | microRNA 107 |
| CDCP1 | CD318, SIMA135, TRASK | CUB domain containing protein 1 |
| DKK3 | REIC, RIG | dickkopf WNT signaling pathway inhibitor 3 |
| ACTN4 | ACTININ-4, FSGS, FSGS1 | actinin alpha 4 |
| SMYD3 | KMT3E, ZMYND1, ZNFN3A1, bA74P | SET and MYND domain containing 3 |
| IL12A | CLMF, IL-12A, NFSK, NKSF1, P35 | interleukin 12A |
| MAP1LC3I | ATG8F, LC3B, MAP1A/1BLC3-a, MA | microtubule associated protein 1 light chain 3 beta |
| MIR320A | MIRN320, MIRN320A, hsa-mir-320 | microRNA 320a |
| TNFRSF10 | APO2, CD261, DR4, TRAILR-1, TRAI | TNF receptor superfamily member 10a |
| LAMC2 | B2T, BM600, CSF, EBR2, EBR2A, LA | laminin subunit gamma 2 |
| ALPL | AP-TNAP, APTNAP, HOPS, TNALP, T | alkaline phosphatase, biomineralization associated |
| PRMT5 | HRMT1L5, HSL7, IBP72, JBP1, SKB1 | protein arginine methyltransferase 5 |
| HES1 | HES-1, HHL, HRY, bHLHb39 | hes family bHLH transcription factor 1 |
| KAT5 | ESA1, HTATIP, HTATIP1, PLIP, TIP, T | lysine acetyltransferase 5 |
| HLA-E | HLA-6.2, QA1 | major histocompatibility complex, class I, E |
| FST | FS | follistatin |
| IL23A | IL-23, IL-23A, IL23P19, P19, SGRF | interleukin 23 subunit alpha |
| BCL9 | LGS | BCL9 transcription coactivator |
| DDX5 | G17P1, HLR1, HUMP68, p68 | DEAD-box helicase 5 |
| BAG1 | BAG-1, HAP, RAP46 | BAG cochaperone 1 |
| CYLD | BRSS, CDMT1, CYLDI, EAC, MFT, M | CYLD lysine 63 deubiquitinase |
| ACTB | BRWS1, PS1TP5BP1 | actin beta |
| KCNMA1 | BKTM, CADEDS, IEG16, KCa1.1, LIW | potassium calcium-activated channel subfamily M alpha 1 |
| PMP22 | CIDP, CMT1A, CMT1E, DSS, GAS-3, | peripheral myelin protein 22 |
| IL7 | IL-7 | interleukin 7 |
| NR1H4 | BAR, FXR, HRR-1, HRR1, PFIC5, RIP1 | nuclear receptor subfamily 1 group H member 4 |
| PLD2 | PLD1C | phospholipase D2 |
| HK2 | HKII, HXK2 | hexokinase 2 |
| KISS1R | AXOR12, CPPB1, GPR54, HH8, HOT | KISS1 receptor |
| MIR150 | MIRN150, miRNA150, mir-150 | microRNA 150 |
| ING4 | my036, p29ING4 | inhibitor of growth family member 4 |
| KITLG | DCUA, DFNA69, FPH2, FPHH, KL-1, | KIT ligand |
| CHUK | IKBKA, IKK-alpha, IKK1, IKKA, NFKB | component of inhibitor of nuclear factor kappa B kinase complex |
| IGF2BP2 | IMP-2, IMP2, VICKZ2 | insulin like growth factor 2 mRNA binding protein 2 |
| DEK | D6S231E | DEK proto-oncogene |
| ARF1 | PVNH8 | ADP ribosylation factor 1 |
| UBE2C | UBCH10, dJ447F3.2 | ubiquitin conjugating enzyme E2 C |
| MIR193A | MIRN193, MIRN193A, mir-193a | microRNA 193a |
| HNRNPK | AUKS, CSBP, HNRPK, TUNP | heterogeneous nuclear ribonucleoprotein K |
| KLK6 | Bssp, Klk7, PRSS18, PRSS9, SP59, h | kallikrein related peptidase 6 |
| HDAC3 | HD3, KDAC3, RPD3, RPD3-2 | histone deacetylase 3 |
| ARRB2 | ARB2, ARR2, BARR2 | arrestin beta 2 |
| IDH2 | D2HGA2, ICD-M, IDH, IDHM, IDP, II | isocitrate dehydrogenase (NADP(+)) 2 |
| STC1 | STC | stanniocalcin 1 |

| | | |
|---|---|---|
| CXCL13 | ANGIE, ANGIE2, BCA-1, BCA1, BLC, | C-X-C motif chemokine ligand 13 |
| PGK1 | HEL-S-68p, MIG10, PGKA | phosphoglycerate kinase 1 |
| NFKB2 | CVID10, H2TF1, LYT-10, LYT10, NF- | nuclear factor kappa B subunit 2 |
| CD9 | BTCC-1, DRAP-27, MIC3, MRP-1, TS | CD9 molecule |
| SRF | MCM1 | serum response factor |
| PTK6 | BRK | protein tyrosine kinase 6 |
| WFDC2 | EDDM4, HE4, WAP5, dJ461P17.6 | WAP four-disulfide core domain 2 |
| PLA2G4A | GURDP, PLA2G4, cPLA2, cPLA2-alp | phospholipase A2 group IVA |
| RORA | IDDECA, NR1F1, ROR1, ROR2, ROR3 | RAR related orphan receptor A |
| ZFP36 | G0S24, GOS24, NUP475, RNF162A, | ZFP36 ring finger protein |
| ISG15 | G1P2, IFI15, IMD38, IP17, UCRP, hU | ISG15 ubiquitin like modifier |
| CA2 | CA-II, CAC, CAII, Car2, HEL-76, HEL- | carbonic anhydrase 2 |
| LGALS9 | HUATA, LGALS9 | galectin 9 |
| JUP | CTNNG, DP3, DPIII, PDGB, PKGB | junction plakoglobin |
| IL7R | CD127, CDW127, IL-7R-alphaA, ILR | interleukin 7 receptor |
| AQP3 | AQP-3, GIL | aquaporin 3 (Gill blood group) |
| CD47 | IAP, MER6, OA3 | CD47 molecule |
| ACTA2 | ACTSA | actin alpha 2, smooth muscle |
| SDHB | CWS2, IP, PGL4, SDH, SDH1, SDH2, | succinate dehydrogenase complex iron sulfur subunit B |
| TJP1 | ZO-1 | tight junction protein 1 |
| SIRT6 | SIR2L6 | sirtuin 6 |
| SLC7A5 | 4F2LC, CD98, D16S469E, E16, LAT1 | solute carrier family 7 member 5 |
| CUL4A | | cullin 4A |
| BTRC | BETA-TRCP, FBW1A, FBXW1, FBXW | beta-transducin repeat containing E3 ubiquitin protein ligase |
| CCL3 | G0S19-1, LD78ALPHA, MIP-1-alpha | C-C motif chemokine ligand 3 |
| PDPK1 | PDK1, PDPK2, PDPK2P, PRO0461 | 3-phosphoinositide dependent protein kinase 1 |
| PRDX2 | HEL-S-2a, NKEF-B, NKEFB, PRP, PRX | peroxiredoxin 2 |
| MIR26B | MIRN26B, hsa-mir-26b, miR-26b | microRNA 26b |
| ETV4 | E1A-F, E1AF, PEA3, PEAS3 | ETS variant transcription factor 4 |
| PSIP1 | DFS70, LEDGF, PAIP, PSIP2, p52, p7 | PC4 and SFRS1 interacting protein 1 |
| NR4A1 | GFRP1, HMR, N10, NAK-1, NGFIB, N | nuclear receptor subfamily 4 group A member 1 |
| MIR132 | MIRN132, miRNA132, mir-132 | microRNA 132 |
| CCN4 | WISP1, WISP1-OT1, WISP1-UT1, W | cellular communication network factor 4 |
| FLI1 | BDPLT21, EWSR2, SIC-1 | Fli-1 proto-oncogene, ETS transcription factor |
| MAP2K4 | JNKK, JNKK1, MAPKK4, MEK4, MKK | mitogen-activated protein kinase kinase 4 |
| TTK | CT96, ESK, MPH1, MPS1, MPS1L1, | TTK protein kinase |
| CTBP1 | BARS, HADDTS | C-terminal binding protein 1 |
| SOD3 | EC-SOD | superoxide dismutase 3 |
| MIR133B | MIRN133B, miRNA133B, mir-133b | microRNA 133b |
| P2RY2 | HP2U, P2RU1, P2U, P2U1, P2UR, P2 | purinergic receptor P2Y2 |
| MIR15A | MIRN15A, hsa-mir-15a, miRNA15A | microRNA 15a |
| YWHAQ | 14-3-3, 1C5, HS1 | tyrosine 3-monooxygenase/tryptophan 5-monooxygenase activa |
| ANXA5 | ANX5, ENX2, HEL-S-7, PP4, RPRGL3 | annexin A5 |
| TEK | CD202B, GLC3E, TIE-2, TIE2, VMCM | TEK receptor tyrosine kinase |
| EPHB4 | CMAVM2, HFASD, HTK, LMPHM7, | EPH receptor B4 |
| CD81 | CVID6, S5.7, TAPA1, TSPAN28 | CD81 molecule |
| NFATC1 | NF-ATC, NF-ATc1.2, NFAT2, NFATc | nuclear factor of activated T cells 1 |
| CLDN7 | CEPTRL2, CLDN-7, CPETRL2, Hs.843 | claudin 7 |
| MIR497 | MIRN497, hsa-mir-497, mir-497 | microRNA 497 |
| ROBO1 | DUTT1, SAX3 | roundabout guidance receptor 1 |
| MX1 | IFI-78K, IFI78, MX, MxA, lncMX1-21 | MX dynamin like GTPase 1 |

| | | |
|---|---|---|
| TNFSF12 | APO3L, DR3LG, TNLG4A, TWEAK | TNF superfamily member 12 |
| ATF2 | CRE-BP1, CREB-2, CREB2, HB16, TR | activating transcription factor 2 |
| UHRF1 | ICBP90, Np95, RNF106, TDRD22, hl | ubiquitin like with PHD and ring finger domains 1 |
| CTSS | | cathepsin S |
| MIR373 | MIRN373, hsa-mir-373, miRNA373 | microRNA 373 |
| MIR224 | MIRN224, miRNA224 | microRNA 224 |
| ATF4 | CREB-2, CREB2, TAXREB67, TXREB | activating transcription factor 4 |
| BAG3 | BAG-3, BIS, CAIR-1, MFM6 | BAG cochaperone 3 |
| YWHAE | 14-3-3E, HEL2, KCIP-1, MDCR, MDS | tyrosine 3-monooxygenase/tryptophan 5-monooxygenase activa |
| GAB2 | | GRB2 associated binding protein 2 |
| PRKD1 | CHDED, PKC-MU, PKCM, PKD, PRK( | protein kinase D1 |
| USP9X | DFFRX, FAF, FAM, MRX99, MRXS99 | ubiquitin specific peptidase 9 X-linked |
| MIR18A | C13orf25, MIR17HG, MIR18, MIRH | microRNA 18a |
| ENPP1 | ARHR2, COLED, M6S1, NPP1, NPPS | ectonucleotide pyrophosphatase/phosphodiesterase 1 |
| FLOT2 | ECS-1, ECS1, ESA, ESA1, M17S1 | flotillin 2 |
| RELN | ETL7, LIS2, PRO1598, RL | reelin |
| CTHRC1 | | collagen triple helix repeat containing 1 |
| COL4A1 | BSVD, BSVD1, PADMAL, RATOR | collagen type IV alpha 1 chain |
| MIR340 | MIRN340, hsa-mir-340, mir-340 | microRNA 340 |
| COL3A1 | EDS4A, EDSVASC, PMGEDSV | collagen type III alpha 1 chain |
| CCL21 | 6Ckine, CKb9, ECL, SCYA21, SLC, T( | C-C motif chemokine ligand 21 |
| IGFBP7 | AGM, FSTL2, IBP-7, IGFBP-7, IGFBP | insulin like growth factor binding protein 7 |
| MAD2L1 | HSMAD2, MAD2 | mitotic arrest deficient 2 like 1 |
| RECQL | RECQL1, RecQ1 | RecQ like helicase |
| LRP6 | ADCAD2, STHAG7 | LDL receptor related protein 6 |
| USP7 | HAUSP, TEF1 | ubiquitin specific peptidase 7 |
| EDNRB | ABCDS, ET-B, ET-BR, ETB, ETB1, ETE | endothelin receptor type B |
| KRT20 | CD20, CK-20, CK20, K20, KRT21 | keratin 20 |
| GRK2 | ADRBK1, BARK1, BETA-ARK1 | G protein-coupled receptor kinase 2 |
| TBX21 | T-PET, T-bet, TBET, TBLYM | T-box transcription factor 21 |
| SCGB2A2 | MGB1, PSBP1, UGB2 | secretoglobin family 2A member 2 |
| LIN28B | CSDD2 | lin-28 homolog B |
| ARHGDIB | D4, GDIA2, GDID4, LYGDI, Ly-GDI, F | Rho GDP dissociation inhibitor beta |
| PTGER4 | EP4, EP4R | prostaglandin E receptor 4 |
| NEDD4 | NEDD4-1, RPF1 | NEDD4 E3 ubiquitin protein ligase |
| SPDEF | PDEF, bA375E1.3 | SAM pointed domain containing ETS transcription factor |
| TRAF4 | CART1, MLN62, RNF83 | TNF receptor associated factor 4 |
| PTH1R | EKNS, PFE, PTHR, PTHR1 | parathyroid hormone 1 receptor |
| HPGD | 15-PGDH, PGDH, PGDH1, PHOAR1, | 15-hydroxyprostaglandin dehydrogenase |
| EDNRA | ET-A, ETA, ETA-R, ETAR, ETRA, MFD | endothelin receptor type A |
| TNFRSF12 | CD266, FN14, TWEAKR | TNF receptor superfamily member 12A |
| AIRE | AIRE1, APECED, APS1, APSI, PGA1 | autoimmune regulator |
| FURIN | FUR, PACE, PCSK3, SPC1 | furin, paired basic amino acid cleaving enzyme |
| AIFM1 | AIF, AUNX1, CMT2D, CMTX4, COW | apoptosis inducing factor mitochondria associated 1 |
| APAF1 | APAF-1, CED4 | apoptotic peptidase activating factor 1 |
| CSK | | C-terminal Src kinase |
| CRK | CRKII, p38 | CRK proto-oncogene, adaptor protein |
| ANG | ALS9, HEL168, RAA1, RNASE4, RNA | angiogenin |
| MIR378A | MIR378, MIRN378, hsa-mir-378, hs | microRNA 378a |
| SLC3A2 | 4F2, 4F2HC, 4T2HC, CD98, CD98HC | solute carrier family 3 member 2 |
| CDC20 | CDC20A, bA276H19.3, p55CDC | cell division cycle 20 |

| | | |
|---|---|---|
| ARRB1 | ARB1, ARR1 | arrestin beta 1 |
| MIR424 | MIR322, MIRN424, hsa-mir-424, m | microRNA 424 |
| CSF3 | C17orf33OS, GCSF, CSF3 | colony stimulating factor 3 |
| USP22 | USP3L | ubiquitin specific peptidase 22 |
| INPPL1 | OPSMD, SHIP2 | inositol polyphosphate phosphatase like 1 |
| MIR34C | MIRN34C, miRNA34C, mir-34c | microRNA 34c |
| ZNF703 | NLZ1, ZEPPO1, ZNF503L, ZPO1 | zinc finger protein 703 |
| FOXL2 | BPES, BPES1, PFRK, PINTO, POF3 | forkhead box L2 |
| MIR23B | MIRN23B, hsa-mir-23b, miRNA23B | microRNA 23b |
| RELB | I-REL, IMD53, IREL, REL-B | RELB proto-oncogene, NF-kB subunit |
| YWHAG | 14-3-3GAMMA, EIEE56, PPP1R170 | tyrosine 3-monooxygenase/tryptophan 5-monooxygenase activa |
| ID2 | GIG8A, ID2H, bHLHb26, ID2 | inhibitor of DNA binding 2 |
| MIR144 | MIRN144, mir-144 | microRNA 144 |
| KRT17 | 39.1, CK-17, K17, PC, PC2, PCHC1 | keratin 17 |
| TBK1 | FTDALS4, IIAE8, NAK, T2K | TANK binding kinase 1 |
| PADI4 | PAD, PAD4, PADI5, PDI4, PDI5 | peptidyl arginine deiminase 4 |
| CXCL5 | ENA-78, SCYB5 | C-X-C motif chemokine ligand 5 |
| HOXA9 | ABD-B, HOX1, HOX1.7, HOX1G | homeobox A9 |
| HMGCR | LDLCQ3 | 3-hydroxy-3-methylglutaryl-CoA reductase |
| MUC2 | MLP, MUC-2, SMUC | mucin 2, oligomeric mucus/gel-forming |
| MIR7-1 | MIRN7-1, hsa-mir-7-1, mir-7-1 | microRNA 7-1 |
| PAX8 | | paired box 8 |
| CTNNA1 | CAP102, MDPT2 | catenin alpha 1 |
| IL24 | C49A, FISP, IL10B, MDA7, MOB5, S | interleukin 24 |
| HDAC4 | AHO3, BDMR, HA6116, HD4, HDAC | histone deacetylase 4 |
| PODXL | Gp200, PC, PCLP, PCLP-1 | podocalyxin like |
| MIR146B | MIRN146B, miRNA146B, mir-146b | microRNA 146b |
| MIR27B | MIR-27b, MIRN27B, miRNA27B | microRNA 27b |
| G3BP1 | G3BP, HDH-VIII | G3BP stress granule assembly factor 1 |
| AGTR2 | AT2, ATGR2, MRX88 | angiotensin II receptor type 2 |
| IRAK1 | IRAK, pelle | interleukin 1 receptor associated kinase 1 |
| AXIN2 | AXIL, ODCRCS | axin 2 |
| EPHB2 | BDPLT22, CAPB, DRT, EK5, EPHT3, | EPH receptor B2 |
| HNRNPA1 | ALS19, ALS20, HNRPA1, HNRPA1L3 | heterogeneous nuclear ribonucleoprotein A1 |
| ARNT | HIF-1-beta, HIF-1beta, HIF1-beta, H | aryl hydrocarbon receptor nuclear translocator |
| GPNMB | HGFIN, NMB, PLCA3 | glycoprotein nmb |
| CUL3 | CUL-3, PHA2E | cullin 3 |
| HSPA9 | CRP40, CSA, EVPLS, GRP-75, GRP75 | heat shock protein family A (Hsp70) member 9 |
| MIR152 | MIRN152, mir-152 | microRNA 152 |
| MALT1 | IMD12, MLT, MLT1, PCASP1 | MALT1 paracaspase |
| IL5 | EDF, IL-5, TRF | interleukin 5 |
| HSP90AB1 | D6S182, HSP84, HSP90B, HSPC2, H | heat shock protein 90 alpha family class B member 1 |
| MIR142 | MIRN142, mir-142 | microRNA 142 |
| GAB1 | DFNB26 | GRB2 associated binding protein 1 |
| MIR124-3 | MIRN124-3, MIRN124A3, mir-124- | microRNA 124-3 |
| GDNF | ATF, ATF1, ATF2, HFB1-GDNF, HSCI | glial cell derived neurotrophic factor |
| PYCARD | ASC, CARD5, TMS, TMS-1, TMS1 | PYD and CARD domain containing |
| WEE1 | WEE1Ahu, WEE1 | WEE1 G2 checkpoint kinase |
| MLLT10 | AF10 | MLLT10 histone lysine methyltransferase DOT1L cofactor |
| SIAH1 | SIAH1A | siah E3 ubiquitin protein ligase 1 |
| MIR494 | MIRN494, hsa-mir-494, mir-494 | microRNA 494 |

| | | |
|---|---|---|
| AXIN1 | AXIN, PPP1R49 | axin 1 |
| CXCR1 | C-C, C-C-CKR-1, CD128, CD181, CD | C-X-C motif chemokine receptor 1 |
| EIF4G1 | EIF-4G1, EIF4F, EIF4G, EIF4GI, P220 | eukaryotic translation initiation factor 4 gamma 1 |
| ADAM9 | CORD9, MCMP, MDC9, Mltng | ADAM metallopeptidase domain 9 |
| RASA1 | CM-AVM, CMAVM, CMAVM1, GAP | RAS p21 protein activator 1 |
| DLX2 | TES-1, TES1 | distal-less homeobox 2 |
| GAS6 | AXLLG, AXSF | growth arrest specific 6 |
| PER2 | FASPS, FASPS1 | period circadian regulator 2 |
| SEMA3A | COLL1, HH16, Hsema-I, Hsema-III, | semaphorin 3A |
| MAPK9 | JNK-55, JNK2, JNK2A, JNK2ALPHA, | mitogen-activated protein kinase 9 |
| TAP1 | ABC17, ABCB2, APT1, D6S114E, PS | transporter 1, ATP binding cassette subfamily B member |
| PDIA3 | ER60, ERp57, ERp60, ERp61, GRP5 | protein disulfide isomerase family A member 3 |
| TSC1 | LAM, TSC | TSC complex subunit 1 |
| CCL4 | ACT2, AT744.1, G-26, HC21, LAG-1, | C-C motif chemokine ligand 4 |
| S1PR1 | CD363, CHEDG1, D1S3362, ECGF1, | sphingosine-1-phosphate receptor 1 |
| CBX5 | HEL25, HP1, HP1A | chromobox 5 |
| SRSF1 | ASF, SF2, SF2p33, SFRS1, SRp30a | serine and arginine rich splicing factor 1 |
| BIRC3 | AIP1, API2, CIAP2, HAIP1, HIAP1, IA | baculoviral IAP repeat containing 3 |
| IRF4 | LSIRF, MUM1, NF-EM5, SHEP8 | interferon regulatory factor 4 |
| AKR1B10 | AKR1B11, AKR1B12, ALDRLn, ARL-1 | aldo-keto reductase family 1 member B10 |
| GATA6 | | GATA binding protein 6 |
| GRB7 | | growth factor receptor bound protein 7 |
| CLDN4 | CPE-R, CPER, CPETR, CPETR1, WBS | claudin 4 |
| OCLN | BLCPMG, PPP1R115, PTORCH1 | occludin |
| HMMR | CD168, IHABP, RHAMM | hyaluronan mediated motility receptor |
| SIRT2 | SIR2, SIR2L, SIR2L2 | sirtuin 2 |
| DROSHA | ETOHI2, HSA242976, RANSE3L, RN | drosha ribonuclease III |
| PCBP1 | HEL-S-85, HNRPE1, HNRPX, hnRNP | poly(rC) binding protein 1 |
| LIF | CDF, DIA, HILDA, MLPLI | LIF interleukin 6 family cytokine |
| TBX3 | TBX3-ISO, UMS, XHL | T-box transcription factor 3 |
| MIR181A1 | MIR213, MIRN181A1, MIRN213, hs | microRNA 181a-1 |
| SRA1 | SRA, SRAP, STRAA1, pp7684 | steroid receptor RNA activator 1 |
| BACH1 | BACH-1, BTBD24 | BTB domain and CNC homolog 1 |
| BIN1 | AMPH2, AMPHL, CNM2, SH3P9 | bridging integrator 1 |
| ADAM33 | C20orf153, DJ964F7.1 | ADAM metallopeptidase domain 33 |
| PIP | GCDFP-15, GCDFP15, GPIP4 | prolactin induced protein |
| CBLB | Cbl-b, Nbla00127, RNF56 | Cbl proto-oncogene B |
| RRM1 | R1, RIR1, RR1 | ribonucleotide reductase catalytic subunit M1 |
| TK1 | TK2 | thymidine kinase 1 |
| GLG1 | CFR-1, ESL-1, MG-160, MG160 | golgi glycoprotein 1 |
| LIMK1 | LIMK, LIMK-1 | LIM domain kinase 1 |
| LAMTOR5 | HBXIP, XIP | late endosomal/lysosomal adaptor, MAPK and MTOR activator 5 |
| BNIP3 | NIP3 | BCL2 interacting protein 3 |
| DACH1 | DACH | dachshund family transcription factor 1 |
| MBD2 | DMTase, NY-CO-41 | methyl-CpG binding domain protein 2 |
| MIR130A | MIRN130A, miRNA130A, mir-130a | microRNA 130a |
| ACKR1 | CCBP1, CD234, DARC, DARC/ACKR | atypical chemokine receptor 1 (Duffy blood group) |
| IL17F | CANDF6, IL-17F, ML-1, ML1 | interleukin 17F |
| CEACAM6 | CD66c, CEAL, NCA | CEA cell adhesion molecule 6 |
| KMT5A | PR-Set7, PR/SET07, SET07, SET8, S | lysine methyltransferase 5A |
| PTN | HARP, HB-GAM, HBBM, HBGF-8, H | pleiotrophin |

| | | |
|---|---|---|
| ENAH | ENA, MENA, NDPP1 | ENAH actin regulator |
| ABCC4 | MOAT-B, MOATB, MRP4 | ATP binding cassette subfamily C member 4 |
| ECM1 | URBWD | extracellular matrix protein 1 |
| MIR34B | MIRN34B, miRNA34B, mir-34b | microRNA 34b |
| MUC5B | MG1, MUC-5B, MUC5, MUC9 | mucin 5B, oligomeric mucus/gel-forming |
| SF3B1 | Hsh155, MDS, PRP10, PRPF10, SAP | splicing factor 3b subunit 1 |
| KRT14 | CK14, EBS3, EBS4, K14, NFJ | keratin 14 |
| LAMP2 | CD107b, DND, LAMP-2, LAMPB, LG | lysosomal associated membrane protein 2 |
| ADAMTS1 | C3-C5, METH1 | ADAM metallopeptidase with thrombospondin type 1 motif 1 |
| ING1 | p24ING1c, p33, p33ING1, p33ING1 | inhibitor of growth family member 1 |
| YES1 | HsT441, P61-YES, Yes, c-yes | YES proto-oncogene 1, Src family tyrosine kinase |
| PSME3 | HEL-S-283, Ki, PA28-gamma, PA28 | proteasome activator subunit 3 |
| NAA10 | ARD1, ARD1A, ARD1P, DXS707, M | N-alpha-acetyltransferase 10, NatA catalytic subunit |
| DIRAS3 | ARHI, NOEY2 | DIRAS family GTPase 3 |
| RBBP8 | COM1, CTIP, JWDS, RIM, SAE2, SC | RB binding protein 8, endonuclease |
| CRKL | | CRK like proto-oncogene, adaptor protein |
| MIR1-1 | MIRN1-1, hsa-mir-1-1, miRNA1-1, | microRNA 1-1 |
| CXADR | CAR, CAR4/6, HCAR | CXADR Ig-like cell adhesion molecule |
| INHA | | inhibin subunit alpha |
| ZNF217 | ZABC1 | zinc finger protein 217 |
| ENO2 | HEL-S-279, NSE | enolase 2 |
| HTATIP2 | CC3, SDR44U1, TIP30 | HIV-1 Tat interactive protein 2 |
| MED1 | CRSP1, CRSP200, DRIP205, DRIP23 | mediator complex subunit 1 |
| DMBT1 | GP340, SAG, SALSA, muclin | deleted in malignant brain tumors 1 |
| DOCK4 | | dedicator of cytokinesis 4 |
| TPM1 | C15orf13, CMD1Y, CMH3, HEL-S-2 | tropomyosin 1 |
| SREBF2 | SREBP-2, SREBP2, bHLHd2 | sterol regulatory element binding transcription factor 2 |
| IRF7 | IMD39, IRF-7, IRF-7HA, IRF7B, IRF7 | interferon regulatory factor 7 |
| LYVE1 | CRSBP-1, HAR, LYVE-1, XLKD1 | lymphatic vessel endothelial hyaluronan receptor 1 |
| TRIM25 | EFP, RNF147, Z147, ZNF147 | tripartite motif containing 25 |
| RRM2 | C2orf48, R2, RR2, RR2M | ribonucleotide reductase regulatory subunit M2 |
| DHFR | DHFRP1, DYR | dihydrofolate reductase |
| NTRK3 | GP145-TrkC, TRKC, gp145(trkC) | neurotrophic receptor tyrosine kinase 3 |
| CD63 | LAMP-3, ME491, MLA1, OMA81H, | CD63 molecule |
| ULK1 | ATG1, ATG1A, UNC51, Unc51.1, hA | unc-51 like autophagy activating kinase 1 |
| PLCE1 | NPHS3, PLCE, PPLC | phospholipase C epsilon 1 |
| RNASEL | PRCA1, RNS4 | ribonuclease L |
| MIR23A | MIRN23A, hsa-mir-23a, miRNA23A | microRNA 23a |
| FUT4 | CD15, ELFT, FCT3A, FUC-TIV, FUTIV | fucosyltransferase 4 |
| ROR1 | NTRKR1, dJ537F10.1 | receptor tyrosine kinase like orphan receptor 1 |
| HIPK2 | PRO0593 | homeodomain interacting protein kinase 2 |
| FLOT1 | | flotillin 1 |
| MIR338 | MIRN338, hsa-mir-338, mir-338 | microRNA 338 |
| SRSF2 | PR264, SC-35, SC35, SFRS2, SFRS2A | serine and arginine rich splicing factor 2 |
| PRDM1 | BLIMP1, PRDI-BF1 | PR/SET domain 1 |
| PLD1 | CVDD | phospholipase D1 |
| WASF3 | Brush-1, SCAR3, WAVE3 | WASP family member 3 |
| MGP | GIG36, MGLAP, NTI | matrix Gla protein |
| PDGFA | PDGF-A, PDGF1 | platelet derived growth factor subunit A |
| FABP3 | FABP11, H-FABP, M-FABP, MDGI, C | fatty acid binding protein 3 |
| COMP | EDM1, EPD1, MED, PSACH, THBS5, | cartilage oligomeric matrix protein |

| | | |
|---|---|---|
| PVR | CD155, HVED, NECL5, Necl-5, PVS, | PVR cell adhesion molecule |
| IL13RA2 | CD213A2, CT19, IL-13R, IL13BP | interleukin 13 receptor subunit alpha 2 |
| PARD3 | ASIP, Baz, PAR3, PAR3alpha, PARD- | par-3 family cell polarity regulator |
| MIR212 | MIRN212, mir-212 | microRNA 212 |
| SEMA4D | A8, BB18, C9orf164, CD100, COLL4 | semaphorin 4D |
| FABP1 | FABPL, L-FABP | fatty acid binding protein 1 |
| PTPN13 | FAP-1, PNP1, PTP-BAS, PTP-BL, PTP | protein tyrosine phosphatase non-receptor type 13 |
| C5AR1 | C5A, C5AR, C5R1, CD88 | complement C5a receptor 1 |
| PIWIL1 | CT80.1, HIWI, MIWI, PIWI | piwi like RNA-mediated gene silencing 1 |
| E2F3 | E2F-3 | E2F transcription factor 3 |
| NR4A2 | HZF-3, NOT, NURR1, RNR1, TINUR | nuclear receptor subfamily 4 group A member 2 |
| PTPN2 | PTN2, PTPT, TC-PTP, TCELLPTP, TCI | protein tyrosine phosphatase non-receptor type 2 |
| WIF1 | WIF-1 | WNT inhibitory factor 1 |
| GDF2 | BMP-9, BMP9, HHT5 | growth differentiation factor 2 |
| DAB2 | DOC-2, DOC2 | DAB adaptor protein 2 |
| RAPGEF3 | CAMP-GEFI, EPAC, EPAC1, HSU792 | Rap guanine nucleotide exchange factor 3 |
| RAB25 | CATX-8, RAB11C | RAB25, member RAS oncogene family |
| CCL22 | A-152E5.1, ABCD-1, DC/B-CK, MDC | C-C motif chemokine ligand 22 |
| CPT1A | CPT1, CPT1-L, L-CPT1 | carnitine palmitoyltransferase 1A |
| GLUD1 | GDH, GDH1, GLUD | glutamate dehydrogenase 1 |
| KCNH1 | EAG, EAG1, Kv10.1, TMBTS, ZLS1, h | potassium voltage-gated channel subfamily H member 1 |
| SETDB1 | ESET, H3-K9-HMTase4, KG1T, KMT | SET domain bifurcated histone lysine methyltransferase 1 |
| CCDC88C | DAPLE, HKRP2, HYC1, KIAA1509, S( | coiled-coil domain containing 88C |
| CXCL9 | CMK, Humig, MIG, SCYB9, crg-10 | C-X-C motif chemokine ligand 9 |
| FOXP2 | CAGH44, SPCH1, TNRC10 | forkhead box P2 |
| PDCD6IP | AIP1, ALIX, DRIP4, HP95 | programmed cell death 6 interacting protein |
| ETS2 | ETS2IT1 | ETS proto-oncogene 2, transcription factor |
| MIR98 | MIRLET7L, MIRN98, hsa-mir-98, mi | microRNA 98 |
| SET | 2PP2A, I2PP2A, IGAAD, IPP2A2, MI | SET nuclear proto-oncogene |
| KDM5B | CT31, JARID1B, MRT65, PLU-1, PLU | lysine demethylase 5B |
| CTAG1B | CT6.1, CTAG, CTAG1, ESO1, LAGE-2 | cancer/testis antigen 1B |
| PTGES | MGST-IV, MGST1-L1, MGST1L1, MI | prostaglandin E synthase |
| PER1 | PER, RIGUI, hPER | period circadian regulator 1 |
| KLF8 | BKLF3, ZNF741 | Kruppel like factor 8 |
| TLN1 | ILWEQ, TLN, talin-1 | talin 1 |
| CXCL2 | CINC-2a, GRO2, GROb, MGSA-b, M | C-X-C motif chemokine ligand 2 |
| TGFBR3 | BGCAN, betaglycan | transforming growth factor beta receptor 3 |
| CST6 | ECTD15 | cystatin E/M |
| PRAME | CT130, MAPE, OIP-4, OIP4 | preferentially expressed antigen in melanoma |
| ADORA2B | ADORA2 | adenosine A2b receptor |
| S100A6 | 2A9, 5B10, CABP, CACY, PRA, S10A | S100 calcium binding protein A6 |
| TMPRSS4 | CAPH2, MT-SP2, TMPRSS3 | transmembrane serine protease 4 |
| MIR193B | MIRN193B, mir-193b | microRNA 193b |
| SLC16A4 | MCT4, MCT5 | solute carrier family 16 member 4 |
| IL9 | HP40, IL-9, P40 | interleukin 9 |
| COL5A1 | EDSC, EDSCL1 | collagen type V alpha 1 chain |
| CD200 | MOX1, MOX2, MRC, OX-2 | CD200 molecule |
| IGF2BP1 | CRD-BP, CRDBP, IMP-1, IMP1, VICK | insulin like growth factor 2 mRNA binding protein 1 |
| FOXA2 | HNF-3-beta, HNF3B, TCF3B | forkhead box A2 |
| PEA15 | HMAT1, HUMMAT1H, MAT1, MAT | proliferation and apoptosis adaptor protein 15 |
| SDHD | CBT1, CII-4, CWS3, PGL, PGL1, QPs | succinate dehydrogenase complex subunit D |

| | | |
|---|---|---|
| GBP1 | | guanylate binding protein 1 |
| HOTTIP | HOXA-AS6, HOXA13-AS1, NCRNA00 | HOXA distal transcript antisense RNA |
| TRPC1 | HTRP-1, TRP1 | transient receptor potential cation channel subfamily C member |
| SIRT7 | SIR2L7 | sirtuin 7 |
| LIPG | EDL, EL, PRO719 | lipase G, endothelial type |
| SPN | CD43, GALGP, GPL115, LSN | sialophorin |
| MIR10A | MIRN10A, hsa-mir-10a, miRNA10A | microRNA 10a |
| NODAL | HTX5 | nodal growth differentiation factor |
| RHOB | ARH6, ARHB, MST081, MSTP081, R | ras homolog family member B |
| LATS1 | WARTS, wts | large tumor suppressor kinase 1 |
| CLDN2 | | claudin 2 |
| RPS6KA1 | HU-1, MAPKAPK1, MAPKAPK1A, RS | ribosomal protein S6 kinase A1 |
| SATB2 | GLSS | SATB homeobox 2 |
| HOXB7 | HHO.C1, HOX2, HOX2C, Hox-2.3 | homeobox B7 |
| MARCKS | 80K-L, MACS, PKCSL, PRKCSL | myristoylated alanine rich protein kinase C substrate |
| TYK2 | IMD35, JTK1 | tyrosine kinase 2 |
| TP53BP2 | 53BP2, ASPP2, BBP, P53BP2, PPP1R | tumor protein p53 binding protein 2 |
| SMC1A | CDLS2, DXS423E, SB1.8, SMC1, SM | structural maintenance of chromosomes 1A |
| C1QA | | complement C1q A chain |
| GOLPH3 | GOPP1, GPP34, MIDAS, Vps74 | golgi phosphoprotein 3 |
| GSTM3 | GST5, GSTB-3, GTM3, GSTM3 | glutathione S-transferase mu 3 |
| PSAP | GLBA, SAP1, SAP2 | prosaposin |
| FBP1 | FBP | fructose-bisphosphatase 1 |
| MIR125B2 | MIRN125B2, mir-125b-2 | microRNA 125b-2 |
| S100A2 | CAN19, S100L | S100 calcium binding protein A2 |
| PAK2 | PAK65, PAKgamma | p21 (RAC1) activated kinase 2 |
| IL17RB | CRL4, EVI27, IL17BR, IL17RH1 | interleukin 17 receptor B |
| FABP7 | B-FABP, BLBP, FABPB, MRG | fatty acid binding protein 7 |
| BCL11A | BCL11A-L-S, BCL11A-XL, BCL11a-M | BAF chromatin remodeling complex subunit BCL11A |
| TXNRD1 | GRIM-12, TR, TR1, TRXR1, TXNR | thioredoxin reductase 1 |
| UBE2N | HEL-S-71, UBC13, UBCHBEN; UBC1 | ubiquitin conjugating enzyme E2 N |
| ARNTL | BMAL1, BMAL1c, JAP3, MOP3, PAS | aryl hydrocarbon receptor nuclear translocator like |
| SMARCE1 | BAF57, CSS5 | SWI/SNF related, matrix associated, actin dependent regulator c |
| STC2 | STC-2, STCRP | stanniocalcin 2 |
| CSTA | AREI, PSS4, STF1, STFA | cystatin A |
| RORC | IMD42, NR1F3, RORG, RZR-GAMM | RAR related orphan receptor C |
| SLC1A5 | AAAT, ASCT2, ATBO, M7V1, M7VS1 | solute carrier family 1 member 5 |
| CCAT2 | LINC00873, NCCP1 | colon cancer associated transcript 2 |
| RBMS3 | | RNA binding motif single stranded interacting protein 3 |
| LRIG1 | LIG-1, LIG1 | leucine rich repeats and immunoglobulin like domains 1 |
| RICTOR | AVO3, PIA, hAVO3 | RPTOR independent companion of MTOR complex 2 |
| PROX1 | | prospero homeobox 1 |
| PRKAA2 | AMPK, AMPK2, AMPKa2, PRKAA | protein kinase AMP-activated catalytic subunit alpha 2 |
| PBK | CT84, HEL164, Nori-3, SPK, TOPK | PDZ binding kinase |
| SRPK1 | SFRSK1 | SRSF protein kinase 1 |
| VAV2 | VAV-2 | vav guanine nucleotide exchange factor 2 |
| SENP1 | SuPr-2 | SUMO specific peptidase 1 |
| NEDD8 | NEDD-8 | NEDD8 ubiquitin like modifier |
| ZBTB7A | FBI-1, FBI1, LRF, TIP21, ZBTB7, ZNF | zinc finger and BTB domain containing 7A |
| MIR503 | MIRN503, hsa-mir-503, mir-503 | microRNA 503 |
| ATG5 | APG5, APG5-LIKE, APG5L, ASP, SCA | autophagy related 5 |

| | | |
|---|---|---|
| MIR449A | MIRN449, MIRN449A, hsa-mir-449 | microRNA 449a |
| DAB2IP | AF9Q34, AIP-1, AIP1, DIP1/2 | DAB2 interacting protein |
| GMNN | Gem, MGORS6 | geminin DNA replication inhibitor |
| MMP10 | SL-2, STMY2 | matrix metallopeptidase 10 |
| EBAG9 | EB9, PDAF | estrogen receptor binding site associated antigen 9 |
| DDB2 | DDBB, UV-DDB2, XPE | damage specific DNA binding protein 2 |
| GPI | AMF, GNPI, NLK, PGI, PHI, SA-36, S. | glucose-6-phosphate isomerase |
| EGFL7 | NEU1, VE-STATIN, ZNEU1 | EGF like domain multiple 7 |
| SMO | CRJS, FZD11, GxH, SMO | smoothened, frizzled class receptor |
| SULF1 | SULF-1 | sulfatase 1 |
| SEMA3F | SEMA-IV, SEMA4, SEMAK | semaphorin 3F |
| KLK4 | AI2A1, ARM1, EMSP, EMSP1, KLK-L | kallikrein related peptidase 4 |
| TNFAIP8L2 | TIPE2 | TNF alpha induced protein 8 like 2 |
| TMSB4X | FX, PTMB4, TB4X, TMSB4 | thymosin beta 4 X-linked |
| CDK8 | IDDHBA, K35 | cyclin dependent kinase 8 |
| MAP3K14 | FTDCR1B, HS, HSNIK, NIK | mitogen-activated protein kinase kinase kinase 14 |
| TIMELESS | TIM, TIM1, hTIM | timeless circadian regulator |
| TDRD3 | | tudor domain containing 3 |
| STK4 | KRS2, MST1, YSK3 | serine/threonine kinase 4 |
| CLDN3 | C7orf1, CPE-R2, CPETR2, HRVP1, R | claudin 3 |
| PIAS1 | DDXBP1, GBP, GU/RH-II, ZMIZ3 | protein inhibitor of activated STAT 1 |
| PRDX6 | 1-Cys, AOP2, HEL-S-128m, NSGPx, | peroxiredoxin 6 |
| PPP3CA | ACCIID, CALN, CALNA, CALNA1, CC | protein phosphatase 3 catalytic subunit alpha |
| HULC | HCCAT1, LINC00078, NCRNA00078 | hepatocellular carcinoma up-regulated long non-coding RNA |
| RALBP1 | RIP1, RLIP1, RLIP76 | ralA binding protein 1 |
| PREX1 | P-REX1 | phosphatidylinositol-3,4,5-trisphosphate dependent Rac exchan |
| MIR106A | MIRN106A, mir-106, mir-106a | microRNA 106a |
| SMURF1 | | SMAD specific E3 ubiquitin protein ligase 1 |
| MSI1 | | musashi RNA binding protein 1 |
| CSNK1A1 | CK1, CK1a, CKIa, HEL-S-77p, HLCDG | casein kinase 1 alpha 1 |
| LMO2 | LMO-2, RBTN2, RBTNL1, RHOM2, T | LIM domain only 2 |
| CYTOR | C2orf59, LINC00152, NCRNA00152 | cytoskeleton regulator RNA |
| AKAP12 | AKAP250, SSeCKS | A-kinase anchoring protein 12 |
| TRAP1 | HSP 75, HSP75, HSP90L, TRAP-1 | TNF receptor associated protein 1 |
| SFRP2 | FRP-2, SARP1, SDF-5 | secreted frizzled related protein 2 |
| TPT1 | HRF, TCTP, p02, p23 | tumor protein, translationally-controlled 1 |
| MIR101-2 | MIRN101-2, mir-101-2 | microRNA 101-2 |
| FUT3 | CD174, FT3B, FucT-III, LE, Les | fucosyltransferase 3 (Lewis blood group) |
| UGT2B15 | HLUG4, UDPGT 2B8, UDPGT2B15, U | UDP glucuronosyltransferase family 2 member B15 |
| MYO6 | DFNA22, DFNB37 | myosin VI |
| TPX2 | C20orf1, C20orf2, DIL-2, DIL2, FLS3 | TPX2 microtubule nucleation factor |
| WWP1 | AIP5, Tiul1, hSDRP1 | WW domain containing E3 ubiquitin protein ligase 1 |
| DLX4 | BP1, DLX7, DLX8, DLX9, OFC15 | distal-less homeobox 4 |
| MIR129-1 | MIR-129b, MIRN129-1, mir-129-1 | microRNA 129-1 |
| WASL | N-WASP, NWASP, WASPB | WASP like actin nucleation promoting factor |
| TAGLN | SM22, SM22-alpha, SMCC1, WS3-1 | transgelin |
| AMOT | | angiomotin |
| FPR1 | FMLP, FPR | formyl peptide receptor 1 |
| SDC2 | CD362, HSPG, HSPG1, SYND2 | syndecan 2 |
| HAX1 | HCLSBP1, HS1BP1, SCN3 | HCLS1 associated protein X-1 |
| MIR429 | MIRN429, hsa-mir-429, mir-429 | microRNA 429 |

| | | |
|---|---|---|
| HAS2 | | hyaluronan synthase 2 |
| CCAT1 | CARLO5, CARLo-5, onco-lncRNA-40 | colon cancer associated transcript 1 |
| RBBP4 | NURF55, RBAP48, lin-53 | RB binding protein 4, chromatin remodeling factor |
| MIR133A1 | MIRN133A1, mir-133a-1 | microRNA 133a-1 |
| NR2C2 | TAK1, TR4 | nuclear receptor subfamily 2 group C member 2 |
| ASS1 | ASS, CTLN1 | argininosuccinate synthase 1 |
| SND1 | TDRD11, Tudor-SN, p100 | staphylococcal nuclease and tudor domain containing 1 |
| TPD52 | D52, N8L, PC-1, PrLZ, hD52 | tumor protein D52 |
| PGRMC1 | Dap1, HPR6.6, IZA, MPR | progesterone receptor membrane component 1 |
| VAV3 | | vav guanine nucleotide exchange factor 3 |
| SOX17 | VUR3 | SRY-box transcription factor 17 |
| TNK2 | ACK, ACK-1, ACK1, p21cdc42Hs | tyrosine kinase non receptor 2 |
| GNA13 | G13 | G protein subunit alpha 13 |
| TNFRSF4 | ACT35, CD134, IMD16, OX40, TXGP | TNF receptor superfamily member 4 |
| UBD | FAT10, GABBR1-3, UBD | ubiquitin D |
| SMURF2 | | SMAD specific E3 ubiquitin protein ligase 2 |
| MIR185 | MIRN185, miR-185 | microRNA 185 |
| MIR590 | MIRN590, hsa-mir-590, mir-590 | microRNA 590 |
| CASP10 | ALPS2, FLICE2, MCH4 | caspase 10 |
| UGT2B17 | BMND12, UDPGT2B17 | UDP glucuronosyltransferase family 2 member B17 |
| STS | ARSC, ARSC1, ASC, ES, SSDD, XLI | steroid sulfatase |
| ECT2 | ARHGEF31 | epithelial cell transforming 2 |
| MIR19B1 | C13orf25, MIR17HG, MIR19B, MIR | microRNA 19b-1 |
| ITGB6 | AI1H | integrin subunit beta 6 |
| MIR192 | MIRN192, miR-192, miRNA192 | microRNA 192 |
| LATS2 | KPM | large tumor suppressor kinase 2 |
| CSPG4 | HMW-MAA, MCSP, MCSPG, MEL-C | chondroitin sulfate proteoglycan 4 |
| MTNR1A | MEL-1A-R, MT1 | melatonin receptor 1A |
| PTGER3 | EP3, EP3-I, EP3-II, EP3-III, EP3-IV, E | prostaglandin E receptor 3 |
| LTB4R2 | BLT2, BLTR2, JULF2, KPG_004, LTB4 | leukotriene B4 receptor 2 |
| NDC80 | HEC, HEC1, HsHec1, KNTC2, TID3, H | NDC80 kinetochore complex component |
| NFIB | CTF, HMGIC/NFIB, MACID, NF-I/B, | nuclear factor I B |
| GLS | AAD20, CASGID, EIEE71, GAC, GAM | glutaminase |
| SDC4 | SYND4 | syndecan 4 |
| FZD7 | FzE3 | frizzled class receptor 7 |
| TNFRSF14 | ATAR, CD270, HVEA, HVEM, LIGHT | TNF receptor superfamily member 14 |
| IL2RG | CD132, CIDX, IL-2RG, IMD4, P64, SC | interleukin 2 receptor subunit gamma |
| FERMT2 | KIND2, MIG2, PLEKHC1, UNC112, U | fermitin family member 2 |
| RNF8 | hRNF8 | ring finger protein 8 |
| SULF2 | HSULF-2 | sulfatase 2 |
| IHH | BDA1, HHG2 | Indian hedgehog signaling molecule |
| GNL3 | C77032, E2IG3, NNP47, NS | G protein nucleolar 3 |
| MIR15B | MIRN15B, hsa-mir-15b, miR-15b | microRNA 15b |
| EPB41L3 | 4.1B, DAL-1, DAL1 | erythrocyte membrane protein band 4.1 like 3 |
| LGALS8 | Gal-8, PCTA-1, PCTA1, Po66-CBP | galectin 8 |
| MIR99A | MIRN99A, mir-99a | microRNA 99a |
| SERPINB2 | HsT1201, PAI, PAI-2, PAI2, PLANH2 | serpin family B member 2 |
| NTS | NMN-125, NN, NT, NT/N1, NTS | neurotensin |
| DLL1 | DELTA1, DL1, Delta, NEDBAS | delta like canonical Notch ligand 1 |
| SPOP | BTBD32, TEF2 | speckle type BTB/POZ protein |
| TBXT | SAVA, T, TFT | T-box transcription factor T |

| | | |
|---|---|---|
| MIR199A2 | MIR-199-s, MIRN199A2, mir-199a- | microRNA 199a-2 |
| MAPKAPK | MAPKAP-K2, MK-2, MK2 | MAPK activated protein kinase 2 |
| LAMB3 | AI1A, BM600-125KDA, LAM5, LAM | laminin subunit beta 3 |
| TFEB | ALPHATFEB, BHLHE35, TCFEB | transcription factor EB |
| CDC27 | ANAPC3, APC3Hs, D0S1430E, D17S | cell division cycle 27 |
| PINX1 | Gno1, LPTL, LPTS, Pxr1 | PIN2 (TERF1) interacting telomerase inhibitor 1 |
| MIR301A | MIR301, MIRN301, MIRN301A, mir | microRNA 301a |
| MIR302A | MIRN302, MIRN302A, hsa-mir-302 | microRNA 302a |
| RBBP7 | RbAp46 | RB binding protein 7, chromatin remodeling factor |
| CD68 | GP110, LAMP4, SCARD1 | CD68 molecule |
| MFGE8 | BA46, EDIL1, HMFG, HsT19888, MF | milk fat globule EGF and factor V/VIII domain containing |
| CHFR | RNF116, RNF196 | checkpoint with forkhead and ring finger domains |
| ODC1 | ODC | ornithine decarboxylase 1 |
| SPINT2 | DIAR3, HAI-2, HAI2, Kop, PB | serine peptidase inhibitor, Kunitz type 2 |
| NSD2 | KMT3F, KMT3G, MMSET, REIIBP, T | nuclear receptor binding SET domain protein 2 |
| KAT2A | GCN5, GCN5L2, PCAF-b, hGCN5 | lysine acetyltransferase 2A |
| HDAC5 | HD5, NY-CO-9 | histone deacetylase 5 |
| TFAP2B | AP-2B, AP2-B, PDA2 | transcription factor AP-2 beta |
| BCL2L12 | | BCL2 like 12 |
| NECTIN1 | CD111, CLPED1, ED4, HIgR, HV1S, H | nectin cell adhesion molecule 1 |
| CLDN5 | AWAL, BEC1, CPETRL1, TMDVCF, T | claudin 5 |
| CDH11 | CAD11, CDHOB, ESWS, OB, OSF-4 | cadherin 11 |
| MIRLET7B | LET7B, MIRNLET7B, hsa-let-7b, let- | microRNA let-7b |
| RB1CC1 | ATG17, CC1, FIP200, PPP1R131 | RB1 inducible coiled-coil 1 |
| KLK10 | NES1, PRSSL1 | kallikrein related peptidase 10 |
| CCN6 | LIBC, PPAC, PPD, WISP-3, WISP3 | cellular communication network factor 6 |
| ZFAS1 | C20orf199, HSUP1, HSUP2, NCRNA | ZNFX1 antisense RNA 1 |
| HDGF | HMG1L2 | heparin binding growth factor |
| EGLN3 | HIFP4H3, HIFPH3, PHD3 | egl-9 family hypoxia inducible factor 3 |
| LPAR1 | EDG2, GPR26, Gpcr26, LPA1, Mrec | lysophosphatidic acid receptor 1 |
| MAP2K7 | JNKK2, MAPKK7, MEK, MEK 7, MKK | mitogen-activated protein kinase kinase 7 |
| NRF1 | ALPHA-PAL | nuclear respiratory factor 1 |
| RAB1A | RAB1, YPT1 | RAB1A, member RAS oncogene family |
| HRH4 | AXOR35, BG26, GPCR105, GPRv53, | histamine receptor H4 |
| NNMT | | nicotinamide N-methyltransferase |
| KDM6B | JMJD3, NEDCFSA | lysine demethylase 6B |
| VIPR1 | HVR1, II, PACAP-R-2, PACAP-R2, RD | vasoactive intestinal peptide receptor 1 |
| MIR135B | MIRN135B, mir-135b | microRNA 135b |
| ASAH1 | AC, ACDase, ASAH, PHP, PHP32, SM | N-acylsphingosine amidohydrolase 1 |
| CADPS | CADPS1, CAPS, CAPS1, UNC-31 | calcium dependent secretion activator |
| SRSF3 | SFRS3, SRp20 | serine and arginine rich splicing factor 3 |
| CDC25B | | cell division cycle 25B |
| MIR135A1 | MIRN135-1, MIRN135A1, mir-135a | microRNA 135a-1 |
| OLFM4 | GC1, GW112, OLM4, OlfD, UNQ362 | olfactomedin 4 |
| NDUFA13 | B16.6, CDA016, CGI-39, GRIM-19, G | NADH:ubiquinone oxidoreductase subunit A13 |
| EPS8 | DFNB102 | epidermal growth factor receptor pathway substrate 8 |
| DUSP6 | HH19, MKP3, PYST1 | dual specificity phosphatase 6 |
| SERPINE2 | GDN, GDNPF, PI-7, PI7, PN-1, PN1, | serpin family E member 2 |
| HIC1 | ZBTB29, ZNF901, hic-1 | HIC ZBTB transcriptional repressor 1 |
| TRIM29 | ATDC | tripartite motif containing 29 |
| POLI | RAD30B, RAD3OB, eta2 | DNA polymerase iota |

| | | |
|---|---|---|
| SYVN1 | DER3, HRD1 | synoviolin 1 |
| HK1 | HK-ta, HK1-tb, HK1-tc, HKD, HKI, H | hexokinase 1 |
| JUNB | AP-1 | JunB proto-oncogene, AP-1 transcription factor subunit |
| EPHB6 | HEP | EPH receptor B6 |
| IGFBP4 | BP-4, HT29-IGFBP, IBP4, IGFBP-4 | insulin like growth factor binding protein 4 |
| CKS1B | CKS1, PNAS-16, PNAS-18, ckshs1 | CDC28 protein kinase regulatory subunit 1B |
| RACGAP1 | CYK4, HsCYK-4, ID-GAP, MgcRacGA | Rac GTPase activating protein 1 |
| CXCL14 | BMAC, BRAK, KEC, KS1, MIP-2g, MI | C-X-C motif chemokine ligand 14 |
| MIR506 | MIRN506, hsa-mir-506, mir-506 | microRNA 506 |
| PSMB8 | ALDD, D6S216, D6S216E, JMP, LMI | proteasome 20S subunit beta 8 |
| PTK7 | CCK-4, CCK4 | protein tyrosine kinase 7 (inactive) |
| TACC3 | ERIC-1, ERIC1, Tacc4, maskin | transforming acidic coiled-coil containing protein 3 |
| MIR124-2 | MIRN124-2, MIRN124A2, mir-124- | microRNA 124-2 |
| SOCS2 | CIS2, Cish2, SOCS-2, SSI-2, SSI2, ST/ | suppressor of cytokine signaling 2 |
| SETD2 | HBP231, HIF-1, HIP-1, HSPC069, HY | SET domain containing 2, histone lysine methyltransferase |
| FUT8 | CDGF, CDGF1 | fucosyltransferase 8 |
| USP14 | TGT | ubiquitin specific peptidase 14 |
| CRABP2 | CRABP-II, RBP6 | cellular retinoic acid binding protein 2 |
| NECTIN4 | EDSS1, LNIR, PRR4, PVRL4, nectin-4 | nectin cell adhesion molecule 4 |
| PTPRA | HEPTP, HLPR, HPTPA, HPTPalpha, L | protein tyrosine phosphatase receptor type A |
| MAP3K8 | AURA2, COT, EST, ESTF, MEKK8, TP | mitogen-activated protein kinase kinase kinase 8 |
| ZFHX3 | ATBF1, ATBT, C16orf47, ZFH-3, ZNF | zinc finger homeobox 3 |
| CCN5 | CT58, CTGF-L, WISP2 | cellular communication network factor 5 |
| CSE1L | CAS, CSE1, XPO2 | chromosome segregation 1 like |
| JAG2 | HJ2, SER2 | jagged canonical Notch ligand 2 |
| INPP4B | | inositol polyphosphate-4-phosphatase type II B |
| PIK3C3 | VPS34, Vps34, hVps34 | phosphatidylinositol 3-kinase catalytic subunit type 3 |
| MAD1L1 | MAD1, PIG9, TP53I9, TXBP181 | mitotic arrest deficient 1 like 1 |
| FSTL1 | FRP, FSL1, MIR198, OCC-1, OCC1, t | follistatin like 1 |
| PLK4 | MCCRP2, SAK, STK18 | polo like kinase 4 |
| TES | TESSS-2, TES | testin LIM domain protein |
| KDM3A | JHDM2A, JHMD2A, JMJD1, JMJD1A | lysine demethylase 3A |
| LTBP1 | | latent transforming growth factor beta binding protein 1 |
| AFAP1-AS | AFAP1-AS, AFAP1AS | AFAP1 antisense RNA 1 |
| HYAL1 | HYAL-1, LUCA1, MPS9, NAT6 | hyaluronidase 1 |
| GPX4 | GPx-4, GSHPx-4, MCSP, PHGPx, SM | glutathione peroxidase 4 |
| ANGPTL2 | ARP2, HARP | angiopoietin like 2 |
| RAD21 | CDLS4, HR21, HRAD21, MCD1, MG | RAD21 cohesin complex component |
| BTG1 | APRO2 | BTG anti-proliferation factor 1 |
| UIMC1 | RAP80, X2HRIP110 | ubiquitin interaction motif containing 1 |
| MIR217 | MIRN217, mir-217 | microRNA 217 |
| GLUL | GLNS, GS, PIG43, PIG59 | glutamate-ammonia ligase |
| UGCG | GCS, GLCT1 | UDP-glucose ceramide glucosyltransferase |
| RBM3 | IS1-RNPL, RNPL | RNA binding motif protein 3 |
| DIAPH1 | DFNA1, DIA1, DRF1, LFHL1, SCBMS | diaphanous related formin 1 |
| EPHA3 | EK4, ETK, ETK1, HEK, HEK4, TYRO4 | EPH receptor A3 |
| TFF2 | SML1, SP | trefoil factor 2 |
| ITGA9 | ALPHA-RLC, ITGA4L, RLC | integrin subunit alpha 9 |
| NOG | SYM1, SYNS1, SYNS1A | noggin |
| KIF14 | MCPH20, MKS12 | kinesin family member 14 |
| CCN3 | IBP-9, IGFBP-9, IGFBP9, NOV, NOVI | cellular communication network factor 3 |

| | | |
|---|---|---|
| PIK3R2 | MPPH, MPPH1, P85B, p85, p85-BET | phosphoinositide-3-kinase regulatory subunit 2 |
| TGIF1 | HPE4, TGIF | TGFB induced factor homeobox 1 |
| HOXA5 | HOX1, HOX1.3, HOX1C | homeobox A5 |
| CYB5R3 | B5R, DIA1 | cytochrome b5 reductase 3 |
| CUX1 | CASP, CDP, CDP/Cut, CDP1, COY1, ( | cut like homeobox 1 |
| MAPK12 | ERK-6, ERK3, ERK6, MAPK 12, P38G | mitogen-activated protein kinase 12 |
| VLDLR | CAMRQ1, CARMQ1, CHRMQ1, VLD | very low density lipoprotein receptor |
| PANX1 | MRS1, OOMD7, PX1, UNQ2529 | pannexin 1 |
| MIR485 | MIRN485, hsa-mir-485, mir-485 | microRNA 485 |
| MAP3K11 | MEKK11, MLK-3, MLK3, PTK1, SPRK | mitogen-activated protein kinase kinase kinase 11 |
| GNA12 | NNX3, RMP, gep | G protein subunit alpha 12 |
| CA1 | CA-I, CAB, Car1, HEL-S-11 | carbonic anhydrase 1 |
| ID3 | HEIR-1, bHLHb25 | inhibitor of DNA binding 3, HLH protein |
| IBSP | BNSP, BSP, BSP-II, SP-II | integrin binding sialoprotein |
| MIR328 | MIRN328, hsa-mir-328, mir-328 | microRNA 328 |
| KCNN3 | KCa2.3, SK3, SKCA3, ZLS3, hSK3 | potassium calcium-activated channel subfamily N member 3 |
| DNAJB6 | DJ4, DnaJ, HHDJ1, HSJ-2, HSJ2, LGM | DnaJ heat shock protein family (Hsp40) member B6 |
| CTBP2 | | C-terminal binding protein 2 |
| HPN | TMPRSS1 | hepsin |
| SLC2A3 | GLUT3 | solute carrier family 2 member 3 |
| SERPINA5 | PAI-3, PAI3, PCI, PCI-B, PLANH3, PR | serpin family A member 5 |
| PTP4A1 | HH72, PRL-1, PRL1, PTP(CAAX1), PT | protein tyrosine phosphatase 4A1 |
| MEIS1 | | Meis homeobox 1 |
| FER | PPP1R74, TYK3, p94-Fer | FER tyrosine kinase |
| CA12 | CA-XII, CAXII, HsT18816, T18816 | carbonic anhydrase 12 |
| FFAR4 | BMIQ10, GPR120, GPR129, GT01, ( | free fatty acid receptor 4 |
| UTRN | DMDL, DRP, DRP1 | utrophin |
| ANXA7 | ANX7, SNX, SYNEXIN | annexin A7 |
| LUM | LDC, SLRR2D | lumican |
| LGMN | AEP1, PRSC1, LGMN | legumain |
| HOXB9 | HOX-2.5, HOX2, HOX2E | homeobox B9 |
| MIR191 | MIRN191, miR-191 | microRNA 191 |
| ETV1 | ER81 | ETS variant transcription factor 1 |
| APPL1 | APPL, DIP13alpha, MODY14 | adaptor protein, phosphotyrosine interacting with PH domain ar |
| CD2 | LFA-2, SRBC, T11 | CD2 molecule |
| ADAM15 | MDC15 | ADAM metallopeptidase domain 15 |
| SPOCK1 | SPOCK, TESTICAN, TIC1 | SPARC (osteonectin), cwcv and kazal like domains proteoglycan 1 |
| RAC2 | EN-7, Gx, HSPC022, p21-Rac2 | Rac family small GTPase 2 |
| ATE1 | | arginyltransferase 1 |
| TPM2 | AMCD1, DA1, DA2B, DA2B4, HEL-S- | tropomyosin 2 |
| MIR128-1 | MIR128A, MIRN128-1, MIRN128A, | microRNA 128-1 |
| MIR135A2 | MIRN135-2, MIRN135A2, mir-135a | microRNA 135a-2 |
| MSI2 | MSI2H | musashi RNA binding protein 2 |
| LEPQTL1 | LSL | Leptin, serum levels of |
| VASH1 | KIAA1036, TTCP 1 | vasohibin 1 |
| RAD9A | RAD9 | RAD9 checkpoint clamp component A |
| E2F2 | E2F-2 | E2F transcription factor 2 |
| UBR5 | DD5, EDD, EDD1, HYD | ubiquitin protein ligase E3 component n-recognin 5 |
| PPP2R2A | B55A, B55ALPHA, PR52A, PR55A, P | protein phosphatase 2 regulatory subunit Balpha |
| ADGRE5 | CD97, TM7LN1 | adhesion G protein-coupled receptor E5 |
| MIR181B1 | MIRN181B1, mir-181b-1 | microRNA 181b-1 |

| | | |
|---|---|---|
| CHKA | CHK, CK, CKI, EK | choline kinase alpha |
| TP53INP1 | SIP, TP53DINP1A, TP53INP1B, Teap | tumor protein p53 inducible nuclear protein 1 |
| TMPRSS3 | DFNB10, DFNB8, ECHOS1, TADG12 | transmembrane serine protease 3 |
| ERO1A | ERO1-L, ERO1-L-alpha, ERO1-alpha | endoplasmic reticulum oxidoreductase 1 alpha |
| ALDH1A3 | ALDH1A6, ALDH6, MCOP8, RALDH3 | aldehyde dehydrogenase 1 family member A3 |
| FOXF2 | FKHL6, FREAC-2, FREAC2 | forkhead box F2 |
| MAF | AYGRP, CCA4, CTRCT21, c-MAF | MAF bZIP transcription factor |
| TMEM45B | | transmembrane protein 45B |
| KDM5A | RBBP-2, RBBP2, RBP2 | lysine demethylase 5A |
| DVL2 | | dishevelled segment polarity protein 2 |
| IL19 | IL-10C, MDA1, NG.1, ZMDA1 | interleukin 19 |
| S1PR3 | EDG-3, EDG3, LPB3, S1P3 | sphingosine-1-phosphate receptor 3 |
| ANXA4 | ANX4, HEL-S-274, P32.5, PAP-II, PIG | annexin A4 |
| HNRNPM | CEAR4, HNRPM, HNRPM4, HTGR1, | heterogeneous nuclear ribonucleoprotein M |
| TWIST2 | AMS, BBRSAY, DERMO1, FFDD3, SE | twist family bHLH transcription factor 2 |
| COP1 | CFAP78, FAP78, RFWD2, RNF200 | COP1 E3 ubiquitin ligase |
| MIR455 | MIRN455, hsa-mir-455, mir-455 | microRNA 455 |
| MIR9-2 | MIRN9-2, hsa-mir-9-2, miRNA9-2, m | microRNA 9-2 |
| PRSS8 | CAP1, PROSTASIN | serine protease 8 |
| CAPNS1 | CALPAIN4, CANP, CANPS, CAPN4, C | calpain small subunit 1 |
| SSRP1 | FACT, FACT80, T160 | structure specific recognition protein 1 |
| CCL19 | CKb11, ELC, MIP-3b, MIP3B, SCYA1 | C-C motif chemokine ligand 19 |
| SPRY4-IT1 | SPRIGHTLY | SPRY4 intronic transcript 1 |
| HOXA1 | BSAS, HOX1, HOX1F | homeobox A1 |
| IFITM1 | 9-27, CD225, DSPA2a, IFI17, LEU13 | interferon induced transmembrane protein 1 |
| MCM5 | CDC46, MGORS8, P1-CDC46 | minichromosome maintenance complex component 5 |
| RAB27B | C25KG | RAB27B, member RAS oncogene family |
| SMAD6 | AOVD2, HsT17432, MADH6, MADH | SMAD family member 6 |
| HRG | HPRGP, THPH11, HRG | histidine rich glycoprotein |
| SP7 | OI11, OI12, OSX, osterix | Sp7 transcription factor |
| HIP1 | HIP-I, ILWEQ, SHON, SHONbeta, SH | huntingtin interacting protein 1 |
| SKIL | SNO, SnoA, SnoI, SnoN | SKI like proto-oncogene |
| MTAP | BDMF, DMSFH, DMSMFH, HEL-249 | methylthioadenosine phosphorylase |
| MBNL1 | EXP, MBNL | muscleblind like splicing regulator 1 |
| ARHGEF2 | GEF, GEF-H1, GEFH1, LFP40, Lfc, N | Rho/Rac guanine nucleotide exchange factor 2 |
| MIR196A1 | MIRN196-1, MIRN196A1, mir-196a | microRNA 196a-1 |
| HIF1AN | FIH1 | hypoxia inducible factor 1 subunit alpha inhibitor |
| MIR134 | MIRN134, mir-134 | microRNA 134 |
| ADAM8 | CD156, CD156a, MS2 | ADAM metallopeptidase domain 8 |
| SIPA1 | SPA1 | signal-induced proliferation-associated 1 |
| AKR1C2 | AKR1C-pseudo, BABP, DD, DD-2, D | aldo-keto reductase family 1 member C2 |
| SDHA | CMD1GG, FP, PGL5, SDH1, SDH2, S | succinate dehydrogenase complex flavoprotein subunit A |
| EPHA4 | EK8, HEK8, SEK, TYRO1 | EPH receptor A4 |
| MIR199B | MIRN199B, mir-199b | microRNA 199b |
| KDM4C | GASC1, JHDM3C, JMJD2C, TDRD14 | lysine demethylase 4C |
| ST8SIA1 | GD3S, SIAT8, SIAT8-A, SIAT8A, ST8S | ST8 alpha-N-acetyl-neuraminide alpha-2,8-sialyltransferase 1 |
| PSMD9 | Rpn4, p27 | proteasome 26S subunit, non-ATPase 9 |
| ABI1 | ABI-1, ABLBP4, E3B1, NAP1BP, SSH | abl interactor 1 |
| BCL2L2 | BCL-W, BCL2-L-2, BCLW, PPP1R51 | BCL2 like 2 |
| FGF8 | AIGF, FGF-8, HBGF-8, HH6, KAL6 | fibroblast growth factor 8 |
| SPAG9 | CT89, HLC-6, HLC4, HLC6, JIP-4, JIP | sperm associated antigen 9 |

| | | |
|---|---|---|
| MAGEA3 | CT1.3, HIP8, HYPD, MAGE3, MAGE. | MAGE family member A3 |
| HOXA11 | HOX1, HOX1I, RUSAT1 | homeobox A11 |
| TBL1XR1 | C21, DC42, IRA1, MRD41, TBLR1 | TBL1X receptor 1 |
| CHD1L | ALC1, CHDL | chromodomain helicase DNA binding protein 1 like |
| FRZB | FRE, FRITZ, FRP-3-1, FRZB-PEN, FRZ | frizzled related protein |
| CBR1 | CBR, SDR21C1, hCBR1 | carbonyl reductase 1 |
| MIR30C1 | MIRN30C1, mir-30c-1 | microRNA 30c-1 |
| MAL | MVP17, VIP17 | mal, T cell differentiation protein |
| EEF1A2 | EEF1AL, EF-1-alpha-2, EF1A, EIEE33 | eukaryotic translation elongation factor 1 alpha 2 |
| KDM4B | JMJD2B, TDRD14B | lysine demethylase 4B |
| MELK | HPK38 | maternal embryonic leucine zipper kinase |
| MGAT5 | GNT-V, GNT-VAA, glcNAc-T V, MGA | alpha-1,6-mannosylglycoprotein 6-beta-N-acetylglucosaminyltra |
| LAMP3 | CD208, DC LAMP, DC-LAMP, DCLAI | lysosomal associated membrane protein 3 |
| CORO1C | HCRNN4 | coronin 1C |
| MIR138-2 | MIRN138-2, mir-138-2 | microRNA 138-2 |
| EFNB1 | CFND, CFNS, EFB1, EFL3, EPLG2, El | ephrin B1 |
| ABCC5 | ABC33, EST277145, MOAT-C, MOA | ATP binding cassette subfamily C member 5 |
| PIWIL2 | CT80, HILI, PIWIL1L, mili | piwi like RNA-mediated gene silencing 2 |
| PLAC1 | CT92, OOSP2B, OOSP2L | placenta enriched 1 |
| WNT2 | INT1L1, IRP | Wnt family member 2 |
| TRIM33 | ECTO, PTC7, RFG7, TF1G, TIF1G, TI | tripartite motif containing 33 |
| ARHGEF7 | BETA-PIX, COOL-1, COOL1, Nbla10 | Rho guanine nucleotide exchange factor 7 |
| NR2E3 | ESCS, PNR, RNR, RP37, rd7 | nuclear receptor subfamily 2 group E member 3 |
| RAG2 | RAG-2 | recombination activating 2 |
| RBM38 | HSRNASEB, RNPC1, SEB4B, SEB4D, | RNA binding motif protein 38 |
| LAMA1 | LAMA, PTBHS, S-LAM-alpha | laminin subunit alpha 1 |
| TCIRG1 | ATP6N1C, ATP6V0A3, Atp6i, OC-11 | T cell immune regulator 1, ATPase H+ transporting V0 subunit a3 |
| LIMS1 | PINCH, PINCH-1, PINCH1 | LIM zinc finger domain containing 1 |
| MAGEC2 | CT10, HCA587, MAGEE1 | MAGE family member C2 |
| EPB41L5 | BE37, LULU, LULU1, YMO1, YRT | erythrocyte membrane protein band 4.1 like 5 |
| CITED2 | ASD8, MRG-1, MRG1, P35SRJ, VSD | Cbp/p300 interacting transactivator with Glu/Asp rich carboxy-te |
| AKAP9 | AKAP-9, AKAP350, AKAP450, CG-N | A-kinase anchoring protein 9 |
| MIR24-2 | MIRN24-2, miR-24-2, miRNA24-2 | microRNA 24-2 |
| MYO5A | GS1, MYH12, MYO5, MYR12 | myosin VA |
| CAPG | AFCP, HEL-S-66, MCP | capping actin protein, gelsolin like |
| MT-CO2 | COII, MTCO2, COX2 | mitochondrially encoded cytochrome c oxidase II |
| ATG7 | APG7-LIKE, APG7L, GSA7 | autophagy related 7 |
| SRGN | PPG, PRG, PRG1 | serglycin |
| HSPB2 | HSP27, Hs.78846, LOH11CR1K, MK | heat shock protein family B (small) member 2 |
| DOCK1 | DOCK180, ced5 | dedicator of cytokinesis 1 |
| MMP26 | | matrix metallopeptidase 26 |
| CLCN3 | CLC3, ClC-3 | chloride voltage-gated channel 3 |
| TEAD4 | EFTR-2, RTEF1, TCF13L1, TEF-3, TE | TEA domain transcription factor 4 |
| MIR186 | MIRN186, miR-186 | microRNA 186 |
| ARL6IP5 | DERP11, GTRAP3-18, HSPC127, JW | ADP ribosylation factor like GTPase 6 interacting protein 5 |
| CLIP1 | CLIP, CLIP-17070, CYLN1, RSN, CLIP | CAP-Gly domain containing linker protein 1 |
| AFDN | AF6, MLL-AF6, MLLT4, l-afadin | afadin, adherens junction formation factor |
| ECRG4 | C2orf40 | ECRG4 augurin precursor |
| SERPINA4 | KAL, KLST, KST, PI-4, PI4, kallistatin | serpin family A member 4 |
| MIR423 | MIRN423, hsa-mir-423, mir-423 | microRNA 423 |
| MIR130B | MIRN130B, mir-130b | microRNA 130b |

| | | |
|---|---|---|
| SESN2 | HI95, SES2, SEST2 | sestrin 2 |
| ZBTB33 | ZNF-kaiso, ZNF348 | zinc finger and BTB domain containing 33 |
| PLXNB1 | PLEXIN-B1, PLXN5, SEP | plexin B1 |
| MIR30B | MIRN30B, mir-30b | microRNA 30b |
| AHNAK | AHNAKRS, PM227 | AHNAK nucleoprotein |
| AGO1 | EIF2C, EIF2C1, GERP95, Q99, hAgo1 | argonaute RISC component 1 |
| FERMT3 | KIND3, MIG-2, MIG2B, UNC112C, U | fermitin family member 3 |
| BCAM | AU, CD239, LU, MSK19 | basal cell adhesion molecule (Lutheran blood group) |
| MIR9-3 | MIRN9-3, hsa-mir-9-3, miRNA9-3, r | microRNA 9-3 |
| SASH1 | CAPOK, DUH1, SH3D6A, dJ323M4.1 | SAM and SH3 domain containing 1 |
| KLF9 | BTEB, BTEB1 | Kruppel like factor 9 |
| MAZ | PUR1, Pur-1, SAF-1, SAF-2, SAF-3, Z | MYC associated zinc finger protein |
| MIR184 | EDICT, MIRN184, miR-184 | microRNA 184 |
| SCN1B | ATFB13, BRGDA5, EIEE52, GEFSP1 | sodium voltage-gated channel beta subunit 1 |
| PCLAF | KIAA0101, L5, NS5ATP9, OEATC, OI | PCNA clamp associated factor |
| MIAT | C22orf35, GOMAFU, LINC00066, N | myocardial infarction associated transcript |
| BCAT1 | BCATC, BCT1, ECA39, MECA39, PNA | branched chain amino acid transaminase 1 |
| HOPX | CAMEO, HOD, HOP, LAGY, NECC1, | HOP homeobox |
| ANTXR1 | ATR, GAPO, TEM8 | ANTXR cell adhesion molecule 1 |
| HAS1 | HAS | hyaluronan synthase 1 |
| MST1 | D3F15S2, DNF15S2, HGFL, MSP, NF | macrophage stimulating 1 |
| ERP29 | C12orf8, ERp28, ERp31, HEL-S-107, | endoplasmic reticulum protein 29 |
| GRPR | BB2, BB2R | gastrin releasing peptide receptor |
| CNTN1 | F3, GP135, MYPCN | contactin 1 |
| MCU | C10orf42, CCDC109A, HsMCU | mitochondrial calcium uniporter |
| MIR486-1 | MIR486, MIRN486, hsa-mir-486, hs | microRNA 486-1 |
| FOXD3 | AIS1, Genesis, HFH2, VAMAS2 | forkhead box D3 |
| DANCR | AGU2, ANCR, KIAA0114, SNHG13, I | differentiation antagonizing non-protein coding RNA |
| MIR218-2 | MIRN218-2, mir-218-2 | microRNA 218-2 |
| WRAP53 | DKCB3, TCAB1, WDR79 | WD repeat containing antisense to TP53 |
| CD109 | CPAMD7, p180, r150 | CD109 molecule |
| SNHG1 | LINC00057, NCRNA00057, U22HG, | small nucleolar RNA host gene 1 |
| THBS4 | TSP-4, TSP4 | thrombospondin 4 |
| PARVA | CH-ILKBP, MXRA2 | parvin alpha |
| NISCH | I-1, IR1, IRAS, hIRAS | nischarin |
| PLS3 | BMND18, T-plastin | plastin 3 |
| DNAJA3 | HCA57, TID1, hTID-1 | DnaJ heat shock protein family (Hsp40) member A3 |
| LRG1 | HMFT1766, LRG | leucine rich alpha-2-glycoprotein 1 |
| ACTR3 | ARP3 | actin related protein 3 |
| PALLD | CGI-151, CGI151, MYN, PNCA1, SIH | palladin, cytoskeletal associated protein |
| LGR4 | BNMD17, GPR48 | leucine rich repeat containing G protein-coupled receptor 4 |
| MAP2K5 | HsT17454, MAPKK5, MEK5, PRKMK | mitogen-activated protein kinase kinase 5 |
| SLC39A6 | LIV-1, ZIP6 | solute carrier family 39 member 6 |
| STARD13 | ARHGAP37, DLC2, GT650, LINC004 | StAR related lipid transfer domain containing 13 |
| ETV5 | ERM | ETS variant transcription factor 5 |
| MIR409 | MIRN409, hsa-mir-409, mir-409 | microRNA 409 |
| LPAR2 | EDG-4, EDG4, LPA-2, LPA2 | lysophosphatidic acid receptor 2 |
| TBX2 | VETD | T-box transcription factor 2 |
| CLDN16 | HOMG3, PCLN1 | claudin 16 |
| GFPT1 | CMS12, CMSTA1, GFA, GFAT, GFAT | glutamine--fructose-6-phosphate transaminase 1 |
| RAB3C | | RAB3C, member RAS oncogene family |

| | | |
|---|---|---|
| SETD1A | KMT2F, Set1, Set1A | SET domain containing 1A, histone lysine methyltransferase |
| DCDC2 | DCDC2A, DFNB66, NPHP19, NSC, R | doublecortin domain containing 2 |
| MIR342 | MIRN342, hsa-mir-342 | microRNA 342 |
| EMSY | C11orf30, GL002 | EMSY transcriptional repressor, BRCA2 interacting |
| EPHA1 | EPH, EPHT, EPHT1 | EPH receptor A1 |
| PICK1 | PICK, PRKCABP | protein interacting with PRKCA 1 |
| SDHC | CYB560, CYBL, PGL3, QPS1, SDH3 | succinate dehydrogenase complex subunit C |
| STAB1 | CLEVER-1, FEEL-1, FELE-1, FEX1, SC | stabilin 1 |
| ASPH | AAH, BAH, CASQ2BP1, FDLAB, HAA | aspartate beta-hydroxylase |
| SFRP5 | SARP3 | secreted frizzled related protein 5 |
| LETM1 | SLC55A1 | leucine zipper and EF-hand containing transmembrane protein 1 |
| PAK5 | PAK7 | p21 (RAC1) activated kinase 5 |
| MIR20B | MIRN20B, hsa-mir-20b, mir-20b | microRNA 20b |
| OTUB1 | HSPC263, OTB1, OTU1 | OTU deubiquitinase, ubiquitin aldehyde binding 1 |
| GIT1 | | GIT ArfGAP 1 |
| PAQR3 | RKTG | progestin and adipoQ receptor family member 3 |
| MIR30E | MIRN30E, mir-30e | microRNA 30e |
| BRD7 | BP75, CELTIX1, NAG4 | bromodomain containing 7 |
| TRIP10 | CIP4, HSTP, STOT, STP, TRIP-10 | thyroid hormone receptor interactor 10 |
| MYO9B | CELIAC4, MYR5 | myosin IXB |
| CBX4 | NBP16, PC2 | chromobox 4 |
| PIP4K2A | PI5P4KA, PIP5K2A, PIP5KII-alpha, P | phosphatidylinositol-5-phosphate 4-kinase type 2 alpha |
| PMEPA1 | STAG1, TMEPAI | prostate transmembrane protein, androgen induced 1 |
| DEPTOR | DEP.6, DEPDC6 | DEP domain containing MTOR interacting protein |
| PEG10 | EDR, HB-1, MEF3L, Mar2, Mart2, R | paternally expressed 10 |
| FOXF1 | ACDMPV, FKHL5, FREAC1 | forkhead box F1 |
| STK3 | KRS1, MST2 | serine/threonine kinase 3 |
| ABL2 | ABLL, ARG | ABL proto-oncogene 2, non-receptor tyrosine kinase |
| KDM2A | CXXC8, FBL11, FBL7, FBXL11, JHDM | lysine demethylase 2A |
| CASC2 | C10orf5 | cancer susceptibility 2 |
| WASF1 | NEDALVS, SCAR1, WAVE, WAVE1 | WASP family member 1 |
| AKR1C1 | 2-ALPHA-HSD, 20-ALPHA-HSD, C9, | aldo-keto reductase family 1 member C1 |
| MIR382 | MIRN382, hsa-mir-382, mir-382 | microRNA 382 |
| RBM10 | DXS8237E, GPATC9, GPATCH9, S1- | RNA binding motif protein 10 |
| RBP1 | CRABP-I, CRBP, CRBP1, CRBPI, RBP | retinol binding protein 1 |
| MIR381 | MIRN381, hsa-mir-381, mir-381 | microRNA 381 |
| ASAP1 | AMAP1, CENTB4, DDEF1, PAG2, PA | ArfGAP with SH3 domain, ankyrin repeat and PH domain 1 |
| SEMA3C | SEMAE, SemE | semaphorin 3C |
| FXYD5 | DYSAD, HSPC113, IWU1, KCT1, OIT | FXYD domain containing ion transport regulator 5 |
| SOX18 | HLTRS, HLTS | SRY-box transcription factor 18 |
| TNFRSF25 | APO-3, DDR3, DR3, GEF720, LARD, | TNF receptor superfamily member 25 |
| MIR363 | MIR-363, MIRN363, hsa-mir-363 | microRNA 363 |
| PTPN3 | PTP-H1, PTPH1 | protein tyrosine phosphatase non-receptor type 3 |
| G3BP2 | | G3BP stress granule assembly factor 2 |
| VANGL1 | KITENIN, LPP2, STB2, STBM2 | VANGL planar cell polarity protein 1 |
| TIGAR | C12orf5, FR2BP | TP53 induced glycolysis regulatory phosphatase |
| STIM2 | | stromal interaction molecule 2 |
| ATAD2 | ANCCA, CT137, PRO2000 | ATPase family AAA domain containing 2 |
| ITGA7 | | integrin subunit alpha 7 |
| SPRY4 | HH17 | sprouty RTK signaling antagonist 4 |
| MAGEA4 | CT1.4, MAGE-41, MAGE-X2, MAGE | MAGE family member A4 |

| | | |
|---|---|---|
| HVCN1 | HV1, VSOP | hydrogen voltage gated channel 1 |
| MIR370 | MIRN370, hsa-mir-370, mir-370 | microRNA 370 |
| SHARPIN | SIPL1 | SHANK associated RH domain interactor |
| PRSS3 | MTG, PRSS4, T9, TRY3, TRY4 | serine protease 3 |
| SCUBE2 | CEGB1, CEGF1, CEGP1, scube/You | signal peptide, CUB domain and EGF like domain containing 2 |
| TAGLN2 | HA1756 | transgelin 2 |
| KCNJ3 | GIRK1, KGA, KIR3.1 | potassium inwardly rectifying channel subfamily J member 3 |
| MIR129-2 | MIR-129b, MIRN129-2, mir-129-2 | microRNA 129-2 |
| SOX2-OT | NCRNA00043, SOX2OT | SOX2 overlapping transcript |
| LIPH | AH, ARWH2, HYPT7, LAH2, LPDLR, | lipase H |
| ZFX | ZNF926 | zinc finger protein X-linked |
| NSUN2 | MISU, MRT5, SAKI, TRM4 | NOP2/Sun RNA methyltransferase 2 |
| MIR708 | MIRN708, hsa-mir-708 | microRNA 708 |
| MSRA | PMSR | methionine sulfoxide reductase A |
| CYFIP1 | P140SRA-1, SHYC, SRA-1, SRA1 | cytoplasmic FMR1 interacting protein 1 |
| RSF1 | HBXAP, RSF-1, XAP8, p325 | remodeling and spacing factor 1 |
| CACYBP | GIG5, PNAS-107, S100A6BP, SIP | calcyclin binding protein |
| NET1 | ARHGEF8A, NET1 | neuroepithelial cell transforming 1 |
| PKP3 | | plakophilin 3 |
| USP11 | UHX1 | ubiquitin specific peptidase 11 |
| GABRA3 | | gamma-aminobutyric acid type A receptor subunit alpha3 |
| RAC3 | | Rac family small GTPase 3 |
| SRXN1 | C20orf139, Npn3, SRX, SRX1 | sulfiredoxin 1 |
| LIMA1 | EPLIN, LDLCQ8, SREBP3 | LIM domain and actin binding 1 |
| CAP1 | CAP-PEN, CAP1 | cyclase associated actin cytoskeleton regulatory protein 1 |
| EIF5A2 | EIF-5A2, eIF5AII | eukaryotic translation initiation factor 5A2 |
| KIFC1 | HSET, KNSL2 | kinesin family member C1 |
| NMU | | neuromedin U |
| GSDMB | GSDMB-1, GSDML, PP4052, PRO25 | gasdermin B |
| ITIH5 | ITI-HC5, PP14776 | inter-alpha-trypsin inhibitor heavy chain 5 |
| TOB1 | APRO5, APRO6, PIG49, TOB, TROB, | transducer of ERBB2, 1 |
| MAP2K3 | MAPKK3, MEK3, MKK3, PRKMK3, S | mitogen-activated protein kinase kinase 3 |
| MAP4K4 | FLH21957, HEL-S-31, HGK, MEKKK4 | mitogen-activated protein kinase kinase kinase kinase 4 |
| HRH3 | GPCR97, HH3R | histamine receptor H3 |
| ADAMTS9 | | ADAM metallopeptidase with thrombospondin type 1 motif 9 |
| MAML1 | Mam-1, Mam1 | mastermind like transcriptional coactivator 1 |
| MIR16-2 | MIRN16-2, mir-16-2, mir-16-3 | microRNA 16-2 |
| ARPC2 | ARC34, PNAS-139, PRO2446, p34-A | actin related protein 2/3 complex subunit 2 |
| MIR421 | MIRN421, hsa-mir-421 | microRNA 421 |
| GNRH2 | GnRH-II, LH-RHII | gonadotropin releasing hormone 2 |
| PFN2 | D3S1319E, PFL | profilin 2 |
| CCL8 | HC14, MCP-2, MCP2, SCYA10, SCYA | C-C motif chemokine ligand 8 |
| CDK14 | PFTAIRE1, PFTK1 | cyclin dependent kinase 14 |
| RASSF2 | CENP-34, RASFADIN | Ras association domain family member 2 |
| EPHB1 | ELK, EPHT2, Hek6, NET | EPH receptor B1 |
| MXD1 | BHLHC58, MAD, MAD1 | MAX dimerization protein 1 |
| TCF12 | CRS3, HEB, HTF4, HsT17266, TCF-1 | transcription factor 12 |
| C1GALT1 | C1GALT, T-synthase | core 1 synthase, glycoprotein-N-acetylgalactosamine 3-beta-gala |
| SCGB2A1 | LPHC, LPNC, MGB2, UGB3 | secretoglobin family 2A member 1 |
| MAGED1 | DLXIN-1, NRAGE | MAGE family member D1 |
| ING5 | p28ING5 | inhibitor of growth family member 5 |

| | | |
|---|---|---|
| MAS1 | MAS, MGRA | MAS1 proto-oncogene, G protein-coupled receptor |
| CREB3 | LUMAN, LZIP, sLZIP | cAMP responsive element binding protein 3 |
| CCL1 | I-309, P500, SCYA1, SISe, TCA3 | C-C motif chemokine ligand 1 |
| CCNG2 | | cyclin G2 |
| TMEM8B | C9orf127, FP588, LINC00950, NAG- | transmembrane protein 8B |
| SOX7 | | SRY-box transcription factor 7 |
| SOX5 | L-SOX5, L-SOX5B, L-SOX5F, LAMSH | SRY-box transcription factor 5 |
| NMI | | N-myc and STAT interactor |
| TFAP4 | AP-4, bHLHc41 | transcription factor AP-4 |
| MRC2 | CD280, CLEC13E, ENDO180, UPAR/ | mannose receptor C type 2 |
| PRKD2 | HSPC187, PKD2, nPKC-D2 | protein kinase D2 |
| WIPF1 | PRPL-2, WAS2, WASPIP, WIP | WAS/WASL interacting protein family member 1 |
| KLF17 | ZLF393, ZNF393, Zfp393 | Kruppel like factor 17 |
| ELF5 | ESE2 | E74 like ETS transcription factor 5 |
| TMSB10 | MIG12, TB10 | thymosin beta 10 |
| PLA2G10 | GXPLA2, GXSPLA2, SPLA2, sPLA2-X | phospholipase A2 group X |
| DSC3 | CDHF3, DSC, DSC1, DSC2, DSC4, HT | desmocollin 3 |
| MIR675 | MIRN675, hsa-mir-675 | microRNA 675 |
| RIOX2 | JMJD10, MDIG, MINA, MINA53, NC | ribosomal oxygenase 2 |
| EMP1 | CL-20, EMP-1, TMP | epithelial membrane protein 1 |
| MIR216A | MIR216, MIRN216, MIRN216A, mi | microRNA 216a |
| MIR1246 | MIRN1246, hsa-mir-1246 | microRNA 1246 |
| GDF11 | BMP-11, BMP11 | growth differentiation factor 11 |
| RAB22A | | RAB22A, member RAS oncogene family |
| PRDM14 | PFM11 | PR/SET domain 14 |
| ARHGEF1 | GEF1, IMD62, LBCL2, LSC, P115-RH | Rho guanine nucleotide exchange factor 1 |
| UBE2V1 | CIR1, CROC-1, CROC1, UBE2V, UEV | ubiquitin conjugating enzyme E2 V1 |
| NID1 | NID | nidogen 1 |
| PRRX1 | AGOTC, PHOX1, PMX1, PRX-1, PRX | paired related homeobox 1 |
| FOXK1 | FOXK1L | forkhead box K1 |
| LIMK2 | | LIM domain kinase 2 |
| CTSH | ACC-4, ACC-5, ACC4, ACC5, CPSB | cathepsin H |
| MIR495 | MIRN495, hsa-mir-495, mir-495 | microRNA 495 |
| MAPK13 | MAPK 13, MAPK-13, PRKM13, SAP | mitogen-activated protein kinase 13 |
| MFAP5 | AAT9, MAGP-2, MAGP2, MFAP-5, M | microfibril associated protein 5 |
| MAPKAP1 | JC310, MIP1, SIN1, SIN1b, SIN1g | MAPK associated protein 1 |
| PAG1 | CBP, PAG | phosphoprotein membrane anchor with glycosphingolipid micro |
| MIR181A2 | MIRN181A, MIRN181A2, hsa-mir-1 | microRNA 181a-2 |
| RAB1B | | RAB1B, member RAS oncogene family |
| KLF15 | KKLF | Kruppel like factor 15 |
| IFI6 | 6-16, FAM14C, G1P3, IFI-6-1616, IF | interferon alpha inducible protein 6 |
| NUS1 | C6orf68, CDG1AA, MGC:7199, MRI | NUS1 dehydrodolichyl diphosphate synthase subunit |
| EYA2 | EAB1 | EYA transcriptional coactivator and phosphatase 2 |
| PDIA6 | ERP5, P5, TXNDC7 | protein disulfide isomerase family A member 6 |
| DUSP3 | VHR | dual specificity phosphatase 3 |
| MIR127 | MIRN127, miRNA127, mir-127 | microRNA 127 |
| TRIM16 | EBBP | tripartite motif containing 16 |
| HOXA11-A | HOXA-AS51, HOXA11AS, HOXA11S, | HOXA11 antisense RNA |
| HPSE2 | HPA2, HPR2, UFS, UFS1 | heparanase 2 (inactive) |
| PARD6A | PAR-6A, PAR6, PAR6C, PAR6alpha, | par-6 family cell polarity regulator alpha |
| HOXB5 | HHO.C10, HOX2, HOX2A, HU-1, Ho | homeobox B5 |

| | | |
|---|---|---|
| ZNF350 | ZBRK1, ZFQR | zinc finger protein 350 |
| MZF1 | MZF-1B, ZFP98, ZNF42, ZSCAN6, M | myeloid zinc finger 1 |
| SMYD2 | HSKM-B, KMT3C, ZMYND14 | SET and MYND domain containing 2 |
| SENP2 | AXAM2, SMT3IP2 | SUMO specific peptidase 2 |
| GPRC5A | GPCR5A, PEIG-1, RAI3, RAIG1, TIG1 | G protein-coupled receptor class C group 5 member A |
| ELK3 | ERP, NET, SAP-2, SAP2 | ETS transcription factor ELK3 |
| NR2F1 | BBOAS, BBSOAS, COUP-TFI, COUPT | nuclear receptor subfamily 2 group F member 1 |
| E2F7 | | E2F transcription factor 7 |
| GRM4 | GPRC1D, MGLUR4, mGlu4 | glutamate metabotropic receptor 4 |
| KIF18A | MS-KIF18A, PPP1R99 | kinesin family member 18A |
| ACKR2 | CCBP2, CCR10, CCR9, CMKBR9, D6, | atypical chemokine receptor 2 |
| METTL3 | IME4, M6A, MT-A70, Spo8, hMETT | methyltransferase like 3 |
| MYO10 | | myosin X |
| CD200R1 | CD200R, HCRTR2, MOX2R, OX2R | CD200 receptor 1 |
| MMP17 | MMP-17, MT4-MMP, MT4MMP, M | matrix metallopeptidase 17 |
| MIR491 | MIRN491, hsa-mir-491, mir-491 | microRNA 491 |
| PRPF4B | PR4H, PRP4, PRP4H, PRP4K, dJ101 | pre-mRNA processing factor 4B |
| CHL1 | CALL, L1CAM2 | cell adhesion molecule L1 like |
| MIRLET7I | LET7I, MIRNLET7I, hsa-let-7i, let-7i | microRNA let-7i |
| IL13RA1 | CD213A1, CT19, IL-13Ra, NR4 | interleukin 13 receptor subunit alpha 1 |
| MIR490 | MIRN490, hsa-mir-490, miR-490 | microRNA 490 |
| CDCA8 | BOR, BOREALIN, DasraB, MESRGP | cell division cycle associated 8 |
| SERBP1 | CGI-55, CHD3IP, HABP4L, PAI-RBP1 | SERPINE1 mRNA binding protein 1 |
| PIK3R3 | p55, p55-GAMMA, p55PIK | phosphoinositide-3-kinase regulatory subunit 3 |
| EFEMP2 | ARCL1B, FBLN4, MBP1, UPH1 | EGF containing fibulin extracellular matrix protein 2 |
| PPP1R9B | PPP1R6, PPP1R9, SPINO, Spn | protein phosphatase 1 regulatory subunit 9B |
| MNAT1 | CAP35, MAT1, RNF66, TFB3 | MNAT1 component of CDK activating kinase |
| ATG12 | APG12, APG12L, FBR93, HAPG12 | autophagy related 12 |
| RAB11FIP1 | NOEL1A, RCP, rab11-FIP1 | RAB11 family interacting protein 1 |
| RSU1 | RSP-1 | Ras suppressor protein 1 |
| MIR190A | MIR190, MIRN190, hsa-mir-190a, r | microRNA 190a |
| ESRP1 | DFNB109, RBM35A, RMB35A | epithelial splicing regulatory protein 1 |
| CST1 | | cystatin SN |
| CBX7 | | chromobox 7 |
| RBFOX2 | FOX2, Fox-2, HNRBP2, HRNBP2, RB | RNA binding fox-1 homolog 2 |
| MIR542 | MIRN542, hsa-mir-542, mir-542 | microRNA 542 |
| TINCR | LINC00036, NCRNA00036, PLAC2, c | TINCR ubiquitin domain containing |
| MIR489 | MIRN489, hsa-mir-489, mir-489 | microRNA 489 |
| MYOF | FER1L3 | myoferlin |
| MAT2A | MATA2, MATII, SAMS2 | methionine adenosyltransferase 2A |
| TUSC3 | D8S1992, M33, MRT22, MRT7, Ma | tumor suppressor candidate 3 |
| SEMA7A | CD108, CDw108, H-SEMA-K1, H-Se | semaphorin 7A (John Milton Hagen blood group) |
| RBM4 | LARKA, ZCCHC21, ZCRB3A, RBM4 | RNA binding motif protein 4 |
| PCDH17 | PCDH68, PCH68 | protocadherin 17 |
| TET3 | BEFAHRS, hCG_40738 | tet methylcytosine dioxygenase 3 |
| MIRLET7G | LET7G, MIRNLET7G, hsa-let-7g, let- | microRNA let-7g |
| NUAK1 | ARK5 | NUAK family kinase 1 |
| TIE1 | JTK14, TIE | tyrosine kinase with immunoglobulin like and EGF like domains 1 |
| MMP28 | EPILYSIN, MM28, MMP-25, MMP-2 | matrix metallopeptidase 28 |
| RASAL2 | NGAP | RAS protein activator like 2 |
| SEMA3B | LUCA-1, SEMA5, SEMAA, SemA, se | semaphorin 3B |

| | | |
|---|---|---|
| ME1 | HUMNDME, MES | malic enzyme 1 |
| SCGB3A1 | HIN-1, HIN1, LU105, PnSP-2, UGRP | secretoglobin family 3A member 1 |
| NUCKS1 | JC7, NUCKS | nuclear casein kinase and cyclin dependent kinase substrate 1 |
| P4HA1 | P4HA | prolyl 4-hydroxylase subunit alpha 1 |
| RRP1B | KIAA0179, NNP1L, Nnp1, PPP1R13 | ribosomal RNA processing 1B |
| MIR103A1 | MIR103-1, MIRN103-1, mir-103a-1 | microRNA 103a-1 |
| MIR187 | MIRN187, miR-187, miRNA187 | microRNA 187 |
| CXCL3 | CINC-2b, GRO3, GROg, MIP-2b, MI | C-X-C motif chemokine ligand 3 |
| PSMD14 | PAD1, POH1, RPN11 | proteasome 26S subunit, non-ATPase 14 |
| ELF1 | EFTUD1, RIA1 | E74 like ETS transcription factor 1 |
| FGF18 | FGF-18, ZFGF5 | fibroblast growth factor 18 |
| CEACAM3 | CD66D, CEA, CGM1, W264, W282 | CEA cell adhesion molecule 3 |
| RABEP1 | RAB5EP, RABPT5 | rabaptin, RAB GTPase binding effector protein 1 |
| STYK1 | NOK, SuRTK106 | serine/threonine/tyrosine kinase 1 |
| FOSL2 | FRA2 | FOS like 2, AP-1 transcription factor subunit |
| RAB23 | HSPC137 | RAB23, member RAS oncogene family |
| ST3GAL1 | Gal-NAc6S, SIAT4A, SIATFL, ST3Gal | ST3 beta-galactoside alpha-2,3-sialyltransferase 1 |
| TPM4 | HEL-S-108 | tropomyosin 4 |
| FBXO11 | FBX11, IDDFBA, PRMT9, UBR6, UG | F-box protein 11 |
| SIX3 | HPE2 | SIX homeobox 3 |
| PRKD3 | EPK2, PKC-NU, PKD3, PRKCN, nPKC | protein kinase D3 |
| SORBS1 | CAP, FLAF2, R85FL, SH3D5, SH3P12 | sorbin and SH3 domain containing 1 |
| BRAP | BRAP2, IMP, RNF52 | BRCA1 associated protein |
| MACROD1 | LRP16 | mono-ADP ribosylhydrolase 1 |
| PEBP4 | CORK-1, CORK1, GWTM1933, HEL- | phosphatidylethanolamine binding protein 4 |
| E2F6 | E2F-6 | E2F transcription factor 6 |
| GCNT1 | C2GNT, C2GNT-L, C2GNT1, G6NT, | glucosaminyl (N-acetyl) transferase 1 |
| KLK13 | KLK-L4, KLKL4 | kallikrein related peptidase 13 |
| TP73-AS1 | KIAA0495, PDAM | TP73 antisense RNA 1 |
| MIEN1 | C17orf37, C35, ORB3, RDX12, XTP4 | migration and invasion enhancer 1 |
| WNT11 | HWNT11 | Wnt family member 11 |
| USP39 | 65K, CGI-21, HSPC332, SAD1, SNRN | ubiquitin specific peptidase 39 |
| IL17RD | HH18, IL-17RD, IL17RLM, SEF | interleukin 17 receptor D |
| PPFIA1 | LIP.1, LIP1, LIPRIN | PTPRF interacting protein alpha 1 |
| MIR361 | MIRN361, hsa-mir-361, mir-361 | microRNA 361 |
| MIR374A | MIRN374, MIRN374A, hsa-mir-374 | microRNA 374a |
| EPHA7 | EHK-3, EHK3, EK11, HEK11 | EPH receptor A7 |
| SOX1 | | SRY-box transcription factor 1 |
| MIR613 | MIRN613, hsa-mir-613 | microRNA 613 |
| MCM10 | CNA43, DNA43, PRO2249 | minichromosome maintenance 10 replication initiation factor |
| PRMT7 | SBIDDS | protein arginine methyltransferase 7 |
| CPEB1 | CPE-BP1, CPEB, CPEB-1, h-CPEB, h | cytoplasmic polyadenylation element binding protein 1 |
| PHLDA2 | BRW1C, BWR1C, HLDA2, IPL, TSSC | pleckstrin homology like domain family A member 2 |
| CARD10 | BIMP1, CARMA3 | caspase recruitment domain family member 10 |
| ACVR1C | ACVRLK7, ALK7 | activin A receptor type 1C |
| LARP7 | ALAZS, HDCMA18P, PIP7S | La ribonucleoprotein 7, transcriptional regulator |
| MIR365A | MIR365-1, MIRN365-1, hsa-mir-36 | microRNA 365a |
| EIF4A2 | BM-010, DDX2B, EIF4A, EIF4F, eIF- | eukaryotic translation initiation factor 4A2 |
| RABGEF1 | RABEX5, RAP1, rabex-5 | RAB guanine nucleotide exchange factor 1 |
| HOXD3 | HOX1D, HOX4, HOX4A, Hox-4.1 | homeobox D3 |
| MIR33B | MIRN33B, hsa-mir-33b, mir-33b | microRNA 33b |

| | | |
|---|---|---|
| SMUG1 | FDG, HMUDG, UNG3 | single-strand-selective monofunctional uracil-DNA glycosylase 1 |
| RTRAF | C14orf166, CGI-99, CGI99, CLE, CLE | RNA transcription, translation and transport factor |
| SNHG6 | HBII-276HG, NCRNA00058, U87HG | small nucleolar RNA host gene 6 |
| OPCML | IGLON1, OBCAM, OPCM | opioid binding protein/cell adhesion molecule like |
| MIR454 | MIRN454, hsa-mir-454, mir-454 | microRNA 454 |
| CCNB2 | HsT17299 | cyclin B2 |
| IFIT2 | G10P2, GARG-39, IFI-54, IFI-54K, IF | interferon induced protein with tetratricopeptide repeats 2 |
| RGS16 | A28-RGS14, A28-RGS14P, RGS-R | regulator of G protein signaling 16 |
| CBR3 | HEL-S-25, SDR21C2, hCBR3 | carbonyl reductase 3 |
| DNAJB4 | DNAJW, DjB4, HLJ1 | DnaJ heat shock protein family (Hsp40) member B4 |
| RPS6KA2 | HU-2, MAPKAPK1C, RSK, RSK3, S6K | ribosomal protein S6 kinase A2 |
| FOXN3 | C14orf116, CHES1, PRO1635 | forkhead box N3 |
| MIR425 | MIRN425, hsa-mir-425, mir-425 | microRNA 425 |
| EPX | EPO, EPP-PEN, EPXD, EPX | eosinophil peroxidase |
| MIR153-1 | MIRN153-1, mir-153-1 | microRNA 153-1 |
| MIR384 | MIRN384, hsa-mir-384 | microRNA 384 |
| MIR219A1 | MIR219-1, MIRN219-1, MRI219-1, | microRNA 219a-1 |
| CYC1 | MC3DN6, UQCR4 | cytochrome c1 |
| CLCA2 | CACC, CACC3, CLCRG2, CaCC-3 | chloride channel accessory 2 |
| MIR136 | MIRN136, miRNA136, mir-136 | microRNA 136 |
| IQSEC1 | ARF-GEP100, ARFGEP100, BRAG2, | IQ motif and Sec7 domain ArfGEF 1 |
| MAGEC1 | CT7, CT7.1 | MAGE family member C1 |
| MIR630 | MIRN630, hsa-mir-630 | microRNA 630 |
| MASTL | GREATWALL, GW, GWL, MAST-L, T | microtubule associated serine/threonine kinase like |
| CREB3L1 | OASIS, OI16 | cAMP responsive element binding protein 3 like 1 |
| TAB3 | MAP3K7IP3, NAP1 | TGF-beta activated kinase 1 (MAP3K7) binding protein 3 |
| CDK3 | | cyclin dependent kinase 3 |
| PTENP1 | PTEN-rs, PTEN2, PTENpg1, PTH2, p | phosphatase and tensin homolog pseudogene 1 |
| MIR519D | MIRN519D, mir-519d | microRNA 519d |
| AFAP1L2 | CTB-1144G6.4, KIAA1914, XB130 | actin filament associated protein 1 like 2 |
| BCYRN1 | BC200, BC200a, LINC00004, NCRN/ | brain cytoplasmic RNA 1 |
| PEAK1 | SGK269 | pseudopodium enriched atypical kinase 1 |
| CPEB4 | CPE-BP4, hCPEB-4 | cytoplasmic polyadenylation element binding protein 4 |
| DACT2 | C6orf116, DAPPER2, DPR2, bA503C | dishevelled binding antagonist of beta catenin 2 |
| EHD2 | PAST2 | EH domain containing 2 |
| PARVB | CGI-56 | parvin beta |
| PYGB | GPBB | glycogen phosphorylase B |
| RYBP | AAP1, APAP-1, DEDAF, YEAF1 | RING1 and YY1 binding protein |
| MIR128-2 | MIR128B, MIRN128-2, MIRN128B, | microRNA 128-2 |
| USP28 | | ubiquitin specific peptidase 28 |
| TNS1 | MST091, MST122, MST127, MSTP0 | tensin 1 |
| BVES | CARICK, HBVES, LGMD2X, LGMDR2 | blood vessel epicardial substance |
| SQLE | | squalene epoxidase |
| ADAMTS1 | ADAMTS21, KNO2, MMCAT | ADAM metallopeptidase with thrombospondin type 1 motif 18 |
| MIR92B | MIRN92B, hsa-mir-92b, mir-92b | microRNA 92b |
| SKA2 | FAM33A | spindle and kinetochore associated complex subunit 2 |
| ANKS1B | AIDA, AIDA-1, ANKS2, EB-1, EB1, ca | ankyrin repeat and sterile alpha motif domain containing 1B |
| PDLIM2 | MYSTIQUE, SLIM | PDZ and LIM domain 2 |
| FRK | GTK, PTK5, RAK | fyn related Src family tyrosine kinase |
| DRG1 | NEDD3 | developmentally regulated GTP binding protein 1 |
| CDH4 | CAD4, R-CAD, RCAD | cadherin 4 |

| | | |
|---|---|---|
| B4GALNT1 | GALGT, GALNACT, GalNAc-T, SPG2 | beta-1,4-N-acetyl-galactosaminyltransferase 1 |
| MIRLET7D | LET7D, MIRNLET7D, hsa-let-7d, let- | microRNA let-7d |
| SNHG16 | Nbla10727, Nbla12061, ncRAN | small nucleolar RNA host gene 16 |
| CXCL17 | DMC, Dcip1, UNQ473, VCC-1, VCC1 | C-X-C motif chemokine ligand 17 |
| KLK15 | ACO, HSRNASPH | kallikrein related peptidase 15 |
| GLRX3 | GLRX4, GRX3, GRX4, PICOT, TXNL2, | glutaredoxin 3 |
| AJUBA | JUB | ajuba LIM protein |
| HOXC8 | HOX3, HOX3A | homeobox C8 |
| PTPN9 | MEG2, PTPMEG2 | protein tyrosine phosphatase non-receptor type 9 |
| MIR1290 | MIRN1290, hsa-mir-1290 | microRNA 1290 |
| SYNPO2 | | synaptopodin 2 |
| CDK10 | ALSAS, PISSLRE | cyclin dependent kinase 10 |
| LINC0067 | ERRLR01, HI-LNC75, HILNC75, LUC | long intergenic non-protein coding RNA 673 |
| BTF3 | BETA-NACa, BTF3b, NACB, BTF3 | basic transcription factor 3 |
| FZD8 | FZ-8, hFZ8 | frizzled class receptor 8 |
| GCNT2 | CCAT, CTRCT13C, GCNT5, IGNT, II, | glucosaminyl (N-acetyl) transferase 2 (I blood group) |
| AKAP4 | AKAP 82, AKAP-4, AKAP82, CT99, F | A-kinase anchoring protein 4 |
| MAGEA11 | CT1.11, MAGE-11, MAGE11, MAGE | MAGE family member A11 |
| MMP21 | HTX7, MMP-21 | matrix metallopeptidase 21 |
| MORC2 | CMT2Z, ZCW3, ZCWCC1 | MORC family CW-type zinc finger 2 |
| PDLIM1 | CLIM1, CLP-36, CLP36, HEL-S-112, | PDZ and LIM domain 1 |
| ADGRL3 | CIRL3, CL3, LEC3, LPHN3 | adhesion G protein-coupled receptor L3 |
| NUSAP1 | ANKT, BM037, LNP, NUSAP, PRO03 | nucleolar and spindle associated protein 1 |
| GALNT6 | GALNAC-T6, GalNAcT6 | polypeptide N-acetylgalactosaminyltransferase 6 |
| SPEN | HIAA0929, MINT, RBM15C, SHARP | spen family transcriptional repressor |
| CNMD | BRICD3, CHM-I, CHM1, LECT1, MYE | chondromodulin |
| TNFAIP1 | B12, B61, BTBD34, EDP1, hBACURD | TNF alpha induced protein 1 |
| P4HA2 | MYP25 | prolyl 4-hydroxylase subunit alpha 2 |
| MIR30C2 | MIRN30C2, mir-30c-2 | microRNA 30c-2 |
| LZTS1 | F37, FEZ1 | leucine zipper tumor suppressor 1 |
| TNFRSF21 | BM-018, CD358, DR6 | TNF receptor superfamily member 21 |
| TRIM59 | IFT80L, MRF1, RNF104, TRIM57, TS | tripartite motif containing 59 |
| FAM3C | GS3786, ILEI | FAM3 metabolism regulating signaling molecule C |
| MIR874 | MIRN874, hsa-mir-874, mir-874 | microRNA 874 |
| ZMYND8 | PRKCBP1, PRO2893, RACK7 | zinc finger MYND-type containing 8 |
| FBLN2 | | fibulin 2 |
| PLAGL2 | ZNF900 | PLAG1 like zinc finger 2 |
| FGFBP1 | FGF-BP, FGF-BP1, FGFBP, FGFBP-1, | fibroblast growth factor binding protein 1 |
| AFAP1 | AFAP, AFAP-11010, AFAP1 | actin filament associated protein 1 |
| PREX2 | DEP.2, DEPDC2, P-REX2, PPP1R129 | phosphatidylinositol-3,4,5-trisphosphate dependent Rac exchan |
| MIR376C | MIR368, MIRN368, MIRN376C, hsa | microRNA 376c |
| NORAD | LINC00657 | non-coding RNA activated by DNA damage |
| RASSF10 | | Ras association domain family member 10 |
| TNFAIP2 | B94, EXOC3L3 | TNF alpha induced protein 2 |
| DLEC1 | CFAP81, DLC-1, DLC1, F56, FAP81 | DLEC1 cilia and flagella associated protein |
| ADGRB1 | BAI1, GDAIF | adhesion G protein-coupled receptor B1 |
| FAIM2 | LFG, LFG2, NGP35, NMP35, TMBIM | Fas apoptotic inhibitory molecule 2 |
| MPZL1 | MPZL1b, PZR, PZR1b, PZRa, PZRb | myelin protein zero like 1 |
| CDR1-AS | CDR1NAT, CDR1as, ciRS-7 | CDR1 antisense RNA |
| PPM1F | CAMKP, CaMKPase, FEM-2, POPX2 | protein phosphatase, Mg2+/Mn2+ dependent 1F |
| SIX2 | | SIX homeobox 2 |

| | | |
|---|---|---|
| UBAP2L | NICE-4, NICE4 | ubiquitin associated protein 2 like |
| MCTS1 | MCT-1, MCT1 | MCTS1 re-initiation and release factor |
| DUSP2 | PAC-1, PAC1 | dual specificity phosphatase 2 |
| CHST11 | C4ST, C4ST-1, C4ST1, HSA269537, ( | carbohydrate sulfotransferase 11 |
| ERAS | HRAS2, HRASP | ES cell expressed Ras |
| WSB1 | SWIP1, WSB-1 | WD repeat and SOCS box containing 1 |
| PBXIP1 | HPIP | PBX homeobox interacting protein 1 |
| RHBDD1 | RHBDL4, RRP4 | rhomboid domain containing 1 |
| MIR26A2 | MIRN26A2, mir-26a-2 | microRNA 26a-2 |
| PRICKLE1 | EPM1B, RILP | prickle planar cell polarity protein 1 |
| MIR1271 | MIRN1271, hsa-mir-1271 | microRNA 1271 |
| MIR7-2 | MIRN7-2, hsa-mir-7-2, mir-7-2 | microRNA 7-2 |
| PIWIL4 | HIWI2, MIWI2 | piwi like RNA-mediated gene silencing 4 |
| MIR4435- | AGD2, LINC00978, MIR4435-1HG, I | MIR4435-2 host gene |
| MIR452 | MIRN452, hsa-mir-452, mir-452 | microRNA 452 |
| VPS37A | HCRP1, PQBP2, SPG53 | VPS37A subunit of ESCRT-I |
| OIP5 | 5730547N13Rik, CT86, LINT-25, MI | Opa interacting protein 5 |
| SOSTDC1 | CDA019, DAND7, ECTODIN, USAG1 | sclerostin domain containing 1 |
| GBP2 | | guanylate binding protein 2 |
| ADAM23 | MDC-3, MDC3 | ADAM metallopeptidase domain 23 |
| SAP30 | | Sin3A associated protein 30 |
| CAVIN2 | PS-p68, SDPR, SDR, cavin-2 | caveolae associated protein 2 |
| MIR532 | MIRN532, hsa-mir-532, mir-532 | microRNA 532 |
| PTBP3 | ROD1 | polypyrimidine tract binding protein 3 |
| SMARCAD | ADERM, BASNS, ETL1, HEL1, HRZ | SWI/SNF-related, matrix-associated actin-dependent regulator c |
| ALKBH5 | ABH5, OFOXD, OFOXD1 | alkB homolog 5, RNA demethylase |
| MIR32 | MIRN32, hsa-mir-32, miR-32, miRN | microRNA 32 |
| MIR7-3 | MIRN7-3, hsa-mir-7-3, mir-7-3 | microRNA 7-3 |
| HES6 | C-HAIRY1, HES-6, bHLHb41, bHLHc | hes family bHLH transcription factor 6 |
| NCKAP1 | HEM2, NAP1, NAP125, p125Nap1 | NCK associated protein 1 |
| NKD2 | Naked2 | NKD inhibitor of WNT signaling pathway 2 |
| MIR133A2 | MIRN133A2, mir-133a-2 | microRNA 133a-2 |
| EVL | RNB6 | Enah/Vasp-like |
| CASC9 | ESCCAL-1, ESSCAL1, LINC00981, lin | cancer susceptibility 9 |
| MIR411 | MIRN411, hsa-mir-411, mir-411 | microRNA 411 |
| SYNJ2 | INPP5H | synaptojanin 2 |
| MIR498 | MIRN498, hsa-mir-498, mir-498 | microRNA 498 |
| ATP6V1C1 | ATP6C, ATP6D, VATC, Vma5 | ATPase H+ transporting V1 subunit C1 |
| CRNN | C1orf10, DRC1, PDRC1, SEP53 | cornulin |
| MIIP | IIP45 | migration and invasion inhibitory protein |
| PCDH8 | ARCADLIN, PAPC | protocadherin 8 |
| LLGL2 | HGL, Hugl-2, LGL2 | LLGL scribble cell polarity complex component 2 |
| SCARA5 | NET33, Tesr | scavenger receptor class A member 5 |
| APLF | APFL, C2orf13, PALF, Xip1, ZCCHH1 | aprataxin and PNKP like factor |
| SRCIN1 | P140, SNIP | SRC kinase signaling inhibitor 1 |
| SHCBP1 | PAL | SHC binding and spindle associated 1 |
| TNS3 | TEM6, TENS1 | tensin 3 |
| UPK2 | UP2, UPII | uroplakin 2 |
| CCNY | C10orf9, CBCP1, CCNX, CFP1 | cyclin Y |
| MLLT11 | AF1Q | MLLT11 transcription factor 7 cofactor |
| MIR448 | MIRN448, hsa-mir-448, miRNA448 | microRNA 448 |

| | | |
|---|---|---|
| MIR744 | MIRN744, hsa-mir-744, mir-744 | microRNA 744 |
| PITPNM1 | DRES9, NIR2, PITPNM, RDGB, RDGI | phosphatidylinositol transfer protein membrane associated 1 |
| DDX43 | CT13, HAGE | DEAD-box helicase 43 |
| GHET1 | lncRNA-GHET1 | gastric carcinoma proliferation enhancing transcript 1 |
| ATG10 | APG10, APG10L, pp12616 | autophagy related 10 |
| MIR331 | MIRN331, hsa-mir-331, mir-331 | microRNA 331 |
| PKMYT1 | MYT1, PPP1R126 | protein kinase, membrane associated tyrosine/threonine 1 |
| CUEDC2 | C10orf66, bA18I14.5 | CUE domain containing 2 |
| TLK2 | HsHPK, MRD57, PKU-ALPHA | tousled like kinase 2 |
| MIR151A | MIR151, MIRN151, hsa-mir-151, hs | microRNA 151a |
| TRIM14 | | tripartite motif containing 14 |
| GPR132 | G2A | G protein-coupled receptor 132 |
| MIRLET7E | LET7E, MIRNLET7E, hsa-let-7e, let-7 | microRNA let-7e |
| CPNE3 | CPN3, PRO1071 | copine 3 |
| ST6GALNA | SAITL1, SIAT7, SIAT7B, SIATL1, ST6( | ST6 N-acetylgalactosaminide alpha-2,6-sialyltransferase 2 |
| MIR493 | MIRN493, hsa-mir-493, mir-493 | microRNA 493 |
| ANXA8 | ANX8, CH17-360D5.2 | annexin A8 |
| MIR539 | MIRN539, hsa-mir-539, mir-539 | microRNA 539 |
| FOXL1 | FKH6, FKHL11, FREAC7 | forkhead box L1 |
| CRB3 | | crumbs cell polarity complex component 3 |
| SNHG15 | C7orf40, Linc-Myo1g, MYO1GUT | small nucleolar RNA host gene 15 |
| MIR339 | MIRN339, hsa-mir-339, mir-339 | microRNA 339 |
| CHRDL1 | CHL, MGC1, MGCN, NRLN1, VOPT, | chordin like 1 |
| MIR362 | MIRN362, hsa-mir-362, mir-362 | microRNA 362 |
| KIF26B | | kinesin family member 26B |
| MT1X | MT-1l, MT1 | metallothionein 1X |
| LFNG | SCDO3 | LFNG O-fucosylpeptide 3-beta-N-acetylglucosaminyltransferase |
| NKILA | | NF-kappaB interacting lncRNA |
| MIR671 | MIRN671, hsa-mir-671, mir-671 | microRNA 671 |
| MIR376A1 | MIR376A-1, MIRN376A, MIRN376A | microRNA 376a-1 |
| LINP1 | | lncRNA in non-homologous end joining pathway 1 |
| RGS20 | RGSZ1, ZGAP1, g(z)GAP, gz-GAP | regulator of G protein signaling 20 |
| MIR379 | MIRN379, hsa-mir-379, mir-379 | microRNA 379 |
| ARID4A | RBBP-1, RBBP1, RBP-1, RBP1 | AT-rich interaction domain 4A |
| PCDH7 | BH-Pcdh, BHPCDH, PPP1R120 | protocadherin 7 |
| MIR1297 | MIRN1297, hsa-mir-1297, mir-1297 | microRNA 1297 |
| ST6GALNA | HSY11339, SIAT7A, ST6GalNAcI, ST | ST6 N-acetylgalactosaminide alpha-2,6-sialyltransferase 1 |
| CNN2 | | calponin 2 |
| IQGAP3 | | IQ motif containing GTPase activating protein 3 |
| SPANXC | CT11.3, CTp11, SPANX-C, SPANX-E, | SPANX family member C |
| ITGB3BP | CENP-R, CENPR, HSU37139, NRIF3, | integrin subunit beta 3 binding protein |
| MIR216B | MIRN216B, mir-216b | microRNA 216b |
| KATNB1 | KAT, LIS6 | katanin regulatory subunit B1 |
| SNHG7 | NCRNA00061 | small nucleolar RNA host gene 7 |
| ARPIN | C15orf38 | actin related protein 2/3 complex inhibitor |
| LHX6 | LHX6.1 | LIM homeobox 6 |
| MTBP | MDM2BP | MDM2 binding protein |
| FBXO22 | FBX22, FISTC1 | F-box protein 22 |
| MIR520B | MIRN520B | microRNA 520b |
| CBR3-AS1 | PlncRNA-1, PlncRNA1 | CBR3 antisense RNA 1 |
| GSE1 | CRHSP24, KIAA0182 | Gse1 coiled-coil protein |

| | | |
|---|---|---|
| GINS2 | HSPC037, PSF2, Pfs2 | GINS complex subunit 2 |
| CCL14 | CC-1, CC-3, CKB1, HCC-1, HCC-1(1- | C-C motif chemokine ligand 14 |
| MIR873 | MIRN873, hsa-mir-873, mir-873 | microRNA 873 |
| MAGEA9 | CT1.9, MAGE9 | MAGE family member A9 |
| ATG4A | APG4A, AUTL2 | autophagy related 4A cysteine peptidase |
| LY6K | CT97, HSJ001348, URLC10, ly-6K | lymphocyte antigen 6 family member K |
| MIR584 | MIRN584, hsa-mir-584 | microRNA 584 |
| EGFL6 | MAEG, W80 | EGF like domain multiple 6 |
| FOXJ2 | FHX | forkhead box J2 |
| RGMB | DRAGON | repulsive guidance molecule BMP co-receptor b |
| BCORL1 | BCoR-L1, CXorf10, SHUVER | BCL6 corepressor like 1 |
| LYNX1 | | Ly6/neurotoxin 1 |
| BMP10 | | bone morphogenetic protein 10 |
| ZNF326 | ZAN75, ZIRD, Zfp326, dJ871E2.1 | zinc finger protein 326 |
| CREB5 | CRE-BPA, CREB-5, CREBPA | cAMP responsive element binding protein 5 |
| FOXD2-AS1 | | FOXD2 adjacent opposite strand RNA 1 |
| MIR661 | MIRN661, hsa-mir-661 | microRNA 661 |
| ODAM | APIN | odontogenic, ameloblast associated |
| ARHGAP1 | MacGAP, SENEX, bA307O14.2 | Rho GTPase activating protein 18 |
| LZTFL1 | BBS17 | leucine zipper transcription factor like 1 |
| TROAP | TASTIN | trophinin associated protein |
| CRIP2 | CRIP, CRP2, ESP1 | cysteine rich protein 2 |
| THEM4 | CTMP | thioesterase superfamily member 4 |
| ILRUN | C6orf106, FP852, dJ391O22.4 | inflammation and lipid regulator with UBA-like and NBR1-like do |
| MIR548C | MIRN548C | microRNA 548c |
| MIR509-3 | MIRN509-3, mir-509-3 | microRNA 509-3 |
| NDUFB9 | B22, CI-B22, LYRM3, MC1DN24, U | NADH:ubiquinone oxidoreductase subunit B9 |
| AAMP | | angio associated migratory cell protein |
| ZKSCAN3 | ZF47, ZFP306, ZNF306, ZNF309, ZS | zinc finger with KRAB and SCAN domains 3 |
| LAD1 | LadA | ladinin 1 |
| CREB3L4 | AIBZIP, ATCE1, CREB3, CREB4, JAL, | cAMP responsive element binding protein 3 like 4 |
| NEWENTRY | | Record to support submission of GeneRIFs for a gene not in Gen |
| MIR592 | MIRN592, hsa-mir-592, mir-592 | microRNA 592 |
| MIR509-1 | MIRN509, MIRN509-1, hsa-mir-50 | microRNA 509-1 |
| NOP14 | C4orf9, NOL14, RES4-25, RES425, | NOP14 nucleolar protein |
| MIR500A | MIR500, MIRN500, hsa-mir-500, h | microRNA 500a |
| MLF2 | NTN4 | myeloid leukemia factor 2 |
| CETN1 | CEN1, CETN | centrin 1 |
| CT83 | CXorf61, KK-LC-1, KKLC1 | cancer/testis antigen 83 |
| ARPP19 | ARPP-16, ARPP-19, ARPP16, ENSA | cAMP regulated phosphoprotein 19 |
| TINAGL1 | ARG1, LCN7, LIECG3, TINAGRP | tubulointerstitial nephritis antigen like 1 |
| MIR655 | MIRN655, hsa-mir-655, mir-655 | microRNA 655 |
| MIR520C | MIRN520C | microRNA 520c |
| MIR1207 | MIRN1207, hsa-mir-1207 | microRNA 1207 |
| GAL3ST2 | GAL3ST-2, GP3ST | galactose-3-O-sulfotransferase 2 |
| ANXA13 | ANX13, ISA | annexin A13 |
| OTUB2 | C14orf137, OTB2, OTU2 | OTU deubiquitinase, ubiquitin aldehyde binding 2 |
| CACUL1 | C10orf46, CAC1 | CDK2 associated cullin domain 1 |
| MICAL2 | MICAL-2PV1, MICAL2PV2, MICAL2 | microtubule associated monooxygenase, calponin and LIM doma |
| SOHLH2 | SOSF2, SPATA28, TEB1, bHLHe81 | spermatogenesis and oogenesis specific basic helix-loop-helix 2 |
| CADM4 | IGSF4C, NECL4, Necl-4, TSLL2, syn | cell adhesion molecule 4 |

| | | |
|---|---|---|
| ESRP2 | RBM35B | epithelial splicing regulatory protein 2 |
| RPL39 | L39P42, RPL39_23_1806, RPL39 | ribosomal protein L39 |
| AIFM3 | AIFL | apoptosis inducing factor mitochondria associated 3 |
| GGNBP2 | DIF-3, DIF3, LCRG1, LZK1, ZFP403, Z | gametogenetin binding protein 2 |
| ARMCX1 | ALEX1, GASP7 | armadillo repeat containing X-linked 1 |
| PLPP5 | DPPL1, HTPAP, PPAPDC1B | phospholipid phosphatase 5 |
| TNN | TN-W, TNW | tenascin N |
| ERVK-10 | PR, Protease, Proteinase | endogenous retrovirus group K member 10 |
| MIR802 | MIRN802, hsa-mir-802 | microRNA 802 |
| EPHA10 | | EPH receptor A10 |
| SYNE3 | C14orf139, C14orf49, KASH3, LINC | spectrin repeat containing nuclear envelope family member 3 |
| TNFAIP8L3 | TIPE3 | TNF alpha induced protein 8 like 3 |
| CLIC3 | | chloride intracellular channel 3 |
| DUXAP9 | LINC01296 | double homeobox A pseudogene 9 |
| EPSTI1 | BRESI1 | epithelial stromal interaction 1 |
| MIR612 | MIRN612, hsa-mir-612 | microRNA 612 |
| MIR629 | MIRN629, hsa-mir-629, mir-629 | microRNA 629 |
| PITPNM3 | ACKR6, CORD5, NIR1, RDGBA3 | PITPNM family member 3 |
| CERNA2 | HOST2, lncRNA-HOST2 | competing endogenous lncRNA 2 for microRNA let-7b |
| STK17B | DRAK2 | serine/threonine kinase 17b |
| MIR432 | MIRN432, hsa-mir-432, mir-432 | microRNA 432 |
| CLDND1 | C3orf4, GENX-3745, Z38 | claudin domain containing 1 |
| LY6D | E48, Ly-6D | lymphocyte antigen 6 family member D |
| TNFAIP8L1 | TIPE1 | TNF alpha induced protein 8 like 1 |
| MIR660 | MIRN660, hsa-mir-660, mir-660 | microRNA 660 |
| PRRX2 | PMX2, PRX2 | paired related homeobox 2 |
| MIR374B | MIRN374B, mir-374b | microRNA 374b |
| MIR577 | MIRN577, hsa-mir-577, mir-577 | microRNA 577 |
| RNASEH2C | AGS3, AYP1 | ribonuclease H2 subunit C |
| ADGRF5 | GPR116, KPG_001 | adhesion G protein-coupled receptor F5 |
| MAP7D3 | MDP3 | MAP7 domain containing 3 |
| RERG | | RAS like estrogen regulated growth inhibitor |
| TMSB15A | TMSB15, TMSB15B, TMSL8, TMSNI | thymosin beta 15a |
| RUSC1 | NESCA | RUN and SH3 domain containing 1 |
| MIR770 | MIRN770, hsa-mir-770 | microRNA 770 |
| TMEM88 | | transmembrane protein 88 |
| RBM47 | NET18 | RNA binding motif protein 47 |
| IRX2 | IRXA2 | iroquois homeobox 2 |
| RAB40B | RAR, SEC4L | RAB40B, member RAS oncogene family |
| RADIL | RASIP2 | Rap associating with DIL domain |
| MIR520G | MIRN520G, mir-520g | microRNA 520g |
| PAGR1 | C16orf53, GAS, PA1 | PAXIP1 associated glutamate rich protein 1 |
| KIF3C | | kinesin family member 3C |
| FBXL14 | Fbl14 | F-box and leucine rich repeat protein 14 |
| SEMA6B | SEM-SEMA-Y, SEMA-VIB, SEMAN, s | semaphorin 6B |
| MIR520F | MIRN520F, mir-520f | microRNA 520f |
| MIR1915 | MIRN1915, hsa-mir-1915 | microRNA 1915 |
| UBR7 | C14orf130 | ubiquitin protein ligase E3 component n-recognin 7 |
| MIR564 | MIRN564, hsa-mir-564 | microRNA 564 |
| TTC9 | TTC9A | tetratricopeptide repeat domain 9 |
| LINC00052 | NCRNA00052, TMEM83 | long intergenic non-protein coding RNA 52 |

| | | |
|---|---|---|
| MOSPD2 | | motile sperm domain containing 2 |
| MIR519C | MIRN519C, mir-519c | microRNA 519c |
| MIR644A | MIR644, MIRN644, hsa-mir-644, hs | microRNA 644a |
| MIR105-2 | MIRN105-2, mir-105-2 | microRNA 105-2 |
| MIR645 | MIRN645, hsa-mir-645 | microRNA 645 |
| MIR1266 | MIRN1266, hsa-mir-1266, mir-126 | microRNA 1266 |
| MIR526B | MIRN526B | microRNA 526b |
| MIR1204 | hsa-mir-1204 | microRNA 1204 |
| DLK2 | DLK-2, EGFL9 | delta like non-canonical Notch ligand 2 |
| SNORD13 | MIR3607, mir-3607 | small nucleolar RNA, C/D box 138 |
| MIR515-1 | MIRN515-1 | microRNA 515-1 |
| MIR4319 | | microRNA 4319 |
| LINC01638 | | long intergenic non-protein coding RNA 1638 |
| MIR3178 | mir-3178 | microRNA 3178 |
| LNCNEF | LINC01384, lncRNA-NEF | lncRNA neighboring enhancer of FOXA2 |
| IRAIN | IGF1R-AS | IGF1R antisense imprinted non-protein coding RNA |
| MIR509-2 | MIRN509-2 | microRNA 509-2 |
| MIR4282 | | microRNA 4282 |
| MIR4480 | | microRNA 4480 |
| MIR4428 | | microRNA 4428 |
| COX2 | | cytochrome c oxidase subunit II |

Supplementary Table 3. Potential therapeutic target genes of tangeretin (PTs)

| No | Gene symbol |
|---|---|
| 1 | CASP3 |
| 2 | BCL2 |
| 3 | CYP3A4 |
| 4 | ABCB1 |
| 5 | AKT1 |
| 6 | HMOX1 |
| 7 | IL6 |
| 8 | MAPK8 |
| 9 | BCL2L1 |
| 10 | MAPK3 |
| 11 | PTGS2 |
| 12 | CASP9 |
| 13 | RELA |
| 14 | PIK3CA |
| 15 | JAG1 |
| 16 | CDH1 |
| 17 | TP53 |
| 18 | NOTCH1 |
| 19 | CCNB1 |
| 20 | PRDX6 |
| 21 | MCL1 |
| 22 | SOD2 |
| 23 | INS |
| 24 | IL10 |
| 25 | CYP1A1 |
| 26 | MBD2 |
| 27 | IL17A |
| 28 | CASP8 |
| 29 | VEGFA |
| 30 | PCNA |
| 31 | CAT |
| 32 | STAT3 |
| 33 | CCL2 |
| 34 | PER2 |
| 35 | MPO |
| 36 | NFKB1 |
| 37 | XBP1 |
| 38 | MTOR |
| 39 | IL23A |
| 40 | RETN |
| 41 | XIAP |
| 42 | NQO1 |
| 43 | CD36 |
| 44 | CDKN1A |

| | |
|---|---|
| 45 | MAPK1 |
| 46 | GJA1 |
| 47 | FOXP3 |
| 48 | CD4 |
| 49 | JAK2 |
| 50 | MMP9 |
| 51 | ABCG2 |
| 52 | PRKAA2 |
| 53 | G6PD |
| 54 | CCND1 |
| 55 | JUN |
| 56 | ADIPOQ |
| 57 | CTNNB1 |
| 58 | MAPK14 |

Supplementary Table 4. KEGG pathway enrichement analysis of the PTs

| No | Gene Set | Description | Size | Expect | Ratio |
|---|---|---|---|---|---|
| 1 | hsa01521 | EGFR tyrosine kinase inhibit | 79 | 0.60844 | 18.079 |
| 2 | hsa01522 | Endocrine resistance | 98 | 0.75478 | 19.873 |
| 3 | hsa01523 | Antifolate resistance | 31 | 0.23876 | 16.753 |
| 4 | hsa01524 | Platinum drug resistance | 73 | 0.56223 | 21.343 |
| 5 | hsa04010 | MAPK signaling pathway | 295 | 2.272 | 5.2816 |
| 6 | hsa04012 | ErbB signaling pathway | 85 | 0.65466 | 12.22 |
| 7 | hsa04014 | Ras signaling pathway | 232 | 1.7868 | 5.5965 |
| 8 | hsa04015 | Rap1 signaling pathway | 206 | 1.5866 | 5.6726 |
| 9 | hsa04024 | cAMP signaling pathway | 199 | 1.5327 | 5.2197 |
| 10 | hsa04062 | Chemokine signaling pathwa | 189 | 1.4556 | 6.1828 |
| 11 | hsa04064 | NF-kappa B signaling pathw | 95 | 0.73167 | 8.2004 |
| 12 | hsa04066 | HIF-1 signaling pathway | 100 | 0.77018 | 18.177 |
| 13 | hsa04068 | FoxO signaling pathway | 132 | 1.0166 | 15.738 |
| 14 | hsa04071 | Sphingolipid signaling pathw | 118 | 0.90882 | 11.003 |
| 15 | hsa04115 | p53 signaling pathway | 72 | 0.55453 | 16.23 |
| 16 | hsa04137 | Mitophagy | 65 | 0.50062 | 9.9876 |
| 17 | hsa04140 | Autophagy | 128 | 0.98583 | 10.144 |
| 18 | hsa04150 | mTOR signaling pathway | 151 | 1.163 | 6.019 |
| 19 | hsa04151 | PI3K-Akt signaling pathway | 354 | 2.7264 | 6.9688 |
| 20 | hsa04152 | AMPK signaling pathway | 120 | 0.92422 | 8.656 |
| 21 | hsa04210 | Apoptosis | 136 | 1.0474 | 15.275 |
| 22 | hsa04211 | Longevity regulating pathwa | 89 | 0.68546 | 16.048 |
| 23 | hsa04213 | Longevity regulating pathwa | 62 | 0.47751 | 14.659 |
| 24 | hsa04215 | Apoptosis | 32 | 0.24646 | 28.402 |
| 25 | hsa04218 | Cellular senescence | 160 | 1.2323 | 10.549 |
| 26 | hsa04310 | Wnt signaling pathway | 146 | 1.1245 | 4.4466 |
| 27 | hsa04370 | VEGF signaling pathway | 59 | 0.45441 | 17.605 |
| 28 | hsa04371 | Apelin signaling pathway | 137 | 1.0552 | 7.5819 |
| 29 | hsa04380 | Osteoclast differentiation | 128 | 0.98583 | 9.1293 |
| 30 | hsa04510 | Focal adhesion | 199 | 1.5327 | 7.177 |
| 31 | hsa04550 | Signaling pathways regulatir | 139 | 1.0706 | 7.4728 |
| 32 | hsa04611 | Platelet activation | 123 | 0.94732 | 5.278 |
| 33 | hsa04620 | Toll-like receptor signaling p | 104 | 0.80099 | 13.733 |
| 34 | hsa04621 | NOD-like receptor signaling | 168 | 1.2939 | 10.047 |
| 35 | hsa04623 | Cytosolic DNA-sensing pathv | 63 | 0.48522 | 6.1828 |
| 36 | hsa04625 | C-type lectin receptor signal | 104 | 0.80099 | 17.478 |
| 37 | hsa04630 | JAK-STAT signaling pathway | 162 | 1.2477 | 10.419 |
| 38 | hsa04657 | IL-17 signaling pathway | 93 | 0.71627 | 19.546 |
| 39 | hsa04658 | Th1 and Th2 cell differentiat | 92 | 0.70857 | 15.524 |
| 40 | hsa04659 | Th17 cell differentiation | 107 | 0.8241 | 18.202 |
| 41 | hsa04660 | T cell receptor signaling patl | 101 | 0.77788 | 12.855 |

| | | | | | |
|---|---|---|---|---|---|
| 42 | hsa04662 | B cell receptor signaling pat | 71 | 0.54683 | 12.801 |
| 43 | hsa04664 | Fc epsilon RI signaling pathv | 68 | 0.52372 | 11.456 |
| 44 | hsa04668 | TNF signaling pathway | 110 | 0.8472 | 18.886 |
| 45 | hsa04722 | Neurotrophin signaling path | 119 | 0.91652 | 12.002 |
| 46 | hsa04726 | Serotonergic synapse | 115 | 0.88571 | 4.5161 |
| 47 | hsa04910 | Insulin signaling pathway | 137 | 1.0552 | 7.5819 |
| 48 | hsa04914 | Progesterone-mediated ooc | 99 | 0.76248 | 10.492 |
| 49 | hsa04915 | Estrogen signaling pathway | 137 | 1.0552 | 6.6341 |
| 50 | hsa04917 | Prolactin signaling pathway | 70 | 0.53913 | 22.258 |
| 51 | hsa04919 | Thyroid hormone signaling p | 116 | 0.89341 | 11.193 |
| 52 | hsa04920 | Adipocytokine signaling patl | 69 | 0.53143 | 18.817 |
| 53 | hsa04926 | Relaxin signaling pathway | 130 | 1.0012 | 10.986 |
| 54 | hsa04930 | Type II diabetes mellitus | 46 | 0.35428 | 19.758 |
| 55 | hsa04931 | Insulin resistance | 107 | 0.8241 | 13.348 |
| 56 | hsa04932 | Non-alcoholic fatty liver dise | 149 | 1.1476 | 11.328 |
| 57 | hsa04933 | AGE-RAGE signaling pathwa | 99 | 0.76248 | 22.296 |
| 58 | hsa04976 | Bile secretion | 71 | 0.54683 | 5.4862 |
| 59 | hsa05014 | Amyotrophic lateral sclerosi | 51 | 0.39279 | 17.821 |
| 60 | hsa05120 | Epithelial cell signaling in He | 68 | 0.52372 | 11.456 |
| 61 | hsa05131 | Shigellosis | 65 | 0.50062 | 11.985 |
| 62 | hsa05132 | Salmonella infection | 86 | 0.66236 | 12.078 |
| 63 | hsa05133 | Pertussis | 76 | 0.58534 | 18.793 |
| 64 | hsa05134 | Legionellosis | 55 | 0.4236 | 14.164 |
| 65 | hsa05140 | Leishmaniasis | 74 | 0.56994 | 15.791 |
| 66 | hsa05142 | Chagas disease (American tı | 102 | 0.78559 | 16.548 |
| 67 | hsa05145 | Toxoplasmosis | 112 | 0.8626 | 18.548 |
| 68 | hsa05146 | Amoebiasis | 96 | 0.73938 | 8.115 |
| 69 | hsa05152 | Tuberculosis | 179 | 1.3786 | 10.88 |
| 70 | hsa05160 | Hepatitis C | 131 | 1.0089 | 10.903 |
| 71 | hsa05161 | Hepatitis B | 144 | 1.1091 | 17.132 |
| 72 | hsa05162 | Measles | 132 | 1.0166 | 8.8527 |
| 73 | hsa05163 | Human cytomegalovirus infe | 225 | 1.7329 | 11.541 |
| 74 | hsa05164 | Influenza A | 171 | 1.317 | 9.8708 |
| 75 | hsa05165 | Human papillomavirus infec | 339 | 2.6109 | 6.5111 |
| 76 | hsa05166 | Human T-cell leukemia virus | 255 | 1.964 | 7.6376 |
| 77 | hsa05167 | Kaposi sarcoma-associated l | 186 | 1.4325 | 15.357 |
| 78 | hsa05168 | Herpes simplex infection | 185 | 1.4248 | 7.7202 |
| 79 | hsa05169 | Epstein-Barr virus infection | 201 | 1.5481 | 10.335 |
| 80 | hsa05170 | Human immunodeficiency v | 212 | 1.6328 | 10.412 |
| 81 | hsa05200 | Pathways in cancer | 524 | 4.0358 | 7.6813 |
| 82 | hsa05202 | Transcriptional misregulatio | 186 | 1.4325 | 6.2825 |
| 83 | hsa05203 | Viral carcinogenesis | 201 | 1.5481 | 7.7516 |
| 84 | hsa05205 | Proteoglycans in cancer | 198 | 1.525 | 9.1806 |
| 85 | hsa05206 | MicroRNAs in cancer | 150 | 1.1553 | 14.715 |
| 86 | hsa05210 | Colorectal cancer | 86 | 0.66236 | 21.137 |
| 87 | hsa05211 | Renal cell carcinoma | 69 | 0.53143 | 13.172 |

| | | | | | |
|---|---|---|---|---|---|
| 88 | hsa05212 | Pancreatic cancer | 75 | 0.57764 | 25.968 |
| 89 | hsa05213 | Endometrial cancer | 58 | 0.44671 | 22.386 |
| 90 | hsa05214 | Glioma | 71 | 0.54683 | 14.63 |
| 91 | hsa05215 | Prostate cancer | 97 | 0.74708 | 20.078 |
| 92 | hsa05216 | Thyroid cancer | 37 | 0.28497 | 24.564 |
| 93 | hsa05218 | Melanoma | 72 | 0.55453 | 14.427 |
| 94 | hsa05219 | Bladder cancer | 41 | 0.31577 | 25.334 |
| 95 | hsa05220 | Chronic myeloid leukemia | 76 | 0.58534 | 17.084 |
| 96 | hsa05221 | Acute myeloid leukemia | 66 | 0.50832 | 21.64 |
| 97 | hsa05222 | Small cell lung cancer | 92 | 0.70857 | 18.347 |
| 98 | hsa05223 | Non-small cell lung cancer | 66 | 0.50832 | 17.705 |
| 99 | hsa05224 | Breast cancer | 147 | 1.1322 | 10.599 |
| 100 | hsa05225 | Hepatocellular carcinoma | 167 | 1.2862 | 9.3298 |
| 101 | hsa05226 | Gastric cancer | 148 | 1.1399 | 10.528 |
| 102 | hsa05230 | Central carbon metabolism | 65 | 0.50062 | 13.983 |
| 103 | hsa05231 | Choline metabolism in canc | 99 | 0.76248 | 9.1806 |
| 104 | hsa05321 | Inflammatory bowel disease | 65 | 0.50062 | 17.978 |
| 105 | hsa05323 | Rheumatoid arthritis | 90 | 0.69316 | 8.656 |
| 106 | hsa05418 | Fluid shear stress and ather | 138 | 1.0629 | 15.054 |

| P Value | FDR |
|---|---|
| 1.22E-11 | 1.31E-10 |
| 2.22E-16 | 7.24E-15 |
| 8.5079E-05 | 0.00028594 |
| 1.61E-13 | 2.28E-12 |
| 1.7886E-06 | 7.2886E-06 |
| 2.25E-07 | 1.0471E-06 |
| 8.8364E-06 | 0.00003389 |
| 2.3564E-05 | 8.2602E-05 |
| 0.00012575 | 0.0004141 |
| 1.1808E-05 | 0.00004261 |
| 8.1395E-05 | 0.0002764 |
| 1.22E-14 | 2.21E-13 |
| 1.22E-15 | 3.06E-14 |
| 1.64E-08 | 8.62E-08 |
| 2.86E-09 | 1.80E-08 |
| 0.00013128 | 0.00042799 |
| 3.60E-08 | 1.83E-07 |
| 0.00014328 | 0.00045794 |
| 5.81E-12 | 6.77E-11 |
| 3.1913E-06 | 1.2844E-05 |
| 2.00E-15 | 4.34E-14 |
| 4.66E-11 | 4.75E-10 |
| 3.87E-07 | 0.00000173 |
| 3.17E-09 | 1.89E-08 |
| 1.45E-10 | 1.31E-09 |
| 0.0051139 | 0.014186 |
| 1.21E-08 | 6.56E-08 |
| 8.583E-06 | 0.00003331 |
| 4.61E-07 | 2.033E-06 |
| 2.48E-07 | 1.124E-06 |
| 9.5537E-06 | 3.5392E-05 |
| 0.0024485 | 0.0070018 |
| 2.61E-10 | 2.13E-09 |
| 2.68E-10 | 2.13E-09 |
| 0.012402 | 0.031341 |
| 2.16E-14 | 3.71E-13 |
| 1.70E-10 | 1.46E-09 |
| 4.22E-15 | 8.09E-14 |
| 6.75E-11 | 6.47E-10 |
| 1.11E-15 | 3.02E-14 |
| 3.57E-09 | 2.04E-08 |

| | |
|---|---|
| 9.93E-07 | 4.0982E-06 |
| 1.2025E-05 | 0.00004261 |
| 0 | 0 |
| 1.13E-09 | 7.39E-09 |
| 0.011595 | 0.029765 |
| 8.583E-06 | 0.00003331 |
| 7.36E-07 | 3.1172E-06 |
| 7.7646E-05 | 0.00026645 |
| 9.49E-14 | 1.55E-12 |
| 1.39E-08 | 7.42E-08 |
| 7.61E-11 | 7.09E-10 |
| 2.93E-09 | 1.80E-08 |
| 4.64E-08 | 2.32E-07 |
| 3.57E-10 | 2.64E-09 |
| 5.89E-11 | 5.81E-10 |
| 0 | 0 |
| 0.017097 | 0.042223 |
| 9.73E-08 | 4.67E-07 |
| 1.2025E-05 | 0.00004261 |
| 9.2369E-06 | 3.5014E-05 |
| 2.46E-07 | 1.124E-06 |
| 7.88E-12 | 8.86E-11 |
| 3.4394E-06 | 1.3674E-05 |
| 3.67E-09 | 2.07E-08 |
| 4.28E-13 | 5.81E-12 |
| 1.11E-16 | 4.52E-15 |
| 0.00008631 | 0.00028711 |
| 2.77E-12 | 3.34E-11 |
| 3.18E-09 | 1.89E-08 |
| 0 | 0 |
| 6.00E-07 | 2.5732E-06 |
| 0 | 0 |
| 3.35E-10 | 2.54E-09 |
| 2.91E-10 | 2.26E-09 |
| 4.45E-10 | 3.22E-09 |
| 0 | 0 |
| 1.18E-07 | 5.58E-07 |
| 1.01E-12 | 1.32E-11 |
| 1.51E-13 | 2.23E-12 |
| 0 | 0 |
| 1.0374E-05 | 3.8001E-05 |
| 2.75E-08 | 1.43E-07 |
| 1.66E-10 | 1.46E-09 |
| 4.44E-16 | 1.32E-14 |
| 1.33E-15 | 3.10E-14 |
| 8.15E-07 | 3.4075E-06 |

| | |
|---|---|
| 0 | 0 |
| 1.24E-11 | 1.31E-10 |
| 5.40E-08 | 2.67E-07 |
| 2.22E-16 | 7.24E-15 |
| 9.40E-09 | 5.20E-08 |
| 6.04E-08 | 2.94E-07 |
| 5.79E-10 | 4.10E-09 |
| 2.05E-10 | 1.71E-09 |
| 1.56E-12 | 1.96E-11 |
| 1.08E-13 | 1.68E-12 |
| 1.29E-09 | 8.24E-09 |
| 7.73E-10 | 5.36E-09 |
| 3.37E-09 | 1.96E-08 |
| 8.37E-10 | 5.68E-09 |
| 5.39E-07 | 2.3416E-06 |
| 9.4163E-06 | 3.5284E-05 |
| 1.12E-09 | 7.39E-09 |
| 6.0059E-05 | 0.00020829 |
| 2.55E-15 | |

Supplementary Table 5. Gene list enriched in breast cancer, TNF and PI3K signal

related to breast cancer

| User ID | Gene Symbo | Gene Name | Entrez Gene ID |
|---|---|---|---|
| AKT1 | AKT1 | AKT serine/tl | 207 |
| CCND1 | CCND1 | cyclin D1 | 595 |
| CDKN1A | CDKN1A | cyclin depen | 1026 |
| CTNNB1 | CTNNB1 | catenin beta | 1499 |
| JAG1 | JAG1 | jagged canor | 182 |
| JUN | JUN | Jun proto-on | 3725 |
| MAPK1 | MAPK1 | mitogen-acti | 5594 |
| MAPK3 | MAPK3 | mitogen-acti | 5595 |
| MTOR | MTOR | mechanistic t | 2475 |
| NOTCH1 | NOTCH1 | notch recept | 4851 |
| PIK3CA | PIK3CA | phosphatidyl | 5290 |
| TP53 | TP53 | tumor protei | 7157 |

related to TNF signaling pathway

| User ID | Gene Symbo | Gene Name | Entrez Gene ID |
|---|---|---|---|
| AKT1 | AKT1 | AKT serine/tl | 207 |
| CASP3 | CASP3 | caspase 3 | 836 |
| CASP8 | CASP8 | caspase 8 | 841 |
| CCL2 | CCL2 | C-C motif che | 6347 |
| IL6 | IL6 | interleukin 6 | 3569 |
| JAG1 | JAG1 | jagged canor | 182 |
| JUN | JUN | Jun proto-on | 3725 |
| MAPK1 | MAPK1 | mitogen-acti | 5594 |
| MAPK14 | MAPK14 | mitogen-acti | 1432 |
| MAPK3 | MAPK3 | mitogen-acti | 5595 |
| MAPK8 | MAPK8 | mitogen-acti | 5599 |
| MMP9 | MMP9 | matrix metal | 4318 |
| NFKB1 | NFKB1 | nuclear facto | 4790 |
| PIK3CA | PIK3CA | phosphatidyl | 5290 |
| PTGS2 | PTGS2 | prostaglandi | 5743 |
| RELA | RELA | RELA proto-c | 5970 |

related tothe PI3K signaling pathway

| User ID | Gene Symbo | Gene Name | Entrez Gene ID |
|---|---|---|---|
| AKT1 | AKT1 | AKT serine/tl | 207 |
| BCL2 | BCL2 | BCL2, apoptc | 596 |
| BCL2L1 | BCL2L1 | BCL2 like 1 | 598 |

| | | | |
|---|---|---|---|
| CASP9 | CASP9 | caspase 9 | 842 |
| CCND1 | CCND1 | cyclin D1 | 595 |
| CDKN1A | CDKN1A | cyclin depen | 1026 |
| IL6 | IL6 | interleukin 6 | 3569 |
| INS | INS | insulin | 3630 |
| JAK2 | JAK2 | Janus kinase | 3717 |
| MAPK1 | MAPK1 | mitogen-acti | 5594 |
| MAPK3 | MAPK3 | mitogen-acti | 5595 |
| MCL1 | MCL1 | MCL1, BCL2 f | 4170 |
| MTOR | MTOR | mechanistic t | 2475 |
| NFKB1 | NFKB1 | nuclear facto | 4790 |
| PIK3CA | PIK3CA | phosphatidyl | 5290 |
| PRKAA2 | PRKAA2 | protein kinas | 5563 |
| RELA | RELA | RELA proto-c | 5970 |
| TP53 | TP53 | tumor protei | 7157 |
| VEGFA | VEGFA | vascular end | 7422 |
